# Multiplex glycan bead array for high throughput and high content analyses of glycan binding proteins

**DOI:** 10.1038/s41467-017-02747-y

**Published:** 2018-01-17

**Authors:** Sharad Purohit, Tiehai Li, Wanyi Guan, Xuezheng Song, Jing Song, Yanna Tian, Lei Li, Ashok Sharma, Boying Dun, David Mysona, Sharad Ghamande, Bunja Rungruang, Richard D. Cummings, Peng George Wang, Jin-Xiong She

**Affiliations:** 1 Center for Biotechnology and Genomic Medicine, Medical College of Georgia, Augusta University, 1120 15th Street, Augusta, GA 30912 USA; 2 Department of Obstetrics and Gynecology, Medical College of Georgia, Augusta University, 1120 15th Street, Augusta, GA 30912 USA; 3 Department of Medical Laboratory, Imaging and Radiologic Sciences, College of Allied Health Sciences Augusta University, 1120 15th Street, Augusta, GA 30912 USA; 4 Department of Chemistry, Georgia State University, Atlanta, GA 30303 USA; 5 Department of Biochemistry, Emory University School of Medicine, Atlanta, GA 30322 USA; 6 Department of Surgery, Beth Israel Deaconess Medical Center, Harvard Medical School, Boston, MA 02115 USA

## Abstract

Glycan-binding proteins (GBPs) play critical roles in diverse cellular functions such as cell adhesion, signal transduction and immune response. Studies of the interaction between GBPs and glycans have been hampered by the availability of high throughput and high-content technologies. Here we report multiplex glycan bead array (MGBA) that allows simultaneous analyses of 384 samples and up to 500 glycans in a single assay. The specificity, sensitivity and reproducibility of MGBA are evaluated using 39 plant lectins, 13 recombinant anti-glycan antibodies, and mammalian GBPs. We demonstrate the utility of this platform by the analyses of natural anti-glycan IgM and IgG antibodies in 961 human serum samples and the discovery of anti-glycan antibody biomarkers for ovarian cancer. Our data indicate that the MGBA platform is particularly suited for large population-based studies that require the analyses of large numbers of samples and glycans.

## Abstract (web summary)

The low throughput or content of current methods for the analysis of glycans-glycan binding proteins (GBPs) interactions hampers their clinical applications. Here, the authors conjugate synthesized glycans to Luminex beads to detect GBPs and apply it for the discovery of ovarian cancer biomarkers.

## Introduction

Many cell surface and secretory proteins produced by mammalian cells are modified by glycans, which play an essential role in the maintenance of diverse cellular functions. Alterations to glycans, such as increased levels of truncation and branching as well as presence of unusual terminal sequences, may cause changes in various physiological and pathogenic processes, including oncogenic transformation^1–3^ and autoimmunity^4–6^. There are a large number of glycan-binding proteins (GBPs). The three main mammalian GBP families are C-type lectins, galectins and siglecs, which play critical roles in diverse cellular functions such as cell adhesion, signal transduction and immune response^6^. Other GBPs include proteins involved in mediating intracellular trafficking, bacterial adhesion molecules, bacterial toxins, viral GBPs and other microbial GBPs, which are important for pathogen-host interactions. A special category of GBPs is anti-glycan antibodies^7,8^, which play an essential role in various diseases, including autoimmune diseases, cancer, blood transfusion, organ transplants and responses to vaccines^5,9–11^. In addition to blood transfusion, anti-glycan antibodies may also have a tremendous potential as diagnostic and prognostic markers for other diseases such as cancer and autoimmunity^5,9–11^.

Despite the critical importance of GBPs in normal physiology and pathogenesis, progress in this field has been slow due to the lack of available glycans and adequate assay technologies. ELISA has been traditionally used to analyze GBPs, including anti-glycan antibodies. ELISA can analyze large numbers of samples but only for a small number of glycans. This obstacle was partially alleviated by advent of the glycan arrays on flat surface such as glass slides, which were mainly developed by the consortium of functional glycomics (CFG)^12,13^. Glycan arrays have been used in a variety of studies and have greatly enhanced our understanding of glycan functions^13,14^. For example, it was shown for the first time the presence of antibodies to a variety of simple sugars and structurally complex glycans^7,8^. However, the flat surface glycan array is technically challenging and cannot be easily made available to non-experts. It is difficult to translate flat surface microarray into a clinical tool due to the technical difficulties and long turn-around time of the assay. Furthermore, the flat surface array does not allow rapid analyses of large numbers of samples required by many studies. Therefore, there is still an urgent need for improved and affordable technologies that can analyze simultaneously large numbers of samples (high throughput) and large numbers of glycans (high content). Development of such a platform that can be used by all biologists and clinic technologists without sophisticated technical expertise will greatly speed up glycomic studies and the translation of glycomic discoveries into clinical tests for diagnosis of microbial response^15–18^, inflammation^10,19–21^ and cancers^5,22,23^. Earlier studies on development of suspension glycan array used a cumbersome procedure with unknown reproducibility and scalability^24–26^. Only a few glycans were used, of these reproducibility of the procedure was determined by using blood group A and B antigens on 45 serum samples^24–26^. Taken together the studies did not address the reproducibility, specificity and sensitivity of the glycan array to produce convincing data to demonstrate the utility of the platform^24–26^.

We report here the development of multiplex glycan bead array (MGBA), which is based on Luminex bead array technology^27^. To create the MGBA, we covalently attach one glycan per bead region, creating a panel of 184 beads corresponding to 184 glycans used in this study. This high throughput and high-content platform allows rapid and economic analyses of large numbers (thousands) of glycans and large number of samples (thousands to tens of thousands).

## Results

### Creation of MGBA version I

**Fig. 1:**
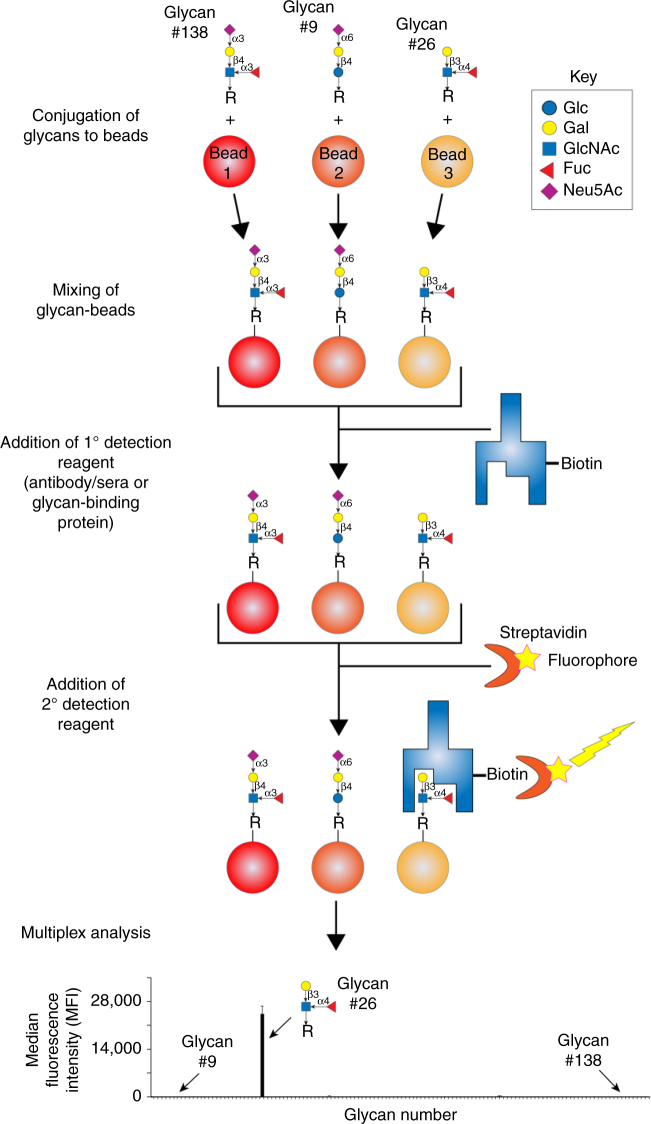
Design and workflow of MGBA. Each glycan was conjugated to one region specific Luminex bead, using 1-ethyl-3-(3-dimethylaminopropyl) carbodiimide hydrochloride. After blocking the beads with 1% BSA in PBS (w/v), the beads were probed with biotinylated lectins, anti-glycan antibodies and glycan-binding proteins. After washing, the bound lectins, anti-glycan antibodies and glycan-binding proteins were detected using phycoerythrin labelled streptavidin (SAPE). The unbound SAPE was removed by washing, and beads were resuspended in wash buffer. The beads were read in FLEXMAP 3D, as per settings defined in “Methods” secrion. The median fluorescence intensity data is then presented as mean+standard deviation of two replicates; each experiment was repeated three times

The overall design and workflow for MGBA is depicted in Fig. 1. The first task of establishing the MGBA was to determine the optimum conditions for glycan conjugation to Luminex beads. Towards this goal, we tested different conjugation strategies for covalent coupling of defined glycan structures to carboxylated Luminex beads (–COOH beads). We optimized the conjugation chemistry as well as the concentration of glycans required to achieve reproducible covalent attachment. We tried three different conjugation chemistries (Sulfo NHS-EDC, DMTMM and EDC), which have been described in the literature^28–30^, to covalently attach glycans to carboxyl microspheres. The Sulfo NHS-EDC conjugation method did not give us acceptable signal to noise ratio for any glycans used in our pilot studies and was not further pursued. DMTMM conjugation yielded good signals but was generally weaker than the data from the EDC conjugation (Supplementary Fig. 1). Therefore, the final protocol uses covalent attachment of glycan to beads using the EDC activation procedure by the formation of an amide bond. This procedure was used to generate conjugated beads for our first version of MGBA. Each glycan was conjugated to a specific bead type in duplicate to assess the quality of the conjugation, which was done by plant lectin binding based on their known binding affinities, discrepancy in plant lectin binding to the known glycans or between duplicate conjugations. Any glycan that did not meet these QC parameters was re-conjugated and retested using the same procedure.

To assess the reproducibility of conjugation over time, we performed four sets of conjugations on four different days (day 1, 2, 5, and 10). The reproducibility of the conjugation was assessed by detecting anti-glycan IgM in 48 serum samples within the same assay plate. Correlations between different conjugations are usually excellent (*r* > 0.96) (Supplementary Fig. 2a). Reproducibility of the assay over time was assessed by measuring anti-glycan IgM in 48 serum samples using the same batch of beads on six different days (1, 2, 5, 10, 15, and 28). We consistently observed high correlation values (*r* > 0.97) (Supplementary Fig. 2b). Comparing the data in Supplementary Fig. 2a vs. 2b, it is evident that there is more variability between different batches of beads compared to different assays using the same conjugation, although the batch-to-batch conjugations are reproducible.

Our glycan library contains a total of 184 glycans, of which 102 are aminoalkyl glycans synthesized in laboratory of Dr. Peng George Wang using combinatorial chemistry^31^ and 82 are AEAB functionalized glycans from Dr. Richard D. Cummings as part of the Consortium of Functional Glycomics (CFG) glycan array^32^. The glycans and their associated structures used in this version I of MGBA are listed in Supplementary Table 1. The glycan-conjugated beads can be mixed in a single well and assayed simultaneously or organized into subpanels at any desired combinations and assayed separately.

### Validation of MGBA by plant lectins

**Fig. 2:**
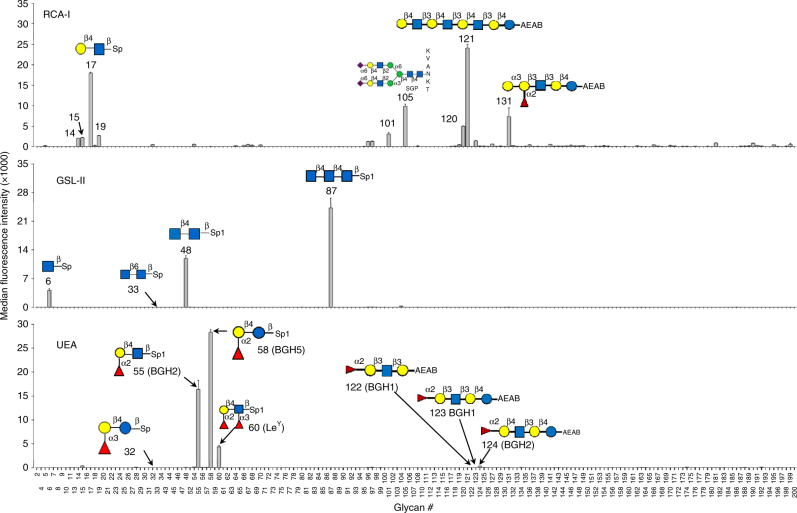
MGBA data using lectin as detection reagents. Bar charts show median fluorescence intensity (MFI) (on *y*-axis) for each of the 184 glycans by their order of glycan ID (on *x*-axis). Data for three representative lectins are shown and data for all other lectins are in Supplementary Figure 3. The data are presented as mean+standard deviation of two replicates; each experiment was repeated three times

In the first set of validation experiments for our MGBA platform, 39 biotinylated plant lectins (Supplementary Table 2) were used to probe each of the 184 glycan beads. As shown in Fig. 2 for three representative plant lectins, each plant lectin binds to a specific subset of glycans. Consistent with reported specificities^33^, RCA-I exhibited high levels of binding to glycans containing Gal-β4-GlcNAc motif present in several glycans (#17, 105 and 121), with the highest signal observed for glycan #121 containing tandem repeats of Gal-β4-GlcNAc. Glycan with similar motif such as Gal-β3-GlcNAc (#18) did not show any binding with RCA-I. Surprisingly, glycan #131 with an internal Gal-β3-GlcNAc motif [Gal-α3-(Fuc-α2)-Gal-β3-GlcNAc-β3-Gal-β4-Glc] showed the second highest binding. The binding of RCA-I observed in our MGBA is similar to that observed for RCA-I in the CFG array, where RCA-I had high signal levels for glycans containing Gal-β4-GlcNAc. As reported in previous studies^34^, RCA-I was also found to bind to Sia-α6-Gal-β4-GlcNAc-containing bi-antennary N-Glycan SGP (#105). However, no binding was observed for α6- and/or α3-linked mono- and/or di-sialylated forms of T-antigen (#65, 66, 67 and 68). Similarly, α6- and α3-linked mono-sialic acid or N-glycolylneuraminic acid present on Gal-β3-GlcNAc (#22–24)) or Gal-β4-Glc (#9 and 10) backbone also showed no binding.

GSL-II, a lectin from *Griffonia simplicifolia*, specifically binds to β-linked GlcNAc oligomers (#48 and #87) and shows low level of binding to a GlcNAc monomer (#6) (Fig. 2). The binding of GSL-II is specific to GlcNAc-β4-GlcNAc motif, since no binding was observed for similar motif GlcNAc-β6-GlcNAc (#33). The binding of GSL-II in our MGBA showed some difference compared to CFG array, where GSL-II binds to all glycans containing terminal and internal GlcNAc, with higher signals observed for tandem repeats of Gal-β4-GlcNAc-containing bi- and tri- antennary glycans. On our MGBA, GSL-II only binds to tandem repeats of Gal-β4-GlcNAc but does not bind to internal or terminal GlcNAc, suggesting higher specificity in our suspension array compared to glass arrays used by CFG.

As expected, the UEA lectin showed strong affinity towards blood group H type2 (BGH2) glycan (#55) and a BGH type 5 glycan (#58) but did not bind to blood group H type 1 (BGH1) glycans (#122 and 123) (Fig. 2). UEA also showed binding to Le^y^ (#60) that also contained the Fuc-α2-Gal-β4-GlcNAc motif. While UEA shows high signals for BGH2 glycan (#55), it does not bind to glycan #32, which only differs from glycan #55 by the fucose linkage. Furthermore, UEA showed low signal to a long chain glycan that contains the BGH2 motif (#124). Consistent with a previous report^34^, UEA did not show binding to other α2-Fuc containing glycans such as glycan #56 and #59. These results together suggest that UEA specifically binds to Fuc-α2-Gal-β4-GlcNAc (#55) or Fuc-α2-Gal-β4-Glc (#58) motifs but the binding can be reduced or abolished by the structure beyond the core motif as seen for glycan #60 and #124.

The net (background-subtracted) MFI values for all 39 lectins and 184 glycans are provided in Supplementary Data 1 and bar charts showing the binding activities for each lectin and each glycan are shown in Supplementary Fig. 3. Overall, the binding patterns for all lectins are largely consistent with previous knowledge. A total of 16 lectins did not show binding in this version of MGBA because specific glycans for eight lectins were not present in this MGBA (ECL, PHA-L, PHA-E, NPL, HHL, MPL, MAL-I and MAL-II) or possibly other technical issues such as poor quality of the lectins, poor quality of lectin labeling, low affinity binding, poor quality of the specific glycans, and inadequate binding conditions (SBA, BPL, ACL, SuConA, LTL, SJA, PTLII and BSIB4). One example for such issues is on AAL, which gives high background at a concentration of 5 µg/ml that is suitable for other lectins. However, the background for assays using 1 µg/ml of AAL was reduced to levels comparable to those observed for other lectins at 5 µg/ml. As expected, AAL binds specifically to glycans containing α-fucose in α-2, α-3 and α-4 linkages^34^.

To further test the specificity of the MGBA, competition assays were carried out for a number of plant lectins. Briefly, a simple sugar unconjugated to beads with specificity to certain lectins was added to the binding buffer before mixing with glycan-conjugated beads to determine the ability of the sugar to compete for binding with glycan-conjugated beads. Our data show that galactose can specifically block binding of RCA-I to beads conjugated with glycans containing galactose. Similarly, GlcNAc can specifically block binding of GSL-II to GlcNAc-containing glycans and fucose can specifically block binding of UEA and AAL to fucose-containing glycans (Supplementary Fig. 4). These results provide further evidence that plant lectins bind specially to the glycans conjugated to beads.

**Fig. 3:**
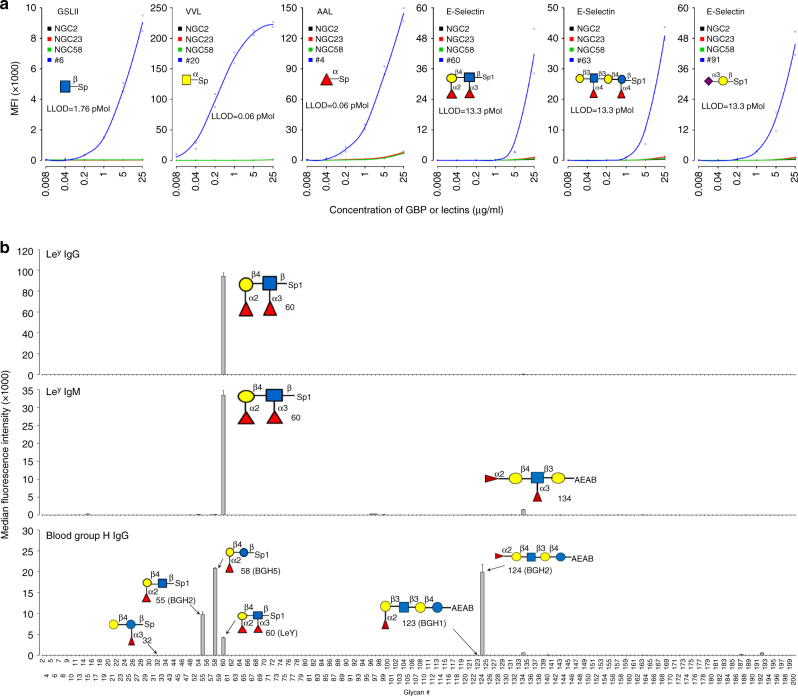
Evaluation of MGBA’s sensitivity and specificity. **a** Serial dilutions of lectins and GBPs were used to assess the lower limit of detection of MGBA. Lowest limit of detection values were 1.76pMol (GSL-II), 0.06pMol (VVL and AAL) and 13.3pMol (E-Selectin). **b** Specificity for anti-glycan antibodies. The data for three representative antibodies are shown here and the data for other antibodies are shown in Supplementary Fig. 6. The glycans with similar structures without binding are also presented in the figure to show specificity of the binding. Mean of two replicates is presented and error bars are standard deviations of the two replicates. Each experiment was repeated three times

We then proceeded to test the sensitivity of the MGBA assay by using serial dilutions of seven plant lectins (RCA-I, ConA, GSL-II, SuWGA, VVL, WFL, and AAL). The top panel of Fig. 3a shows results for five-fold serial dilutions for GSL-II, VVL and AAL, and the data for the other four lectins are presented in Supplementary Fig. 5. The results suggest that the lower limit of detection (LLOD) is highly lectin- and glycan-dependent. The LLOD for VVL binding to GalNAc was found to be around 0.0085 µg/ml (0.1 pmol), while LLOD for RCA-I, GSL-II, AAL, Con A, WGA and AAL was observed to be around 0.2 µg/ml. For the majority of glycans, the background MFI from the control beads was found to be negligible even when 25 µg/ml of lectin was used in the assays.

### Validation of MGBA using anti-glycan antibodies

We further evaluated the performance of MGBA using 13 anti-glycan antibodies (Supplementary Table 3). Four antibodies against proteins (two anti-human MUC1, one anti goat IgG and one anti rat IgG) were used as negative controls and did not bind to any glycan in our glycan array (Supplementary Fig. 6). A commercial Le^y^ antibody of the IgM class was found to bind specifically (*K*_d_ = 3.06 ± 0.61 µM, mean ± SD, Supplementary Fig. 7) to the Le^y^ glycan Fuc-α2-Gal-β4-(Fuc-α3-)GlcNAc (#60**)** and weakly to Fuc-α2-Gal-β4-(Fuc-α3)GlcNAc-β3-Gal (#134) but did not recognize any other structures in the array (Fig. 3b). A monoclonal IgG antibody produced in our own laboratory was known to bind to glycans but the binding specificity was unknown. Using MGBA, it was quickly revealed that this antibody binds with high specificity (*K*d = 0.92 ± 0.079 µM, mean ± SD, Supplementary Fig. 7) to glycan #60 which contains the Le^y^ motif (Fig. 3b), demonstrating utility of MGBA for mapping glycan-binding specificity of antibodies.

The blood group H (BGH) antibody had identical binding profile as the UEA lectin with the only exception of glycan #124. The BGH antibody binds to two BGH-type 2 motif-containing glycans (#55 and 60) and one BGH-type 5 glycan (#58) recognized by UEA as well as glycan #124 that is not recognized by UEA (Fig. 3b). Our data suggest that anti-BGH antibody used to test the MGBA recognizes (Fuc-α2)-Gal-β4-x motif. If the motif changes to BGH-type 1 as in glycan #123 [(Fuc-α2)-Gal-β3-*x*] or (Fucα1-3)-Gal β4-x as in glycan #32, binding is abolished (Fig. 3b). The monoclonal antibody raised against GA1 structure (Gal-β3-GalNAc-β4-Gal-β4-Glc) specifically binds to glycan #171 as expected. The Le^b^ antibody recognized the Le^b^ glycans (#61 and 137) but also bound to several glycans containing Gal-β4-Glc (#15**)** and Gal-β3-(Fuc-α4)-GlcNAc (#26, 62,135 and 136) structures. The Lac/Gal-β4-Glc-β (#15) structure was recognized by CA19.9 (IgG and IgM class), sLe^x^, and Le^b^ antibodies (Supplementary Fig. 6).

### Validation of MGBA by glycan-binding proteins

**Fig. 4:**
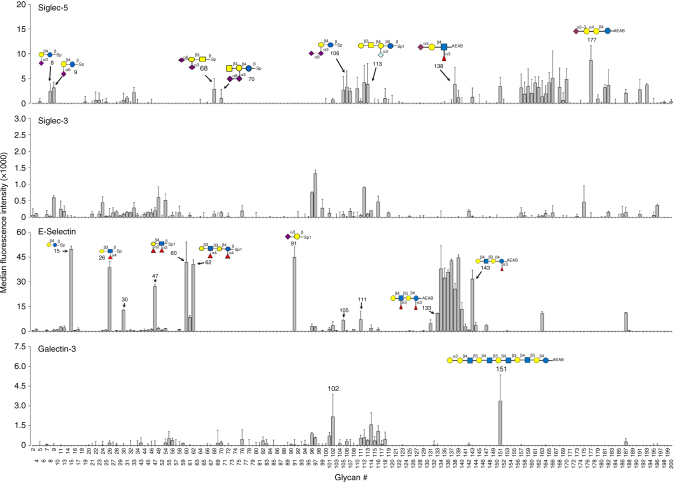
MGBA’s specificity for glycan-binding proteins. The data for four representative GBPs are shown here and the data for other GBPs are shown in Supplementary Fig. 7. Representative glycans binding to the GBPs are shown for siglec-5 (top), E-selectin (lower middle) and galectin-3 (bottom). Mean of two replicates is presented and error bars are standard deviations of the two replicates. The experiment was repeated three times

To assess the utility of MGBA, we analyzed representative glycan-binding proteins in different GBP families: mouse E-selectin (C-type lectin), siglec-5 and siglec-3 (siglecs) and galectin-3 (galectins). In addition to these GBPs, three proteins that are not expected to bind to glycans (transferrin, α-1 acid glycoprotein and carbonic anhydrase 9) were tested against a subset of 50 glycans. As expected, none of the proteins showed appreciable binding to any glycan (Supplementary Fig. 8), further confirming the high specificity of the MGBA platform. It is well known that GBPs have multivalency for binding glycans^35,36^. In our studies, siglec-5 recognized several sialic acid-containing glycans such as Neu5Ac-α3-Gal-β4-Glc motif (#8) and Neu5Ac-α6-Gal-β4-Glc (#9) as well as Gal-β3-GalNAc-β4-(Neu5Ac-α3-)Gal-β4-Glc (#112) and its structural analog Gal-β3-GalNAc-β4-(Neu5Gc-α3-)Gal -β4-Glc (#113). However, it did not bind to any of the glycan structures containing the Gal-β4-Glc motif (Fig. 4). Siglec-3 showed weak binding to glycan structures containing sialic acid. Similar to Siglec-3, the overall binding to glycans by galectin-3 was weak with the highest signal observed for glycan #151 [Gal-α3-(Gal-β4-GlcNAc-β3)_4_-Gal-β4-Glc] (Fig. 4). Low levels of signals were also observed to glycans containing the Sia-α3-Gal-β4-Glc (#111, 112, and 114), Sia-α3-Gal-β4-(6 S)GlcNAc (#116), NeuraGc-α3-Gal-β4-Glc motif (#113), GM2 KDN (#115) and the glycoside antibiotic sisomycin, (102) (Fig. 4). Mouse E-selectin showed high signal levels for glycans containing Lewis motifs (#60, 61, 62, 131, 133, 134, 135, 136, 137, 138, 139) and showed binding to lactodifucotetraoase (#140), lacto-*N*-neofucopentaose (#143), GT3 ganglioside (#163) and 2’2Difucosyl lactose (#187) as well as other glycan structures (#15, 26, 30, 47, 91, 105, and 111) (Fig. 4). Why the binding signals vary between different GBPs is not entirely clear. However, we do know that the amount of GBPs required to detect binding vary for different GBPs (Fig. 3a).

### Analyses of natural anti-glycan IgM antibodies in human sera

**Fig. 5:**
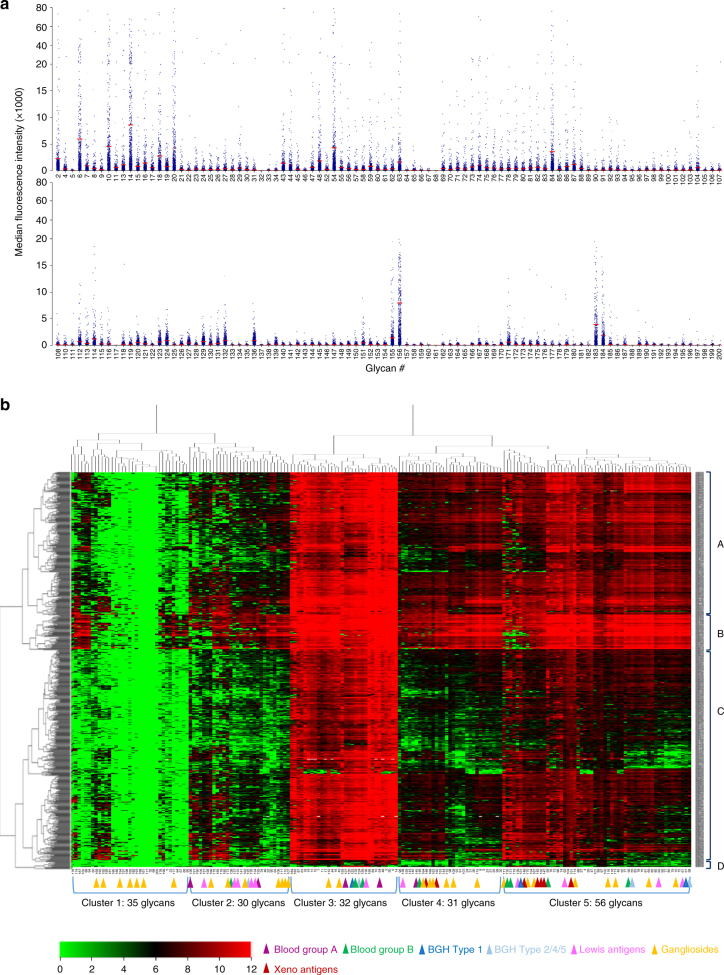
Natural anti-glycan IgM antibodies in 613 human serum samples measured on MGBA. **a** Dot plots of anti-IgM antibody levels (MFI) for each glycan in 613 individuals. Each dot represents one individual and mean for each glycan is represented by the red line. Background-subtracted net MFI data (MFI×1000) on *y*-axis is expanded for 0–20,000 range to improve visualization. **b** Heatmap of anti-IgM antibody levels in 613 samples. The data are Log 2 transformed. Each row represents an individual sample and each column represents an individual glycan. IgM levels are represented by the color codes as indicated by the color bar. Serum IgM was used to cluster glycans (Cluster 1–5) as well as subjects (Cluster **a**–**d**). Cluster A, B, C and D contain 221 (36%), 54 (8.8%), 325 (53.1%), and 13 (2.1%) subjects, respectively. The sample clusters are not associated with demographic or clinical variables such as collection date, gender and age of the subjects, and batch of sample analyses

MGBA was used to measure natural anti-glycan IgM antibodies in 613 human serum samples recruited in the Phenome and Genome of Diabetes Autoimmunity (PAGODA) study. We initially titrated serum concentration and here present data using 1:2500 dilution which yields low background and good signals for many glycans. As shown in Fig. 5a, the levels of anti-glycan antibodies vary greatly for different glycans, with net (background-subtracted) MFI below 1000 in most individuals for about 40% of the glycans and MFI in tens of thousands for most subjects for over 30 glycans (Fig. 5a). We determined positivity of a subject for each glycan if the observed net MFI is greater than the mean plus 3 times of standard deviation of the background. The percentage of subjects with positive IgM antibodies is presented in Supplementary Table 4. For 82 of the 184 glycans, including #58, 130, 113, 62, 2, 190, 189, 27, 72, 80, 85, 185, 144, 154, 92 and 70, over 50% of the subjects have positive IgM, while less than 10% of the subjects are positive for IgM for 44 glycans.

IgM levels in all 613 subjects were used to cluster the 184 glycans (Fig. 5b). The heatmap and clustering revealed five major clusters of glycans as defined by the serum IgM levels. The 35 glycans in Cluster 1 are either completely negative for IgM in all subjects or have only a few individuals with detectable antibodies, while a tightly clustered group of 32 glycans (Cluster 3) detected high levels (MFI >1000) of IgM antibodies in ~95% subjects. Cluster 4 glycans detected moderate levels of IgM in about 50% of the subjects, while Cluster 5 glycans detected high levels of IgM in most subjects.

In our MGBA, serum IgM antibodies were detected for blood groups A, B and H glycan antigens. Eight glycans on MGBA contain the blood group-B motif. Four of the glycans (#56, 59, 130 and 131) detected moderate levels of IgM in most subjects (Cluster 5) and two glycans (#129 and 132) detected high levels of serum IgM antibodies in 90% of the subjects (Clusters 3). The seventh glycan (#146), which contains the blood group-B motif but has a complex structure (two fucose), had moderate reactivity for IgM in about 50% of the subjects (Cluster 4). The MGBA also contains five glycans with the blood group-A motif. Two of these glycans, #54 [GalNAc-α-1,3-(Fuc-α-1,2)-Gal-β] and #127 [GalNAcα1-3(Fucα1-2)Galβ1-3GlcNAcβ1-3Galβ1-4Glc, detected high IgM levels in almost all subjects (Cluster 3). Two glycans, #125 [GalNAcα1-3(Fucα1-2) Galβ1-3GalNAcβ1-3Gal] and #126 [GalNAcα1-3(Fucα1-2) Galβ1-4GlcNAcβ1-3Galβ1-4Glc] had very low reactivity (Cluster 2), while #145 [GalNAcα1-3(Fucα1-2) Galβ1-4(Fucα1-3)-Glc] detected moderate levels of IgM in about 50% of the subjects (Cluster 4). The IgM data and the glycan structure relationships argue that the linkage and residue on the reducing end of the motif also influence the binding to serum IgM antibodies.

The reactivity to BGH glycans was spread into three clusters. The BGH type 1 glycan (#123) and one BGH type 2 glycan (#124) detected high levels of serum IgM in ~90% of the subjects (Cluster 3), while one BGH type 2 glycans (#55) and one BGH type 5 (#58) detected moderate levels of IgM in most subjects (Cluster 5). The BGH type 4 glycan (#122) was found to have low reactivity (Cluster 2). Our glycan array featured ten Lewis antigen glycans. Three of them are in Cluster 5 (#60–62, 139), one in Cluster 3 (#136) and one in cluster 4 (#138), and the remainder in Cluster 2.

Low reactivity was found for asialo GM2/GA2 (#173), GD1a (#169), GT2 (#166), GM3 (#161), GD3 (#106, 107 and 162), GT3 (#110 and 111) and 3-sialyl Gb3 (#177) (all in Cluster 1 and 2). Nine gangliosides, including fucosylated GM1 (#172), asialo GM1/GA1 (#171), GM2 (#164 and 69), GM1a (#113 and 167), GD2 and GD2-Gc (#70 and 114), GD1b (#170), GT3 (#163), and GM1b (#168) are all grouped in Clusters 4 and 5. Only sialylated GM1 (#112) and asialo GM2 (#43) are clustered in Cluster 3.

The IgM data on all 184 glycans were used to cluster the subjects and four main clusters of subjects are recognizable (Fig. 5b). The subjects in cluster A showed strong IgM reactivity to glycans of cluster 3, 4 and 5. Cluster B subjects had high levels of antibodies against glycans in Clusters 3, 4, and 5 as well as some glycans in Cluster 1 and 2. Cluster C subjects are positive for all glycans in Clusters 3 and most Cluster 5 glycans but mostly negative for Cluster 1, 2 and 4 glycans. Cluster D includes a small number of subjects (*n* = 13, 2.1%) who are negative for almost all glycans. We determined that these subject clusters are not related the subject gender, subject age at sampling, year of sampling or the plates for sample analyses (Supplementary Fig. 9).

### Anti-glycan IgG as prognostic biomarkers for ovarian cancer

**Fig. 6:**
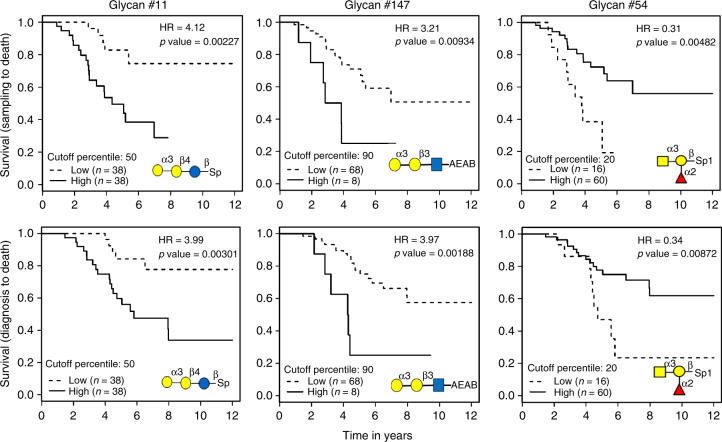
Kaplan–Meier survival analyses of ovarian cancer patients. Analysis was done using date of sample collection to death (top row) and date of diagnosis to death (bottom row). The subjects were assigned to the low- (dashed lines) or high- (solid lines) level groups based on the anti-glycan IgG levels for each glycan. The data for three glycans are shown here. The hazards ratio (HR) was determined by Cox regression analysis and the *p* values were determined using log-rank test

A total of 348 serum samples from 119 ovarian cancer patients were analyzed for the levels of anti-glycan IgG using MGBA. All patients underwent primary debulking surgery followed by six cycles of platinum plus taxol chemotherapy. Serum samples were obtained before surgery and at different time points after chemotherapy. Of these patients, 76 achieved complete response (or remission) after six cycles of chemotherapy and serum samples at remission were available. As shown in Supplementary Fig. 10, the anti-glycan IgG levels vary from glycan to glycan, with glycan #54 and #149 having the highest IgG levels in these patients. Overall, serum anti-glycan IgG titers are much lower than anti-glycan IgM across almost all glycans on this MGBA. To illustrate the potential application of MGBA, we focused our analyses on the potential of using these antibody levels as prognostic biomarkers for ovarian cancer. Similar to our previous analyses on serum proteins^37^, anti-glycan IgG was determined for the at remission serum samples from 76 patients and Kaplan–Meier survival analyses were carried out using two phenotypes: survival from the date of sample collection to death and from the date of diagnosis to death (Fig. 6). IgG against glycan #11 (Gal-α3-Gal-β4-Glc) is the best prognostic biomarker in this data set. Patients with higher IgG levels against glycan #11 have significantly lower probability of survival than patients with lower IgG. This is true for both analyses using the survival data from sampling date to death (Cox Regression Hazards Ratio (HR) = 4.12, Log-rank *p* = 0.00227) and from diagnosis to death (HR = 3.99, Log-Rank *p* = 0.00301). Glycan #11 is an abundant trisaccharide (known as Pk antigen), a key fragment of glycosphingolipid globotriaosylceramide (Gb3Cer) which is a component of erythrocyte and endothelial cell membrane. Antibodies against this trisaccharide were found to bind to synthetic version of the glycan as observed here but not to glycan on cell membrane^38^. The binding difference between synthetic and natural forms of the trisaccharide is probably due to the different spacing and conformation^38^. Our results suggest that ovarian cancer cells may express the trisaccharide in a conformation that are different from those on normal cells and can induce an antibody response in ovarian cancer patients. High levels of anti-glycan antibodies for a similar trisaccharide (#147: Gal-α3-Gal-β3-GlcNAc) are also associated with poorer survival (HR = 3.97, Log-Rank *p* = 0.00188). On the other hand, higher levels of IgG antibodies against blood group-A trisaccharide (#54), are associated with better survival (HR = 0.31, Log-Rank *p* = 0.0048) (Fig. 6).

## Discussion

The ability to probe the interaction between glycans and GBPs is of critical importance to biomedical research and clinical care of patients. However, progress in this field has been hampered by the difficulty and availability of suitable technologies. Glycan arrays printed on nitro-cellulose membrane, ELISA plates, or glass slides^39,40^ have been developed and used in various settings. Each of these technologies have limitations, including throughput, multiplex capability, sensitivity, specificity, upfront costs of the setup, assay cost and technical difficulty. Glycan arrays printed on nitro-cellulose membrane have limited number of glycans (usually a few to a few dozens) and the sample throughput is also very low. Glycan arrays on ELISA plates usually contain only one glycan per well. Although the sample throughput can be high if only a few glycans are to be analyzed, the throughput becomes low for large number of glycans. Furthermore, the dynamic range of signals for ELISA based on optical density is much lower than fluorescent detection as done with MGBA. On the other hand, glycan arrays on glass slides can contain hundreds or more glycans per slide, while only a few samples can be processed by a single technician and the procedure for glass arrays is longer than MGBA. Furthermore, the setup for producing glass arrays and performing the assay costs several hundred thousand dollars and can be prohibitive and highly trained personnel is required for both printing and assay. Therefore, the glass array platform has never been used in a clinical setting. Another key advantage of MGBA over glass arrays is its flexibility to rearrange the content of the array, a task that cannot be done easily for glass arrays.

The Luminex platform is based on 5.6 µm fluorescent microspheres, each distinguished by a different mixture of red and orange dye. The microspheres are excited with a red laser, and the resultant emission wavelengths are used to distinguish 500 unique spectrally encoded regions^41,42^. The high throughput, high-content technology has been widely used to analyze plasma proteomics^43^, autoimmunity^44^, kinase inhibitor^45^, protein phosphorylation^46^, miRNA^47^ and gene expression^48^. One research group has previously attempted to use the Luminex beads for glycan studies^24–26^. However, these studies used a cumbersome conjugation procedure and only a few glycans and a small number of serum samples were used in their studies, which do not allow a rigorous evaluation of the specificity, sensitivity and reproducibility of their assays. Here we developed a simple, efficient and reproducible one-step glycan-conjugation procedure and present our first version of the Luminex Multiplex Glycan Bead Array (MGBA) that can be used to simultaneously analyze the interaction between hundreds of glycans and various types of GBPs, including naturally occurring anti-glycan antibodies. Using a variety of glycan-binding proteins, including 39 plant lectins, 13 recombinant anti-glycan antibodies, 4 mammalian GBPs and close to 1000 serum samples, we demonstrated that MGBA is both high content and high throughput, and therefore are suitable for the analyses of large numbers of biological samples and glycans. The specificity of the binding on MGBA was validated using three different types of proteins that can bind to glycans: plant lectins, anti-glycan antibodies and mammalian glycan-binding proteins (selectins and galectins). For the vast majority of the plant lectins, the binding characteristics are largely consistent with the CFG data and previous knowledge. For example, RCA-I was found to recognize terminal Gal-β4-GlcNAc residues^33^ and the UEA showed specific binding to BGH type-2 structures but not the BGH type I structures^34^. The binding profiles of the anti-glycan antibodies are also consistent with their known specificity. We also demonstrated the utility of the MGBA to quickly identify the glycan specificity of new anti-glycan antibodies with unknown specificity.

There is a considerable interest in exploring the potential of using natural anti-glycan antibodies as potential candidate biomarkers for human diseases. Several studies suggested that serum antibodies to glycans may serve as biomarkers for autoimmune diseases such as inflammatory bowel disease and Crohn’s disease^49–51^. It was shown that serum antibodies to blood group-A glycans predict the survival of prostate cancer patients in a clinical trial of a poxvirus-based cancer vaccine^9,52^. It has also been shown that serum antibodies to glycans can distinguish ovarian cancer patients from controls with sensitivity and specificity comparable to the clinically used CA-125 test^22^. In this study, we illustrated the utility of using MGBA to identify potential candidate biomarkers for cancer by discovering novel anti-glycan antibodies that predict the survival of ovarian cancer patients. The MGBA platform is particularly suited for such population-based studies that require the analyses of large number of samples and for a large number of glycans. This platform can be applied to the studies of any human disease. However, like every other platform, the analytical range for MGBA is sample and glycan dependent. For screening purposes, this is not a major concern because one only wants to identify glycans of interest and precise values for samples with saturation signal are not critical at this stage and can be determined by sample dilutions later if desired. As a clinical assay, there are multiple ways to deal with the dynamic range issue. First, samples with saturation levels of signals are usually analyzed with multiple sample concentrations. Second, glycans can be organized into subpanels based on their signal levels and analyzed with sample dilutions in the linear range.

In summary, our data demonstrated that MGBA could produce specific binding data with various types of GBPs, although the assay conditions may need some adjustment for different types of GBPs. The MGBA assay is also sensitive for most GBPs and glycans. The results are very reproducible. The manufacturing of the glycan-conjugated beads is a routine chemical reaction that can be adopted in almost all research and medical laboratories with basic training in biochemistry and immune assays. The assay uses inexpensive equipment that is certified for clinical use, requires small sample volume and has a relatively fast turn-around time, 4 h for lectin/anti-glycan antibodies assays and for measurement of anti-glycan antibodies in serum. Therefore, it can be easily adopted by medical laboratories for clinical services. Because of these advantageous attributes of MGBA, we believe that it will become a method of choice to investigate glycan-GBP interactions and significantly advance glycobiology.

## Methods

### Synthesis of glycans

Glycans were synthesized using Core Synthesis and Enzymatic Extension approaches^31^. A 3 or 4 carbon spacer was attached to the reducing end as published by Bohorov et al.^53^. Bifunctional fluorescent tag, 2-amino(N-aminoethyl) benzamide (AEAB), and labelled glycans with a free amino group were also included in this study^32^. The chemical names and structures of 184 glycans used in this study are presented in Supplementary Table 1.

### Blood collection from human subjects

Serum samples used in the study were obtained from participants of the Phenome and Genome of Diabetes Autoimmunity (PAGODA), and Biomarkers and Therapy (BAT) Cancer Study. The institutional review board at Augusta University approved the study. Blood samples were collected in a serum separator tubes from BD Biosciences. The blood was allowed to clot for 30 min at room temperature and then centrifuged at 2500 × *g* for 10 mins. Serum was aliquoted and stored at −80 °C until use.

### Conjugation of glycans to Luminex beads

Glycans that contain a free amino group were chemically coupled to carboxylated beads with a unique fluorescent spectral address (Luminex Corp., Austin, TX, USA). The bead region is encoded by precise ratio of red and infrared fluorescent dyes, which can be identified by the Flex-MAP3D (FM3D) analyzer. Briefly, the stock vial of beads (1.25 × 10^7^ beads/ml) was vigorously vortexed for 30 s, and 100 µl (1.25 × 10^6^ beads) was aliquoted into a 1.5 ml polypropylene tube. The beads were separated by centrifugation at 10,000 × *g* for 5 min at room temperature (RT). The storage solution was discarded and beads were washed once with deionized water. Beads were then resuspended in 80 µl of deionized water, to which 10 µg of glycan and 2.5 µl of 1-ethyl-3-(3-dimethylaminopropyl) carbodiimide hydrochloride (EDC) solution (10 mg/ml in deionized water) was added. The beads were incubated on a shaker at RT for 2 h. Beads were then centrifuged (10000 g for 5 min) and resuspended in 50 mM Tris-buffered saline to block the reactive sites for 30 min. Beads were recovered and resuspended in 1 ml of 1% bovine serum albumin (BSA, w/v) in phosphate-buffered saline (PBS) overnight at RT. Following overnight blocking, beads were suspended in PBS containing 1% BSA (w/v) and 0.2% sodium azide (w/v), and stored in the dark at 4 °C. To assess the assay background, beads from three different bead regions were included as “no glycan conjugation” (NGC) controls. These NGC control beads underwent the same conjugation process at the same time as the glycan-conjugated beads.

### Plant lectin-binding assay

Thirty-nine plant lectins were obtained from Vector Laboratories (Burlingame CA, Supplementary Table 2). All lectins were diluted to 5 µg/ml with 1% BSA in PBS freshly prepared on the day of the assay. Before the assay, individual wells of a 384-well filter plate (EMD Millipore,MS, USA) were washed with 90 µl wash buffer (PBS containing 0.05% Tween-20). An individual microsphere stock suspension was mixed together to create an array and diluted with 1% BSA in PBS (final dilution 1:100). Ten microliters of the diluted microsphere suspension were added to each well, followed by addition of 10 µl of lectin solutions. The mixture was incubated at RT on a shaker set at 550 r.p.m. (IKA MTS4, Wilmington, NC) for 1 h. The reaction mixture was removed by vacuum suction and the wells were washed twice with wash buffer. After washing, 10 µl of streptavidin R-phycoerythrin (SAPE; 3 µg/ml freshly diluted in wash buffer) was added. After 30 min incubation with SAPE, all liquid from the wells were removed with vacuum suction. The beads were resuspended in 60 µl of wash buffer and the median fluorescence intensities (MFI) were measured on a FlexMAP 3D reader (Millipore, Billerica, MA) with the following instrument settings: events/bead: 50, minimum events: 0, flow rate: 60 µl/min, sample size: 50 µl and doublet discriminator gate: 8000–13,500.

### Assay for recombinant anti-glycan antibodies

Thirteen anti-glycan antibodies were obtained from commercial sources (ABCAM, US biologicals and EMD Millipore) or produced in-house (Supplementary Table 3). Individual bead stocks were diluted 1:100 in PBS containing 1% BSA, and 10 µl of diluted beads were added to prewashed wells of a 384-well filter plates. Ten microliters of diluted antibodies (2 µg/ml) were added and the mixture was incubated at RT for 1 h. The reaction mixture was removed by vacuum suction and the wells were washed two times with wash buffer. After washing, PE-labelled anti-mouse IgM or anti-mouse IgG (2.5 µg/ml, Southern Biotech, AL, USA) were added to the wells and incubated for one additional hour. The plates were washed two times with wash buffer and the beads were resuspended in 60 µl of wash buffer and the MFI was measured on FM3D reader with settings described above.

### Affinity determination of binding on MGBA

To investigate avidity of binding to glycans on MGBA, competitive assays were done according procedures described by Rath et al.^54^. Briefly, Le^y^ (gly#60)-conjugated beads were incubated with Le^y^ IgM antibody and the Le^y^ IgG antibody (0.5 µg/ml in 1%BSA/PBS) for 1 h. The beads were then washed two times to remove the unbound antibodies. Diluted Le^y^ was then added to the well at decreasing concentrations (25–0.000423 µM), and incubated for 1 h at 37 °C. At the end of the incubation, the plate was washed and bound antibody was detected with 3 µg/ml of SAPE. The binding affinity (Kd) was defined as the concentration of free LeY that blocked 50% of the antibody binding to the immobilized Le^y^ beads.

### Recombinant glycan-binding protein assay

Recombinant FC chimera containing siglec-3, siglec-5, E-selectin and galectin-3 were obtained from Biolegend (San Diego, CA). The GBPs were processed as described by Blixt et al^35^. Briefly, a 25 µg/ml of the GBP solution was mixed with secondary and PE-labelled tertiary antibody in a ratio of 1:0.5:0.25 and incubated at 37 °C for 30 min. After formation of the multivalent GBP-antibody complexes, a 10 µl aliquot was added to the microsphere mixture in a well of 384 well plate, and further incubated for 1 h with shaking on a shaker set at 550 r.p.m. (IKA MTS4, Wilmington, NC). The microsphere-GBP complexes were washed with 25 mM Tris-HCl buffer pH 7.5, containing 75 mM of NaCl, three times and resuspended in the same buffer. The beads-GBP complexes were read using a Luminex FM3D machine as described above.

### Profiling of natural anti-glycan IgM in human serum

Human serum samples were diluted 2500-fold in 5% BSA in 25 mM Tris-HCl, pH 7.5 containing 75 mM NaCl and 0.8% polyvinyl pyrrilidone 360 (PVP). Briefly, 1000 microspheres for each glycan were added to each well and incubated with 10 µl of diluted serum sample for 2 h. After 2 h, unbound reagents were removed by vacuum suction and the wells were washed with wash buffer (25 mM Tris-HCl, pH 7.5 containing 75 mM NaCl and 0.025% Tween-20). Detection was performed by adding biotinylated anti-human IgM (Southern Biotech, AL, USA) to each well and the plates were incubated for 1 h. After incubation, the plates were washed and incubated with SAPE (3 µg/ml in wash buffer) for 30 min. The plates were washed and the beads resuspended in 60 µl of wash buffer. The MFI was captured using Luminex FM3D machine.

### Measuring natural anti-glycan IgG antibodies in human serum

Glycans were conjugated using one-step EDC method, the unreactive sites on the beads were blocked with 1% human serum albumin prepared in phosphate buffer saline. For measurement of anti-glycan IgG, human serum samples were diluted 500-fold in 1% BSA in PBS. Briefly, 1000 microspheres for each glycan were added to each well and incubated with 10 µl of diluted serum sample for 2 h. After 2 h, unbound reagents were removed by vacuum suction and the wells were washed with wash buffer (PBS containing 0.1% Tween-20). Detection was performed by adding biotinylated anti-human IgG (3 µg/ml in 1% HSA/PBS Southern Biotech, AL, USA) to each well and the plates were incubated for 1 h. After the incubation, the plates were washed and incubated with SAPE (3 µg/ml in wash buffer) for 30 min. The plates were washed and the beads resuspended in 60 µl of wash buffer. The MFI was captured using Luminex FM3D machine.

### Data analysis

Raw data files from Luminex FM3D machine were processed using a software pipeline, which we have developed for quality control, visualization and normalization of raw Luminex data. Wells with individual bead counts below 30 were flagged for exclusion and were not included in further analyses. The average of three no glycan control (NGC) beads was compared with the mean MFI value for each glycan without sample (blank control). The higher value of NGC and blank control was used for background subtraction. Bar plots represent mean MFI with standard deviation values as error bars from replicates. Dot-plots were generated to summarize the distribution of MFI levels for each glycan on log2 scale, each sample for each glycan is represented using a dot. The binding levels of the natural antibodies in human serum were described using four categories: no binding: MFI <3×SD of the no glycan control (NGC), weak/low binding: mean MFI of NGC+3×SD, elevated/moderate: MFI >5 × mean MFI of NGC and strong/high: MFI >20 × mean MFI of NGC. Visualization of MFI matrix was performed using heatmap, after log2 transformation, to graphically summarize the global interaction between glycans and glycan-binding proteins. The red color was used to represent larger values and green color was used to represent smaller values. Hierarchical clustering was performed to discover the patterns and to arrange the profiles based on the glycan-GBP interactions. To assess the prognostic potential, we used Cox regression analysis and Log-Rank test to evaluate the impact of serum anti-glycan IgG levels on survival. Overall survival was calculated as time from diagnosis date to the death of patient and date of the analyzed sample to the death of patient. Patients who are alive with no evidence of disease were censored at the date of last follow-up visit. Kaplan–Meier plots are shown and statistical significance was assessed using the log-rank test. Antibody affinity (IC_50_) was determined using “drc” package in R. All analyses were performed using the R language and environment for statistical computing (R version 2.15.1; R Foundation for Statistical Computing; www.r-project.org).

### Data availability

All the relevant data are available from the authors upon request.

## Electronic supplementary material

Supplementary Figures and tables

Descriptions of Additional Supplementary Files

Dataset 1
