# Supplementary material for: Multiplex glycan bead array for high throughput and high content analyses of glycan binding proteins
Source: Nat Commun. 2018 Jan 17;9:258. doi: 10.1038/s41467-017-02747-y (PMC5772357; doi:10.1038/s41467-017-02747-y)
Supplement: Supplementary file 1 — Supplementary Figures and tables [file 41467_2017_2747_MOESM1_ESM.pdf]

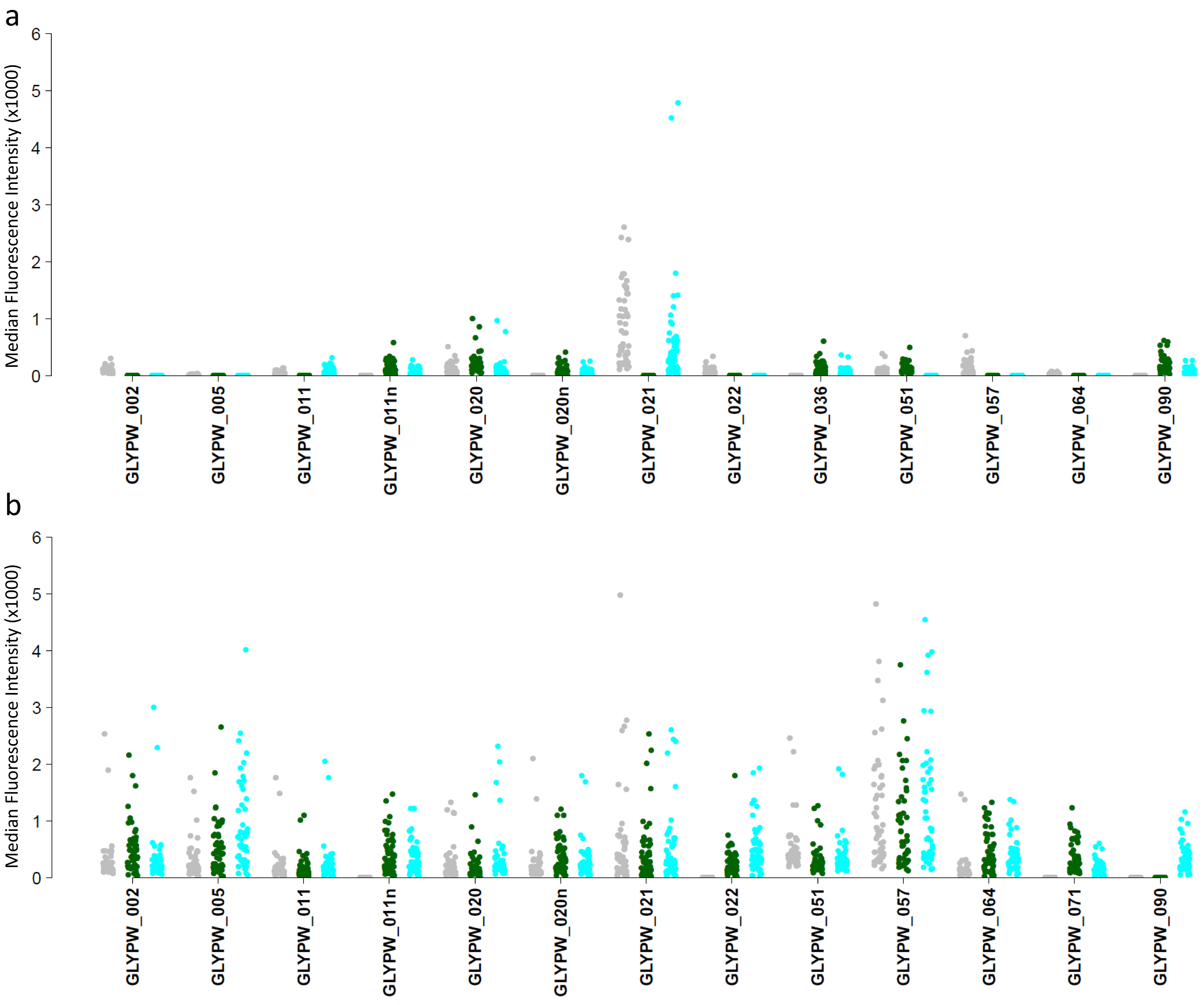

Supplementary Figure 1: Comparison of three conjugation methods. a: conjugation of glycans to Luminex beads using DMTMM, b: conjugation of glycans to beads using single-step EDC conjugation and c: two step conjugation by activating the beads with Sulfo-NHS and EDC prior to addition of glycans. All conjugations were repeated on three different days to assess the reproducibility of the conjugation strategies. We measured anti-glycan IgM in serum samples (n=48) to compare the conjugation strategies. Conjugations were performed on three different days day1 (grey), day 2 (dark green) and day 5 (cyan).

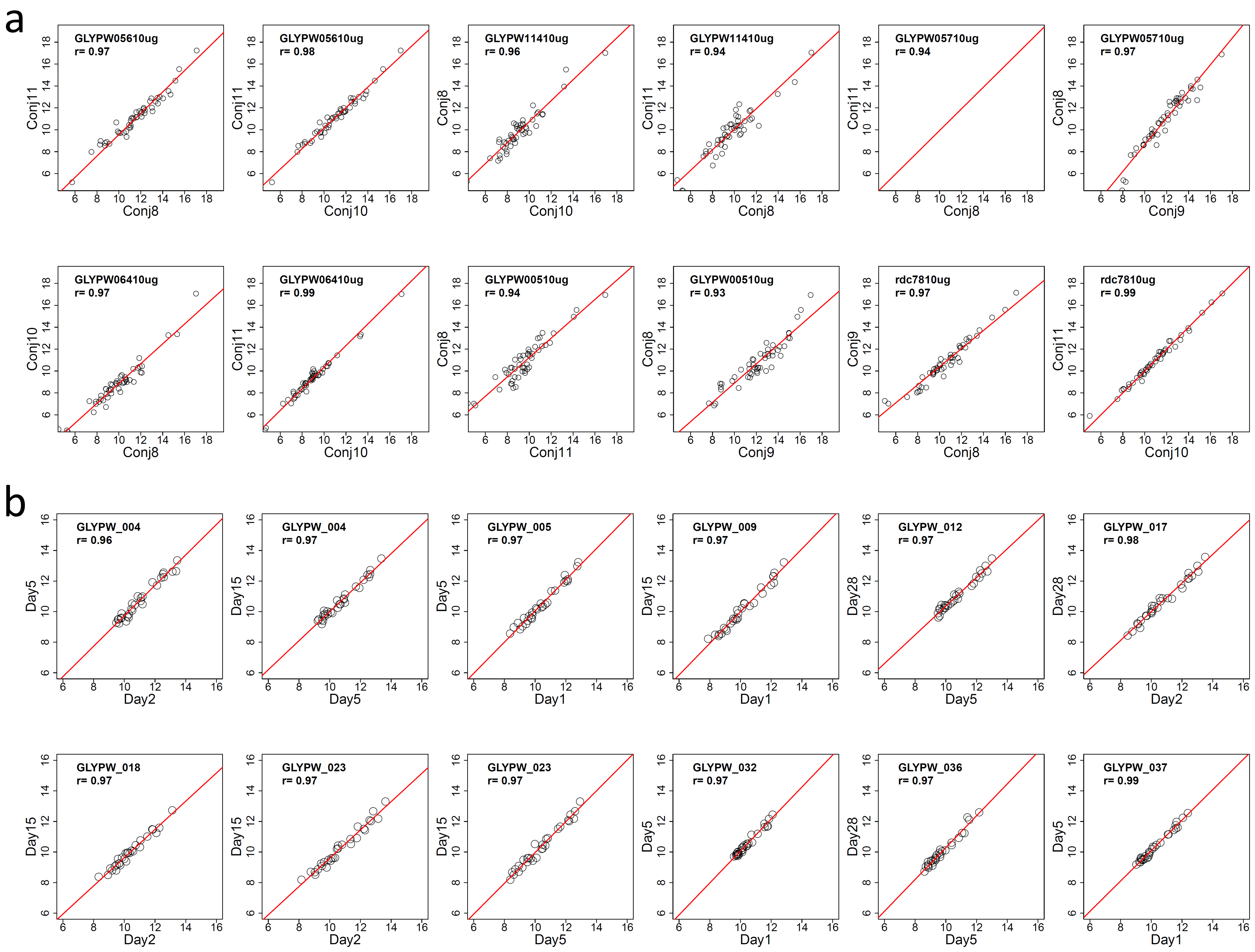

Supplementary Figure 2: Reproducibility of conjugation and profiling process for the MGBA. **a**) Reproducibility of the conjugation process. Selected glycans were conjugated to beads using EDC conjugation on Days 1, 2, 5, 15, 20. Reproducibility was assessed by measuring anti-glycan IgM in serum samples of 48 individuals in the same plate. Log2 transformed median fluorescence intensities (MFI) were compared using linear regression and correlation coefficient (R) was calculated. **b**) Reproducibility for the assay at different assay times. The same batch of bead conjugation was used to measure anti-glycan IgM in 48 serum samples on five different days (1, 2, 5, 10 and 28). Linear regression and coefficient of correlation (R) between the log2 transformed MFI values for different days were used to measure reproducibility.

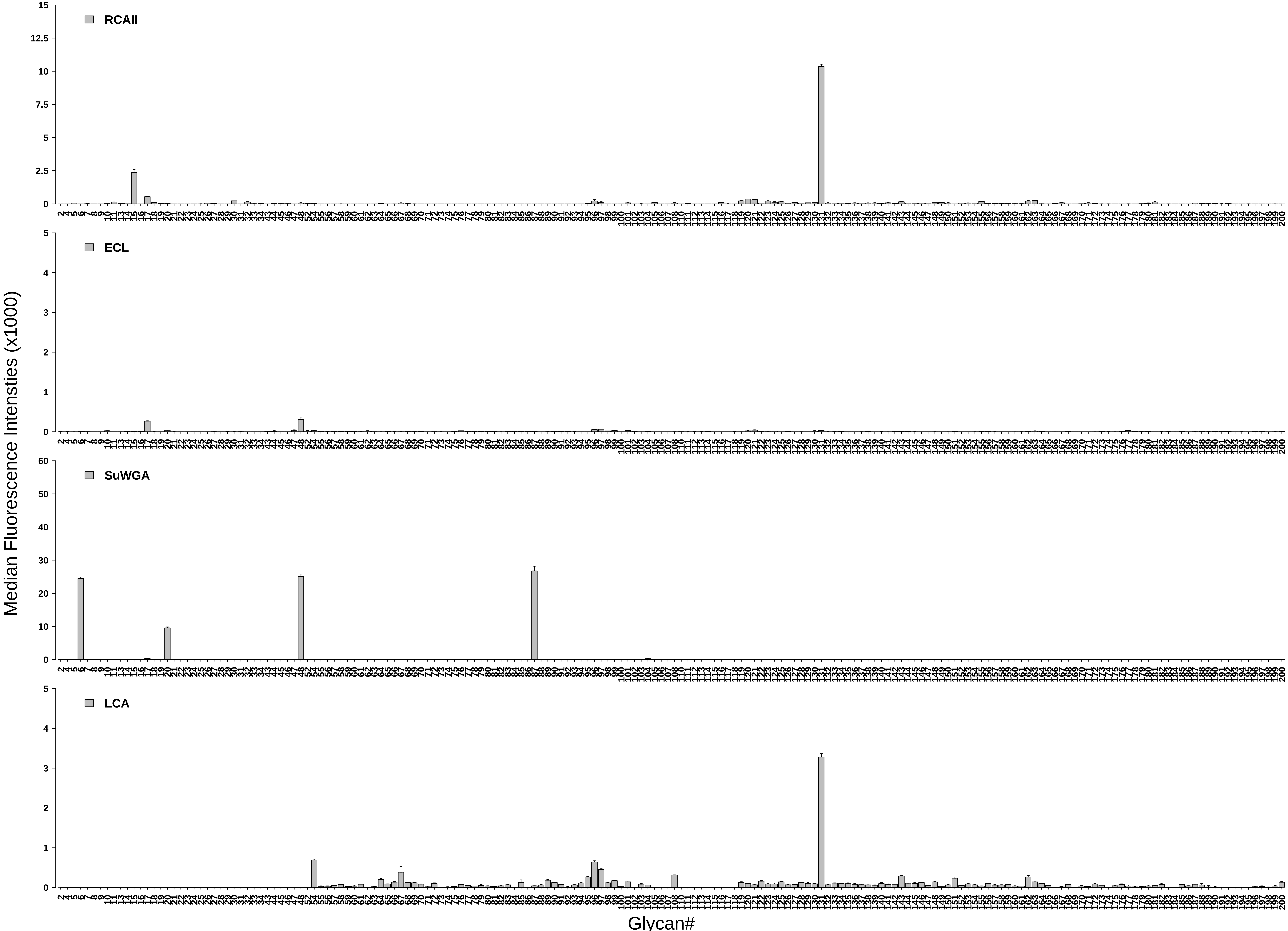

RCAII
ECL
SuWGA
LCA
Median Fluorescence Intensties (x1000)
Glycan#

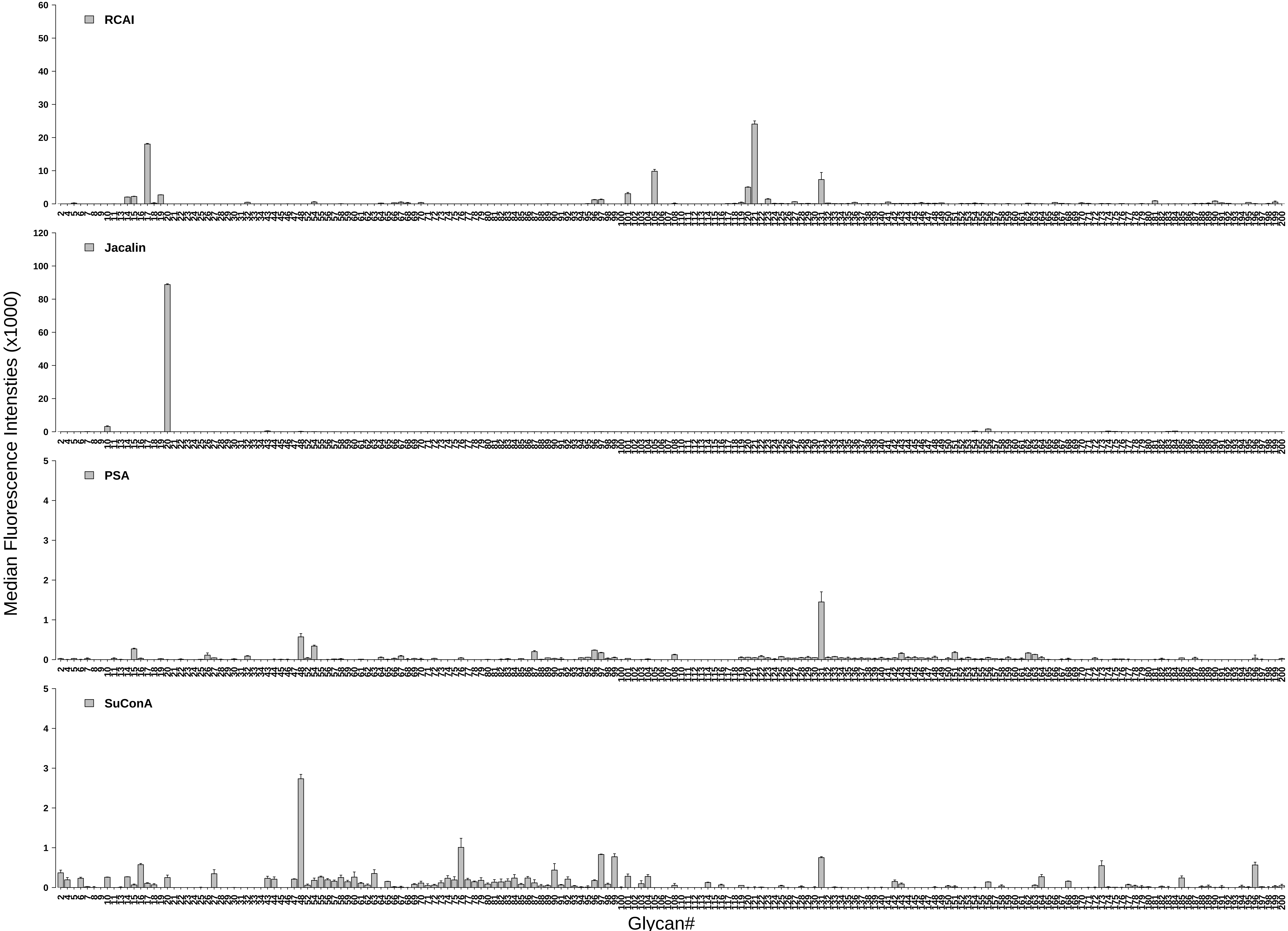
RCAI
Jacalin
PSA
SuConA
Median Fluorescence Intensties (x1000)
Glycan#

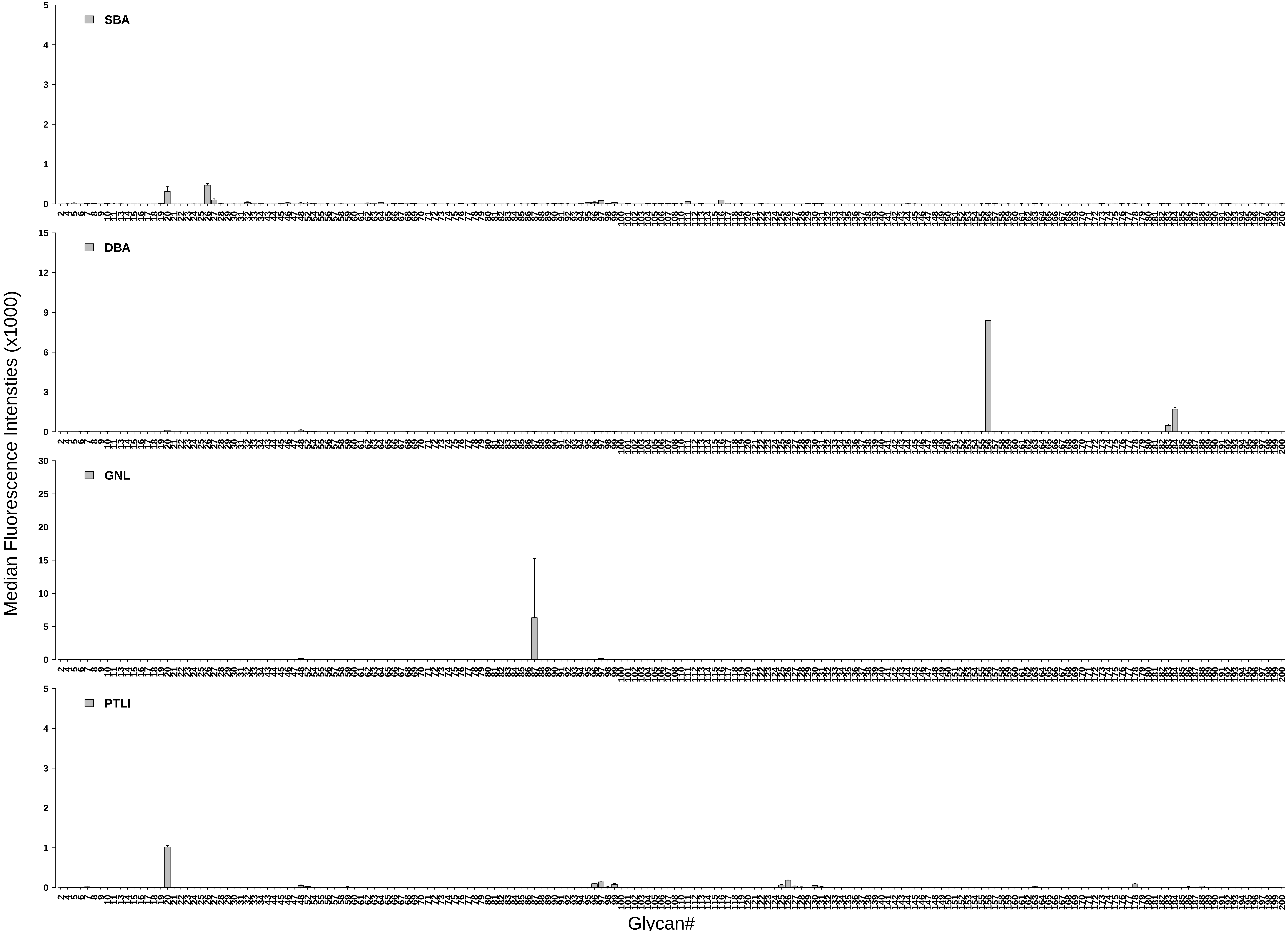
SBA
DBA
GNL
PTLI
Median Fluorescence Intensties (x1000)
Glycan#

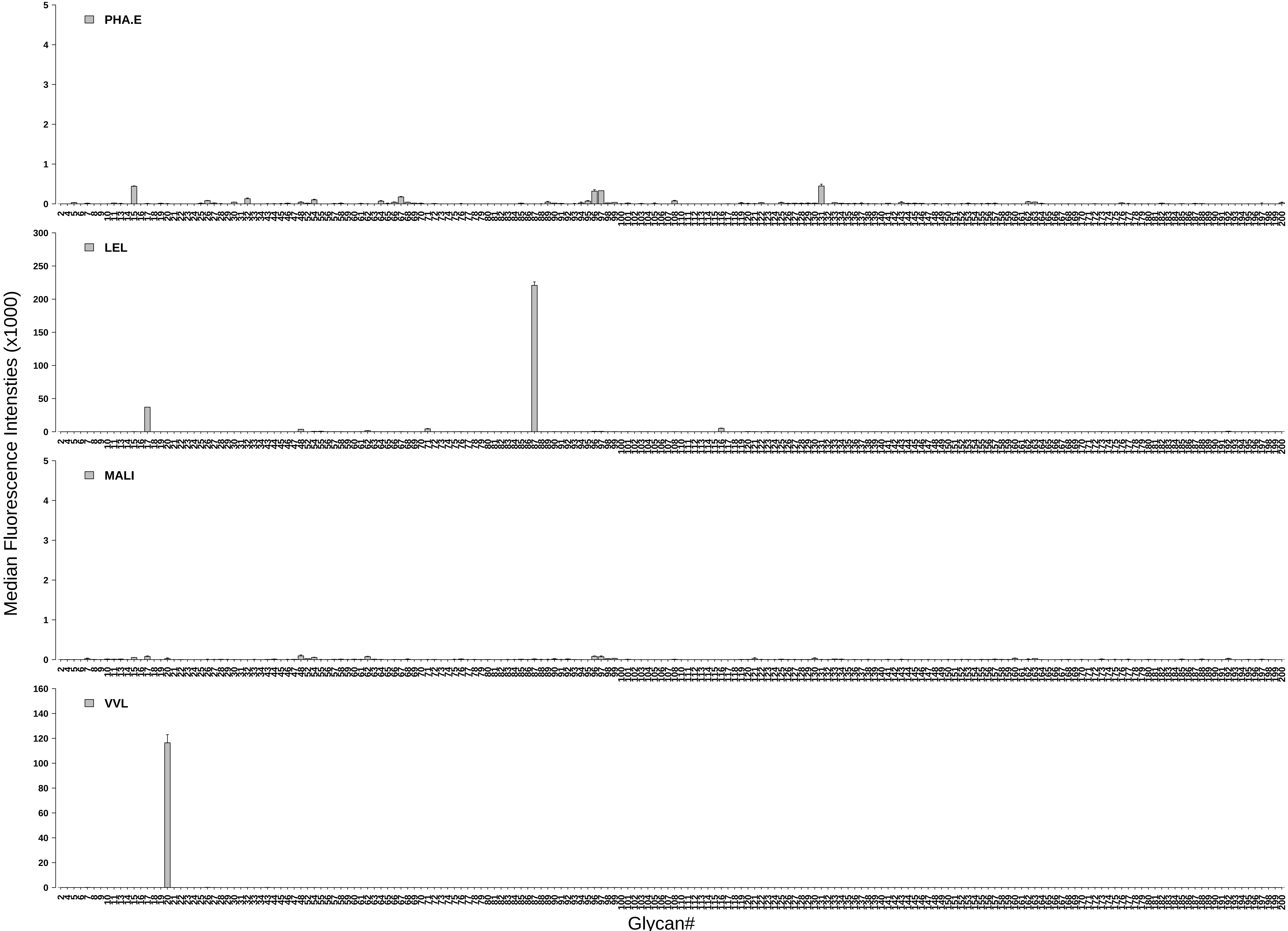
PHA.E
LEL
MALI
VVL
Median Fluorescence Intensties (x1000)
Glycan#

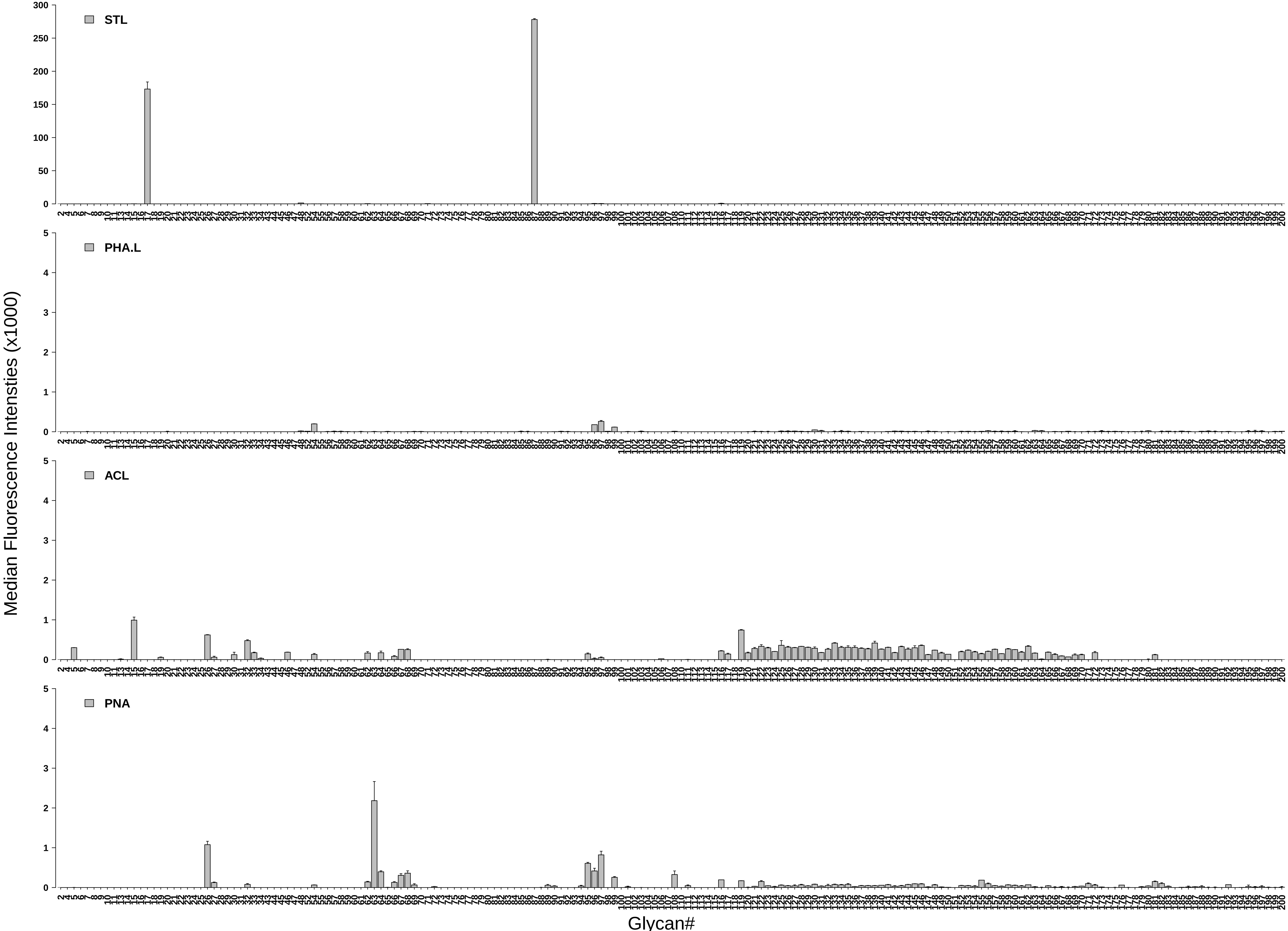

STL
PHA.L
ACL
PNA
Median Fluorescence Intensties (x1000)
Glycan#

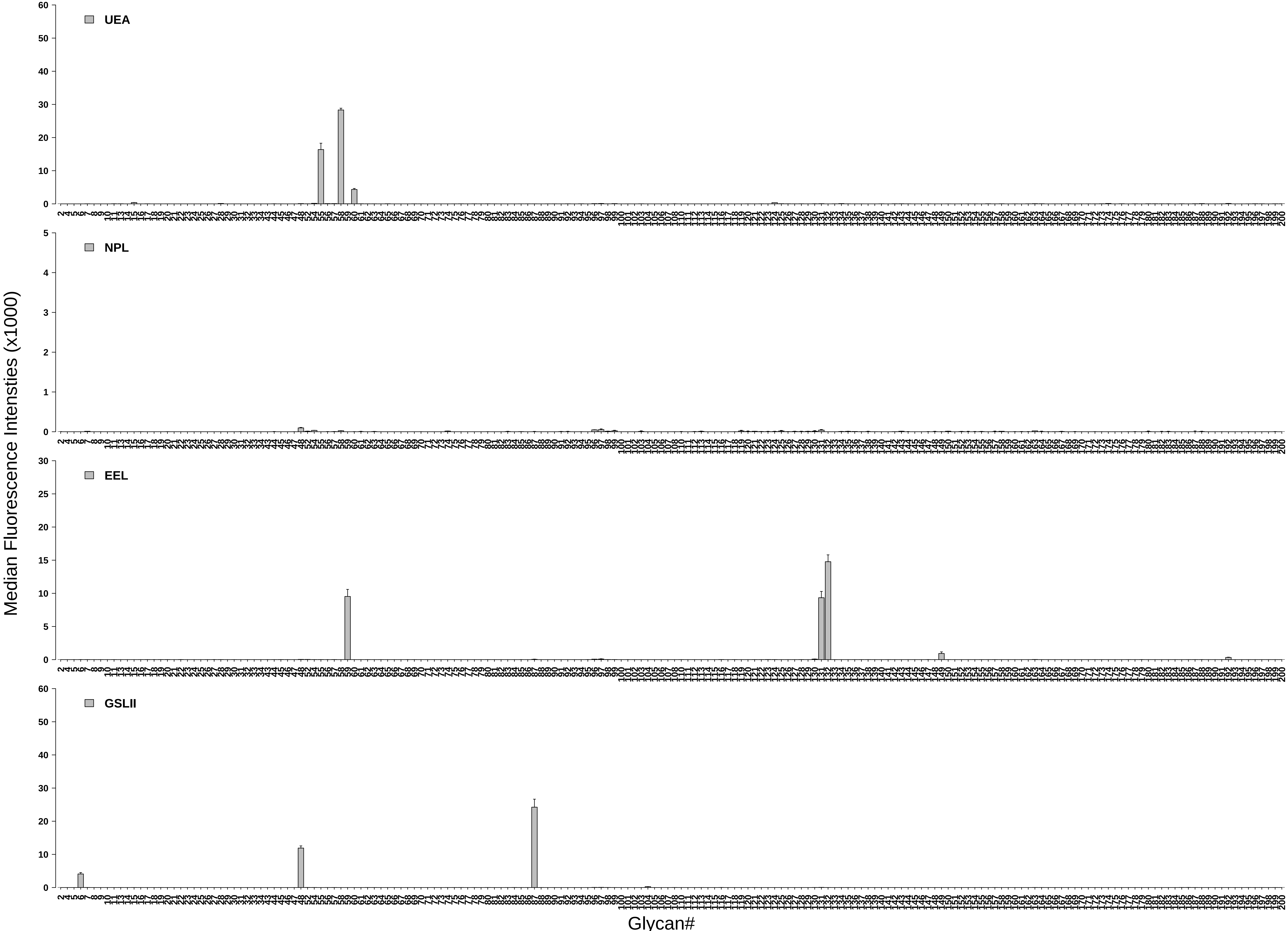
UEA
NPL
EEL
GSLII
Median Fluorescence Intensties (x1000)
Glycan#

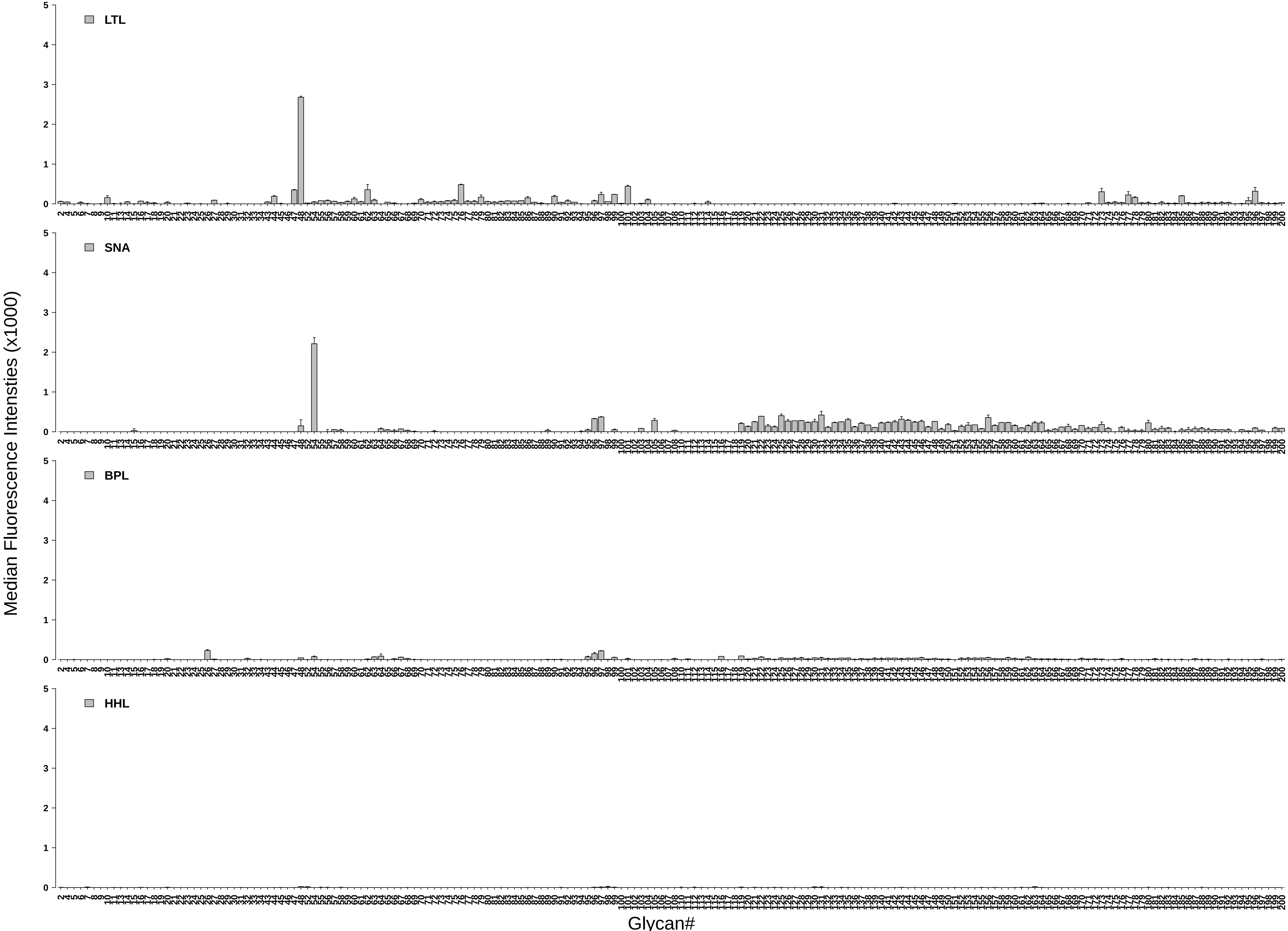

LTL
SNA
BPL
HHL
Median Fluorescence Intensties (x1000)
Glycan#

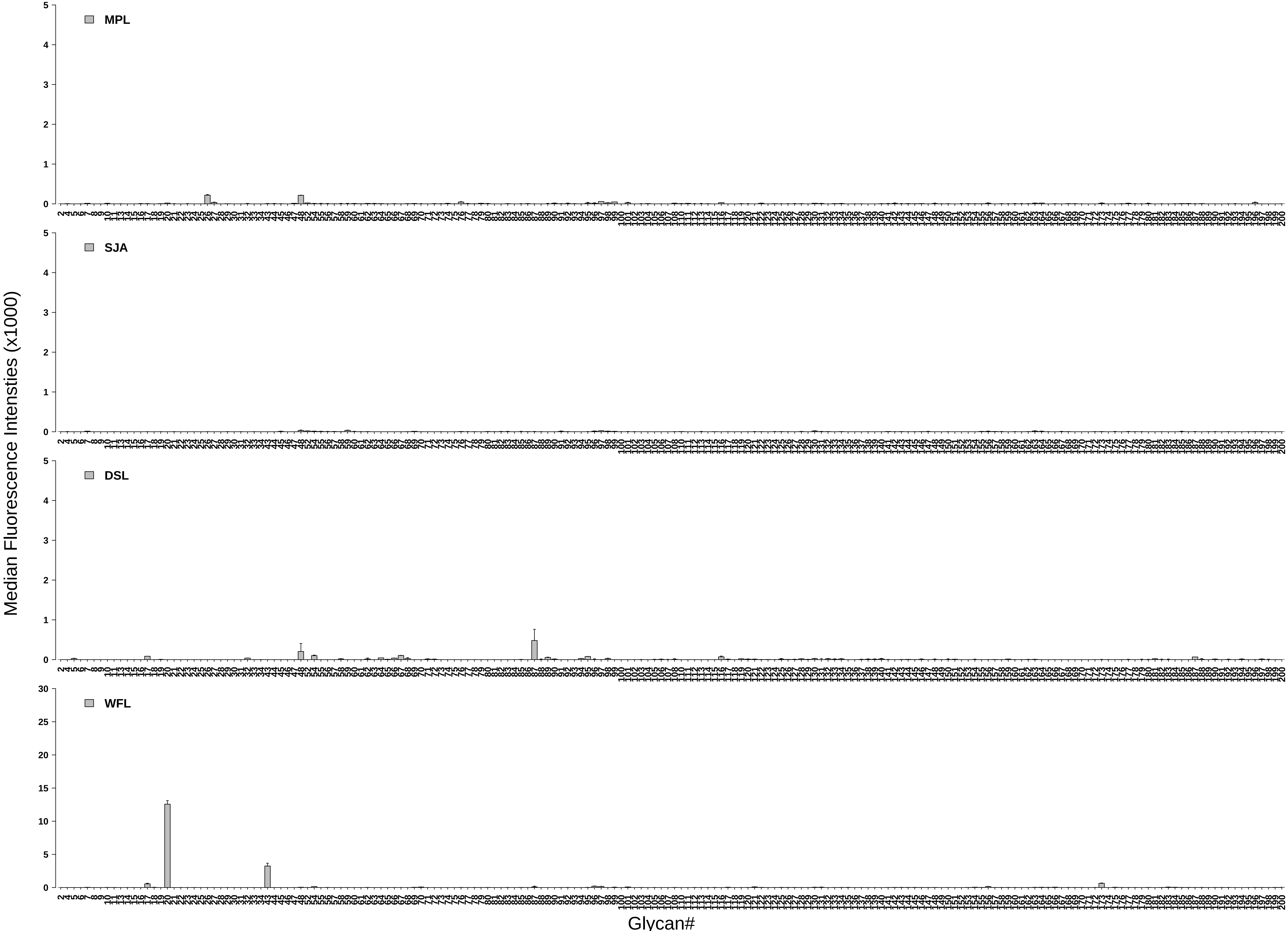

MPL
SJA
DSL
WFL
Median Fluorescence Intensities (x1000)
Glycan#

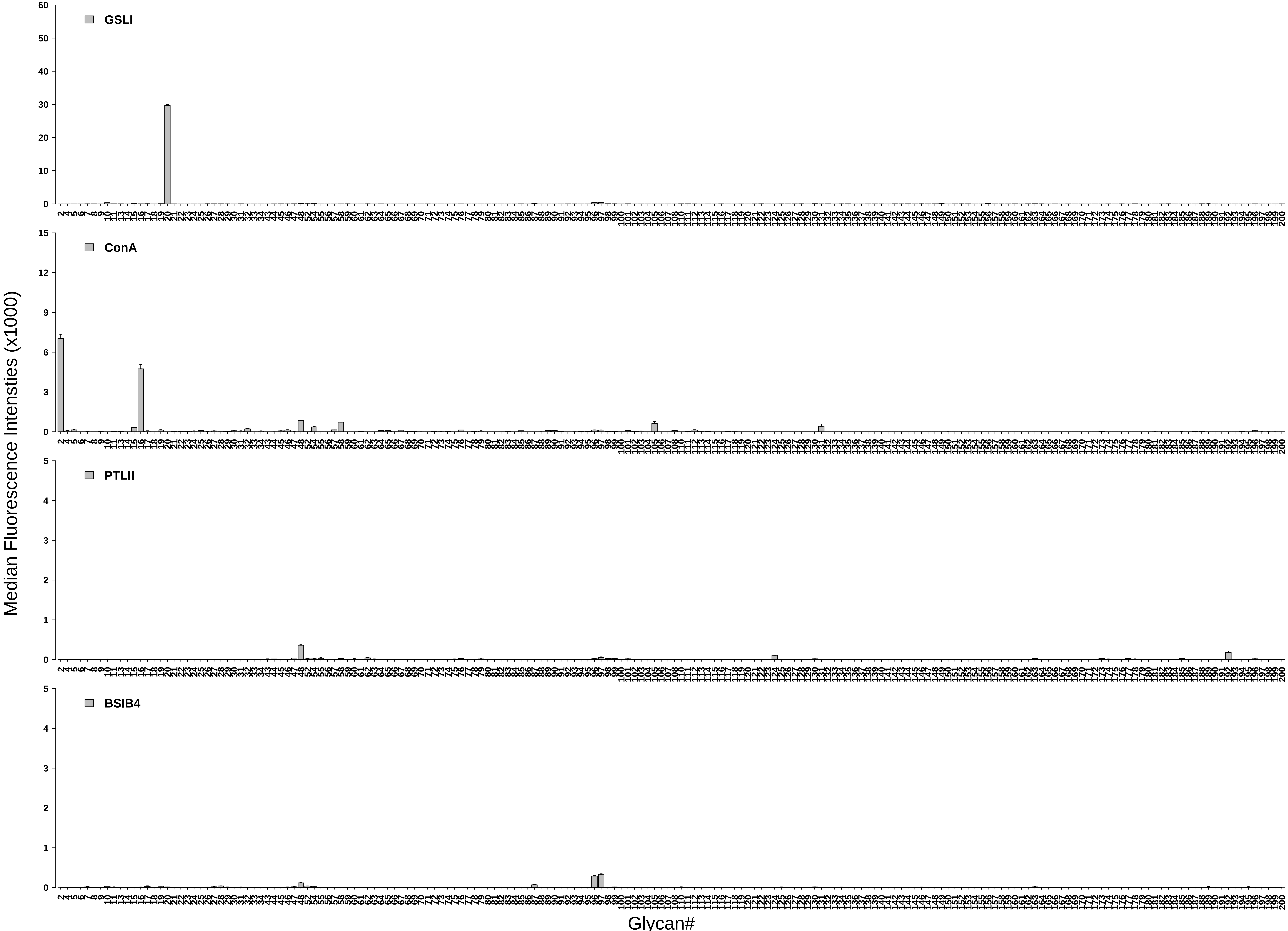

GSLI
ConA
PTLII
BSIB4
Median Fluorescence Intensties (x1000)
Glycan#

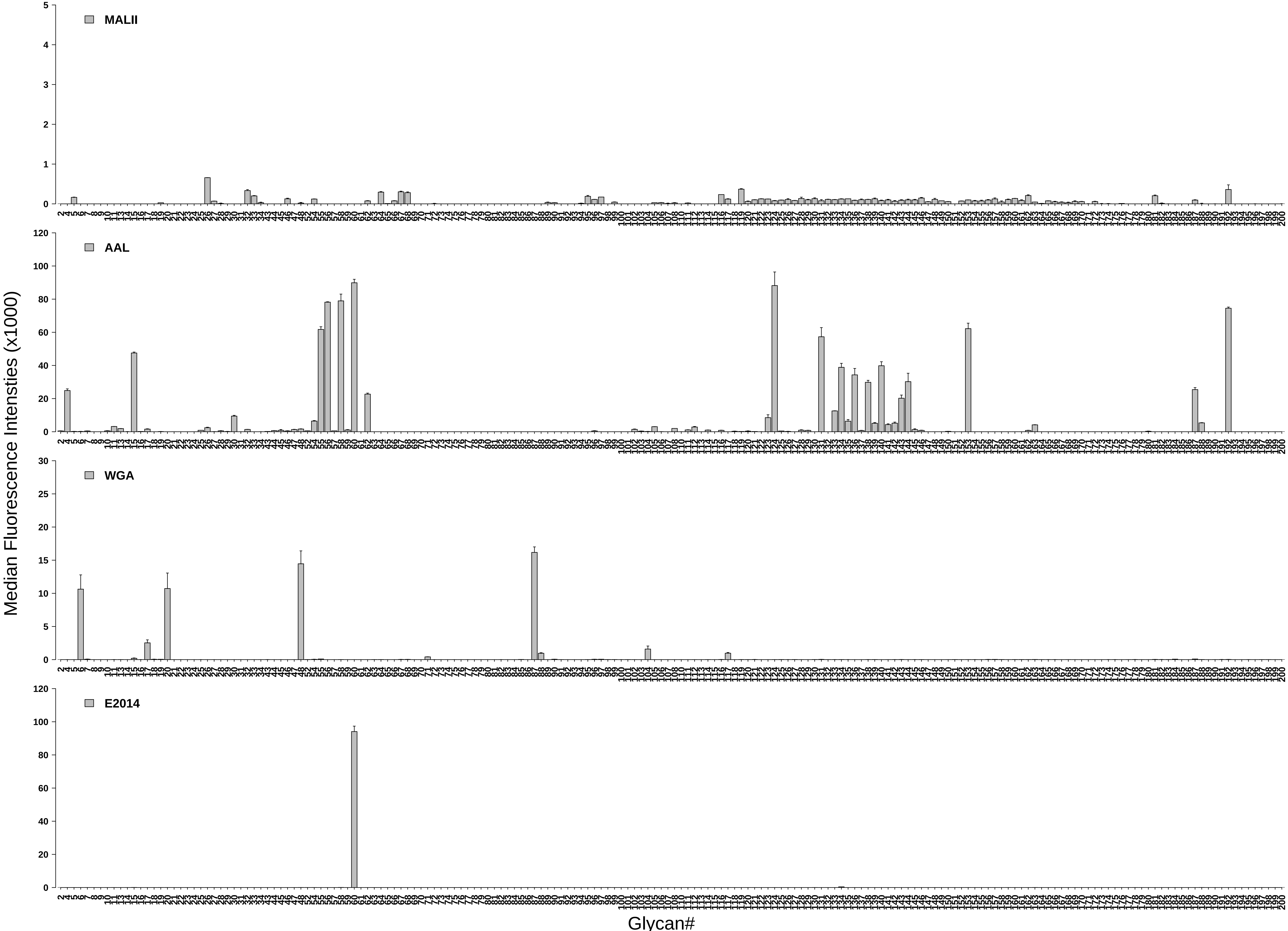
MALII
AAL
WGA
E2014
Median Fluorescence Intensties (x1000)
Glycan#

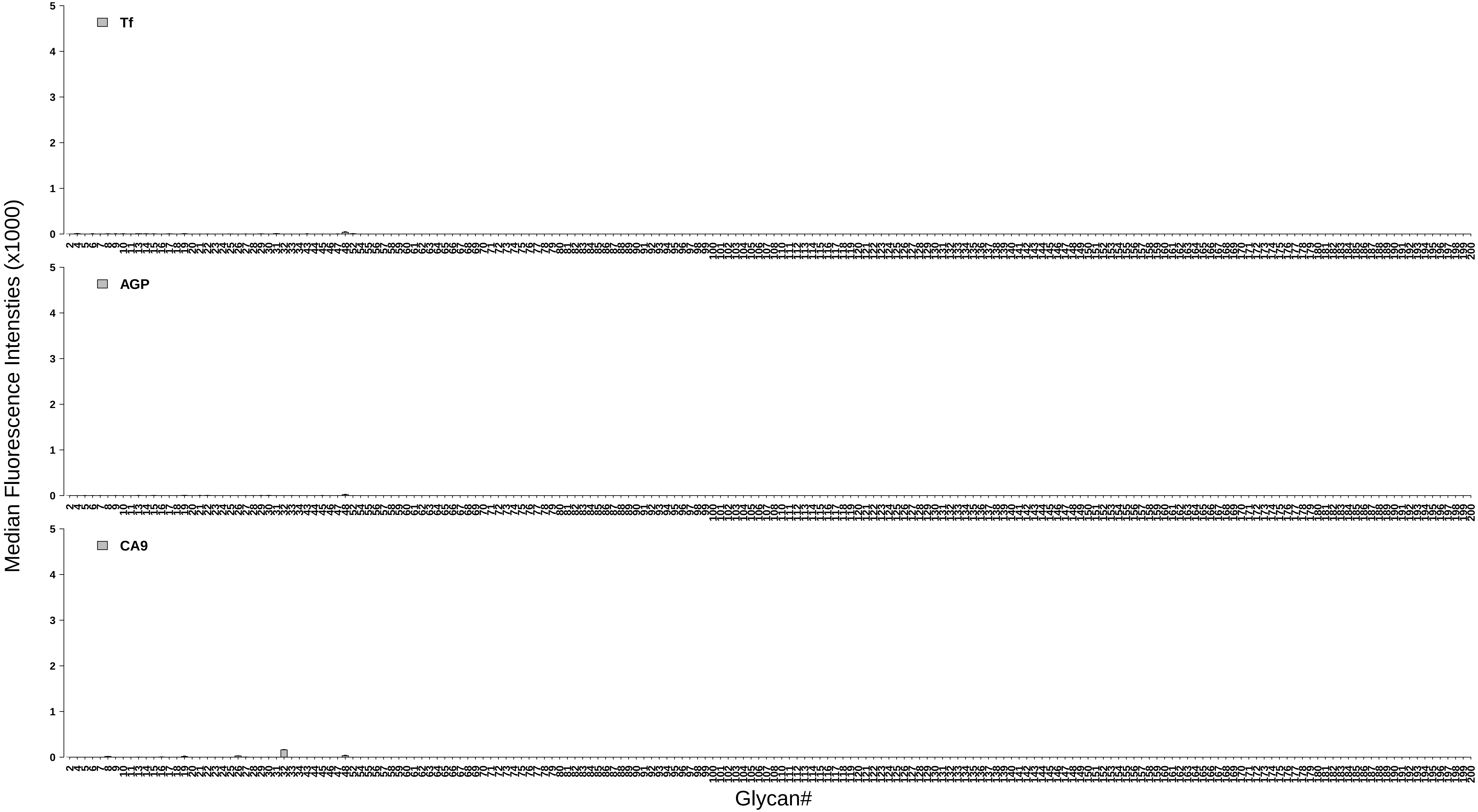

Supplementary Figure 3: Barplots displaying lectin binding to the glycans on MGBA. Briefly, 5ug/ml of lectins, except for AAL (1ug/ml), was used for the binding assay. The experiment was performed in duplicate wells and data were shown as mean MFI + SD. Several purified proteins were included as negative controls. An in-house anti-LeY IgG antibody (E2014) was used as a positive control. Data is presented and mean+SD of two replicates, each experiment was repeated 3 times.

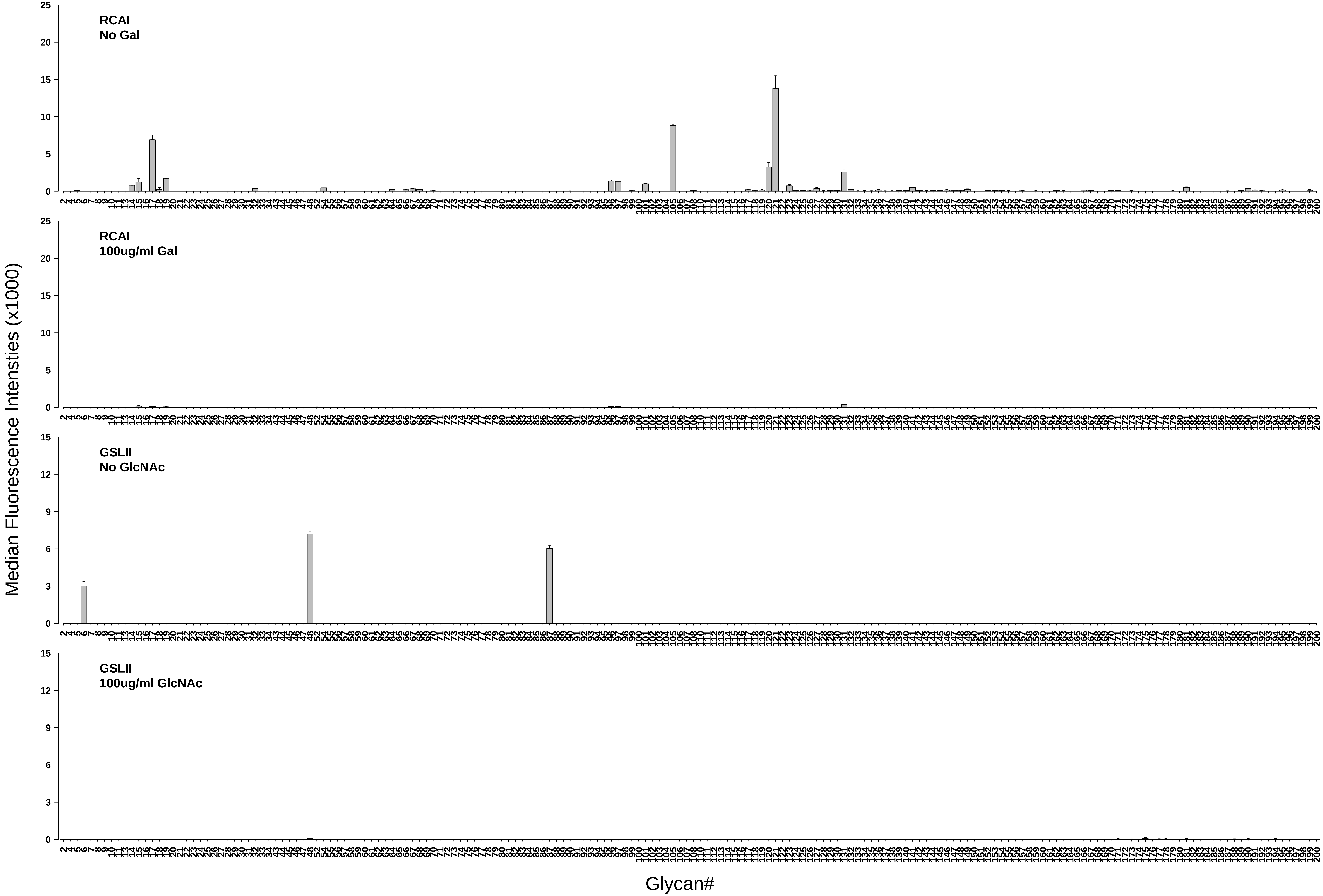
RCAI
No Gal
RCAI
100ug/ml Gal
GSLII
No GlcNAc
GSLII
100ug/ml GlcNAc
Median Fluorescence Intensties (x1000)
Glycan#

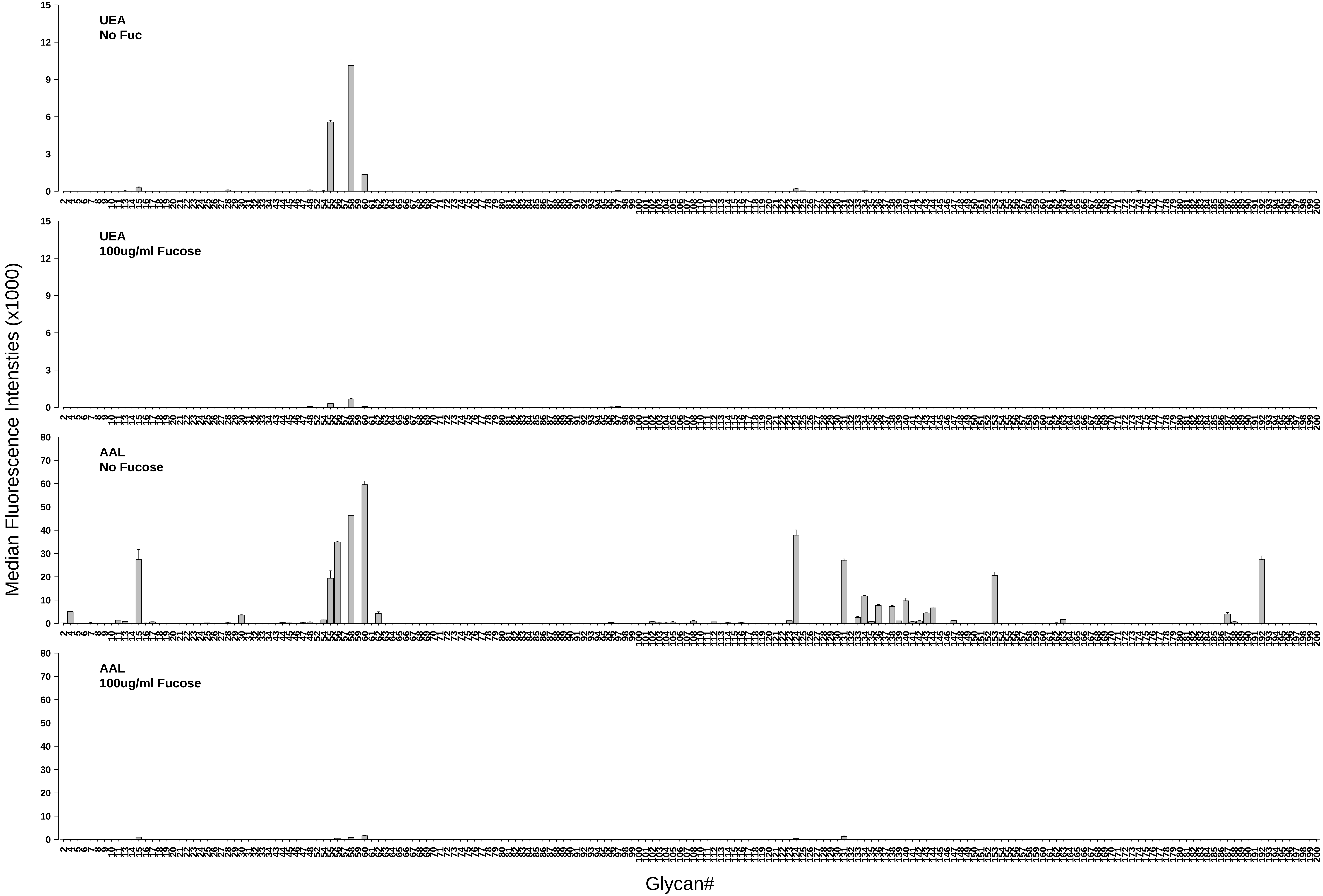

UEA
No Fuc
UEA
100ug/ml Fucose
AAL
No Fucose
AAL
100ug/ml Fucose
Median Fluorescence Intensities (x1000)
Glycan#

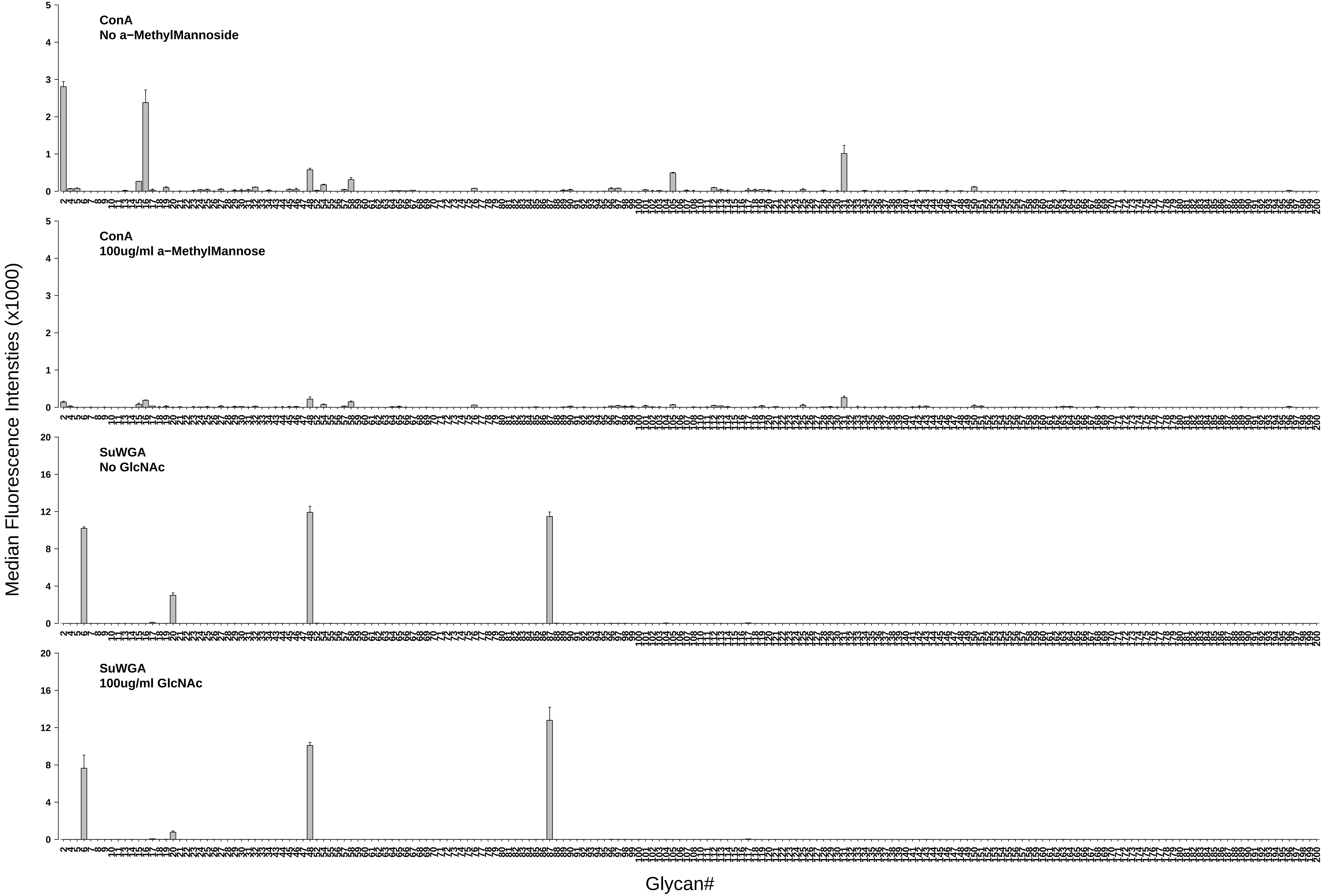
ConA
No a−MethylMannoside
ConA
100ug/ml a−MethylMannose
SuWGA
No GlcNAc
SuWGA
100ug/ml GlcNAc
Median Fluorescence Intensities (x1000)
Glycan#

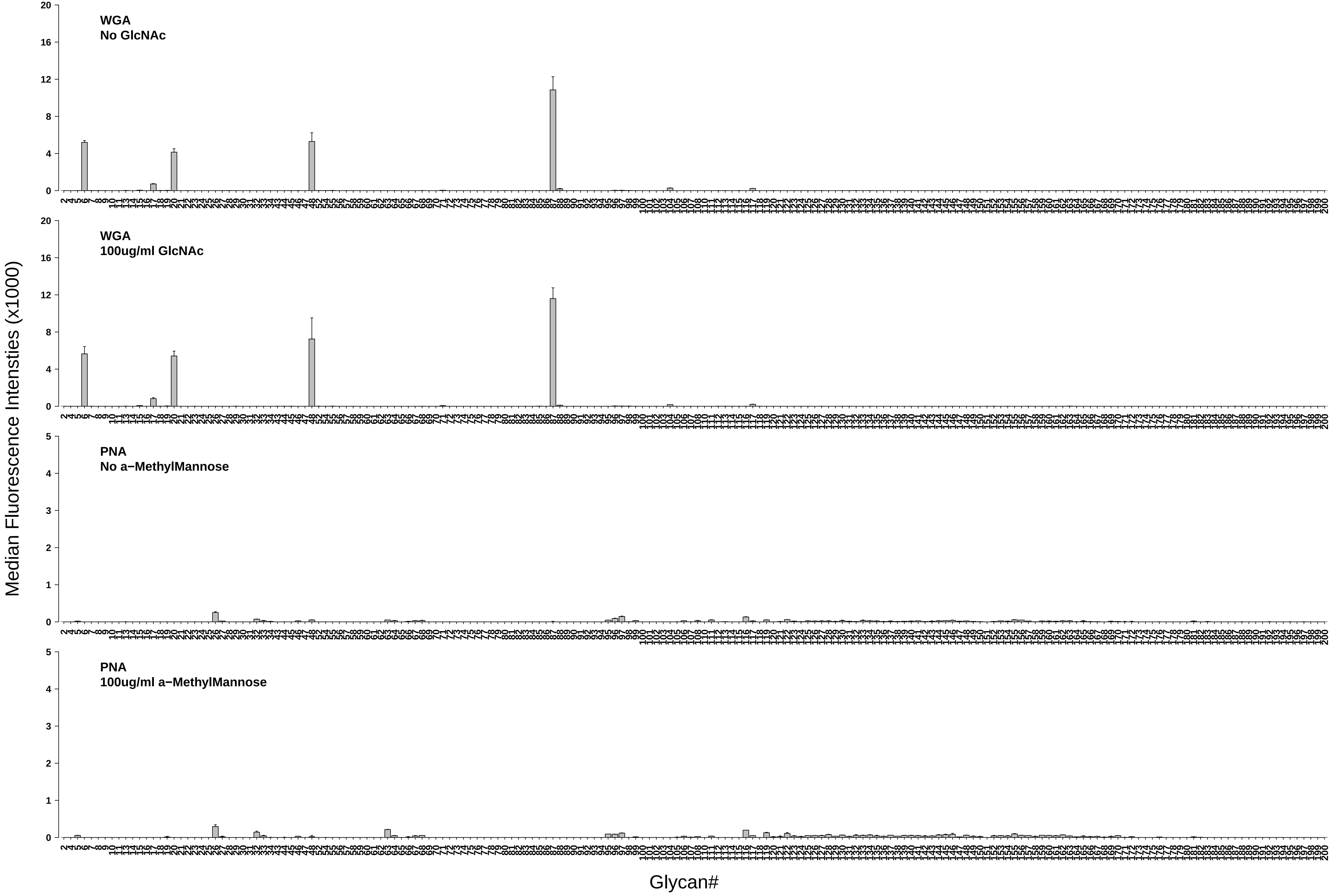
WGA
No GlcNAc
WGA
100ug/ml GlcNAc
PNA
No a−MethylMannose
PNA
100ug/ml a−MethylMannose
Median Fluorescence Intensties (x1000)
Glycan#

Supplementary Figure 4: Inhibition of lectin binding by monosaccharides. Lectins (5ug/ml), except for AAL (1ug/ml), and their respective inhibitory sugars (100μg/ml) were used in the inhibitory binding array. The data are presented as mean MFI + SD of the duplicate wells for each glycan.

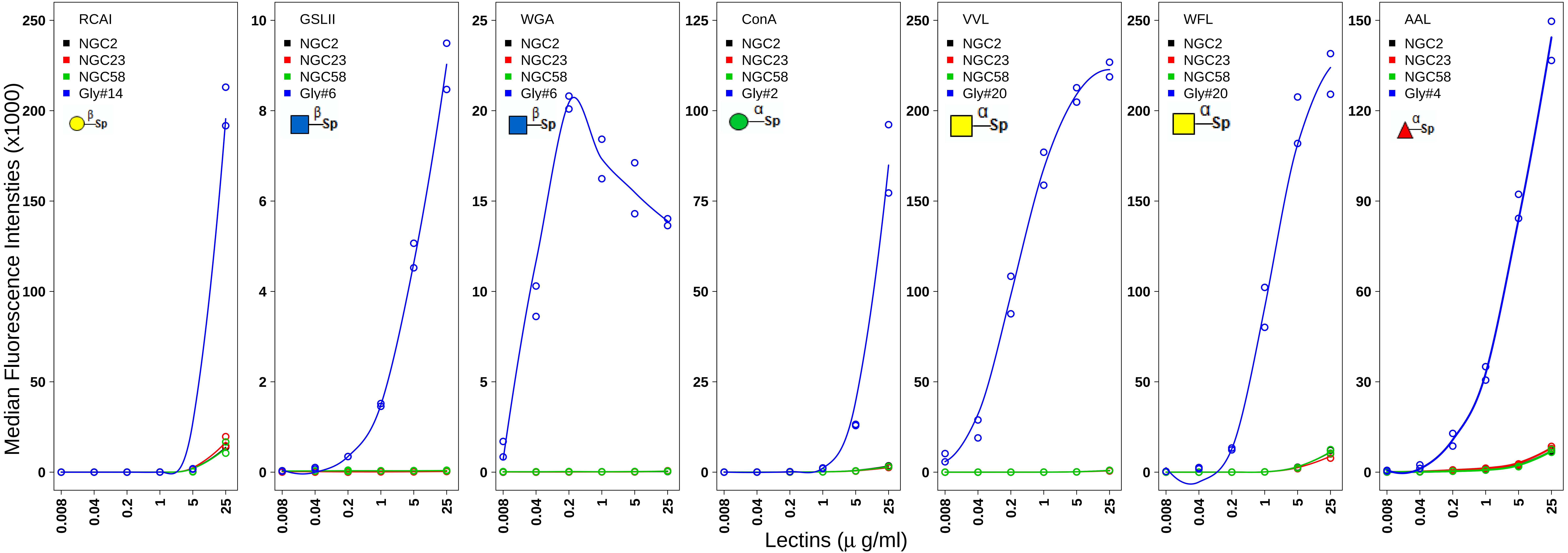

Supplementary Figure 5: Sensitivity of MGBA for lectin binding assays. Serial dilution of seven lectins were used for binding assays on MGBA. Mean MFI values (y-axis) were plotted against lectin concentration on x-axis. Lectin concentration values displayed are log2 transformed.

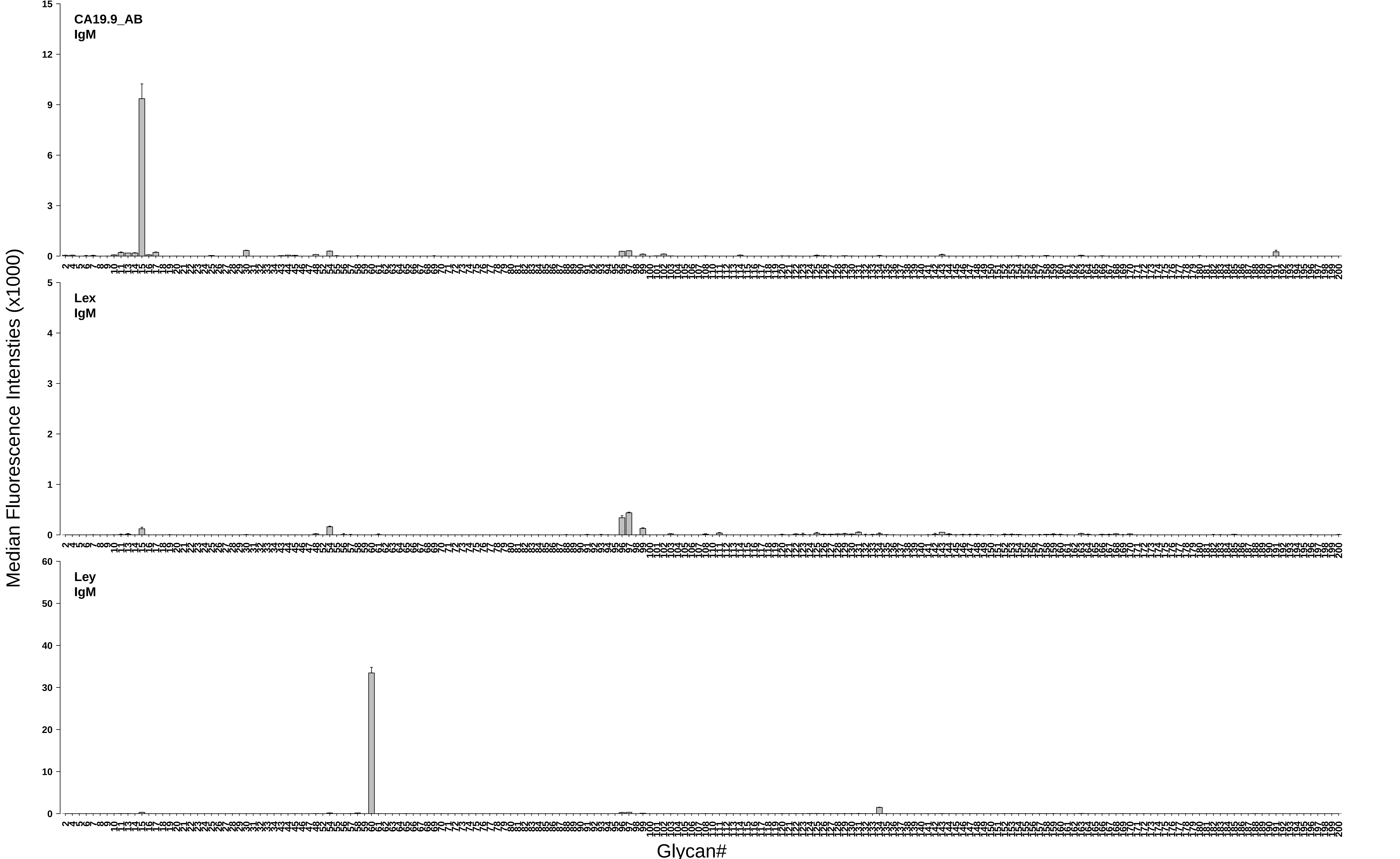
CA19.9_AB
IgM
Lex
IgM
Ley
IgM
Median Fluorescence Intensties (x1000)
Glycan#

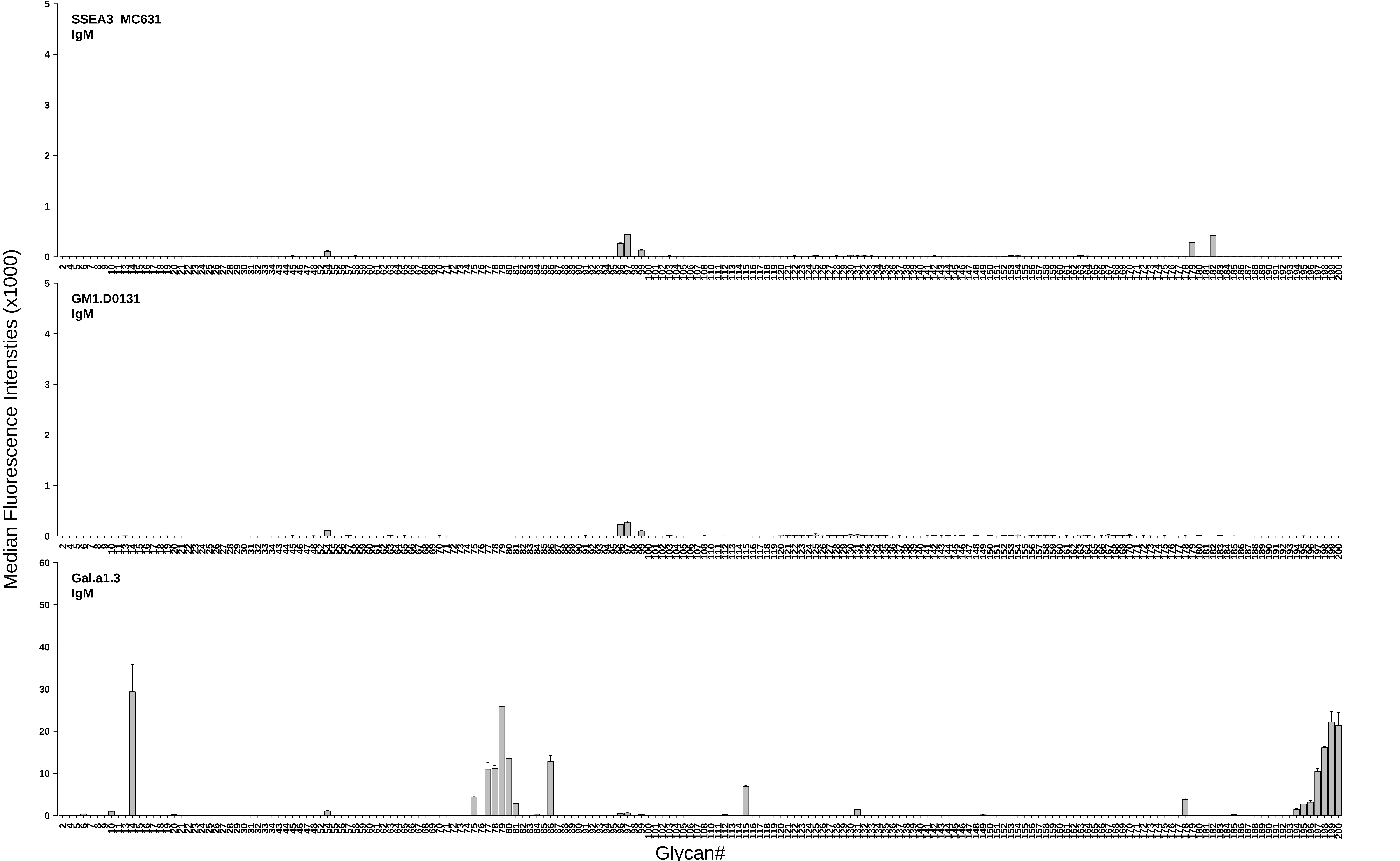
SSEA3_MC631
IgM
GM1.D0131
IgM
Gal.a1.3
IgM
Median Fluorescence Intensties (x1000)
Glycan#

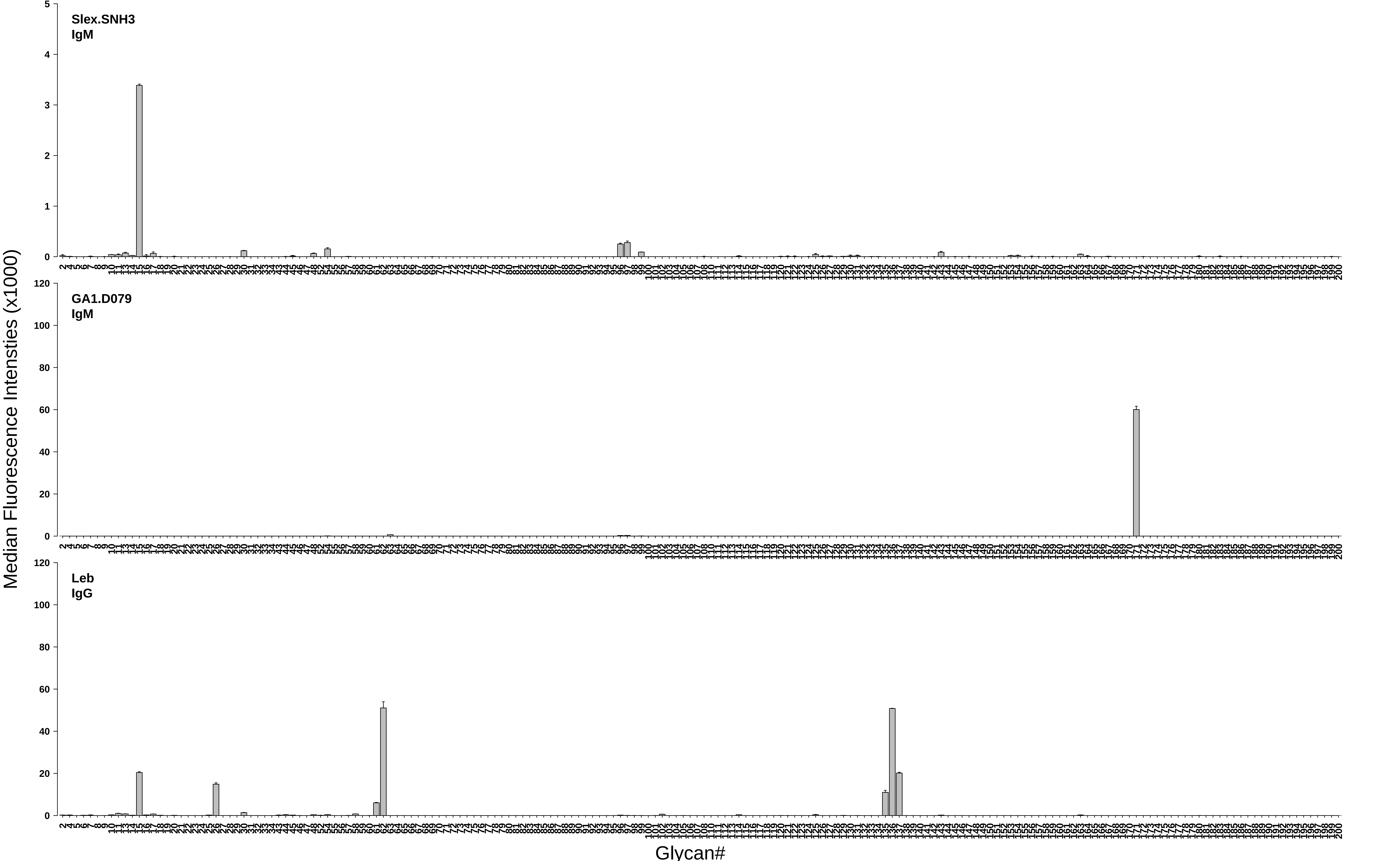

Slex.SNH3
IgM
GA1.D079
IgM
Leb
IgG
Median Fluorescence Intensties (x1000)
Glycan#

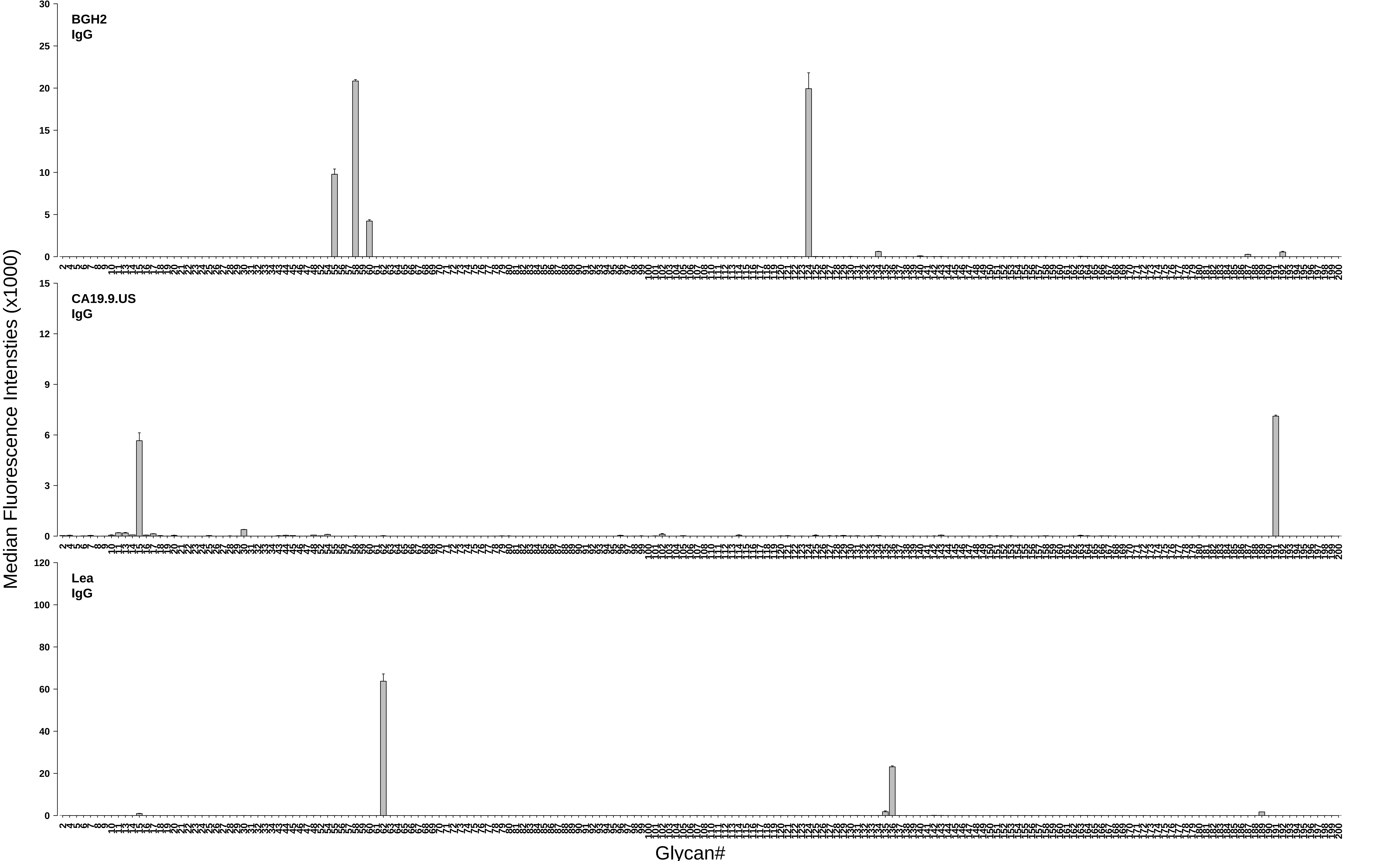
BGH2
IgG
CA19.9.US
IgG
Lea
IgG
Median Fluorescence Intensties (x1000)
Glycan#

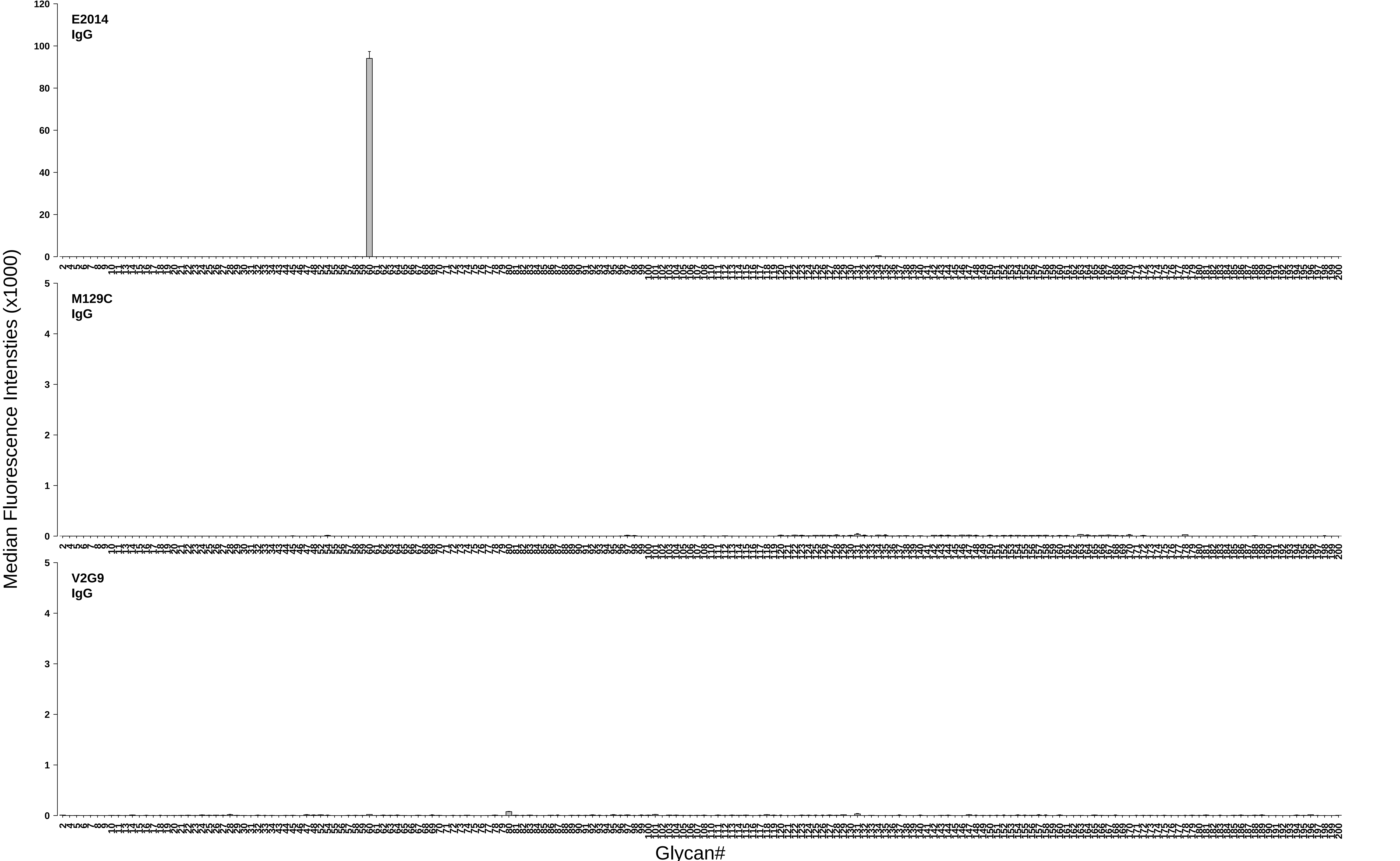
E2014
IgG
M129C
IgG
V2G9
IgG
Median Fluorescence Intensties (x1000)
Glycan#

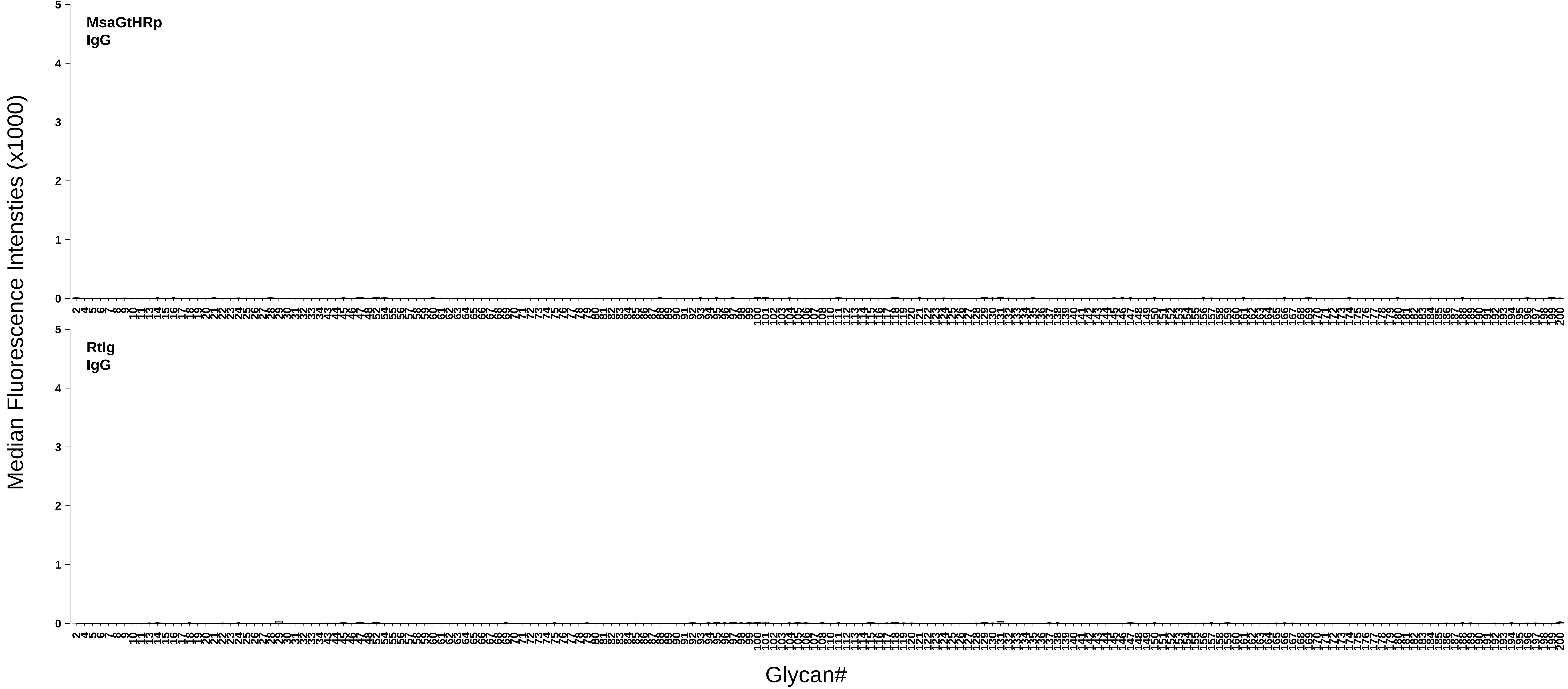

Supplementary Figure 6: Profiling of 13 anti-glycan antibodies on MGBA. Antibodies against proteins that are not known to bind glycans and total IgG from mouse and rat were used as negative controls. The data is presented as mean+SD of two replicates, each experiment was repeated 3 times.

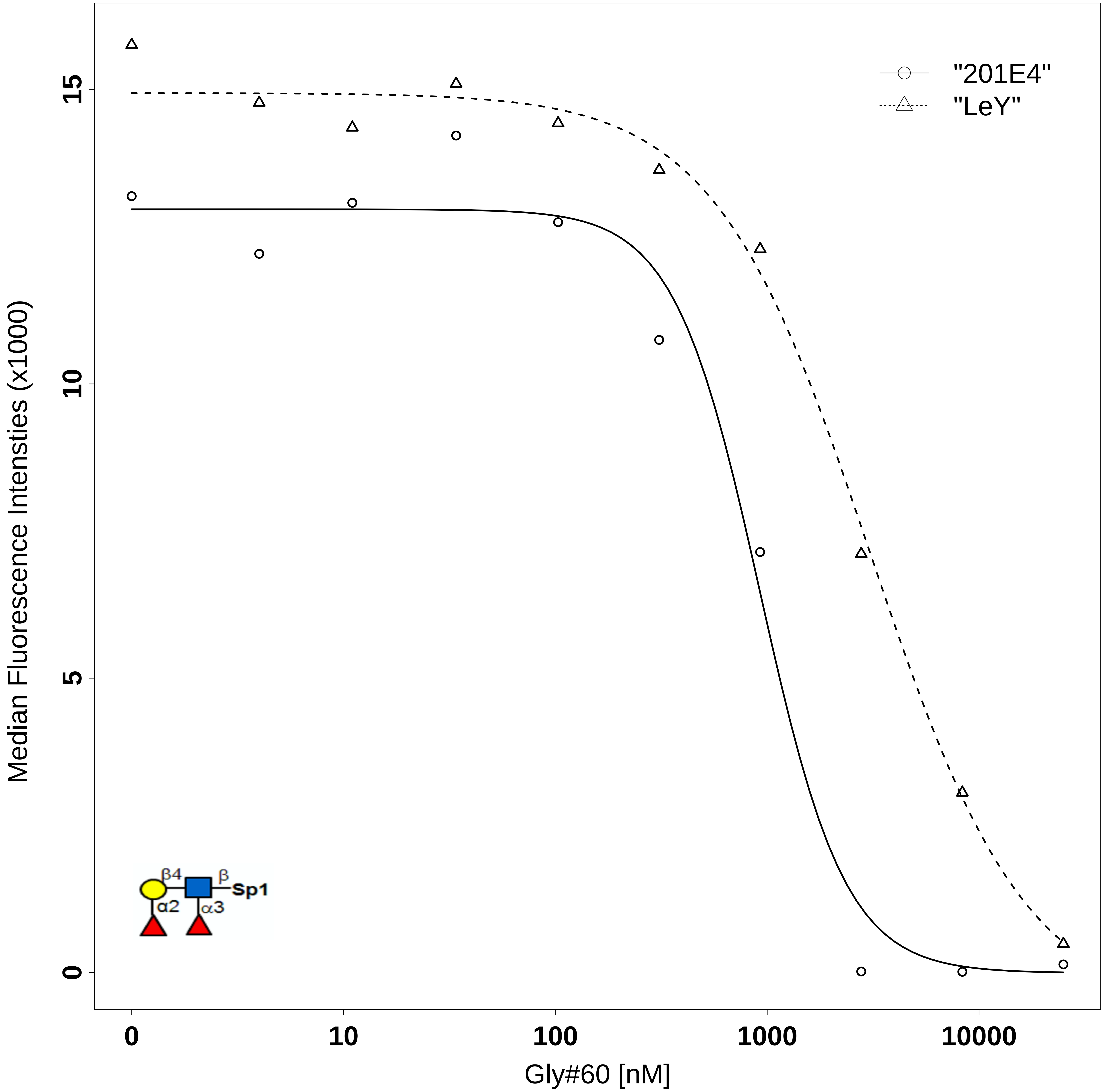

Supplementary Figure 7: Relative affinity determination of IgG and IgM antibodies that can bind Leyby competitive binding assay. Antibody affinity was obtained by determining the IC50. IC50 values for the in lab IgG type antibody (solid line) was 0.92 +/- 0.079 µM (mean +/- SD), whereas 3.0 +/- 0.61µM (mean +/- SD) for commercial IgM antibody (dashed line). The LeY structure used for determination of Kd values are presented in inset.

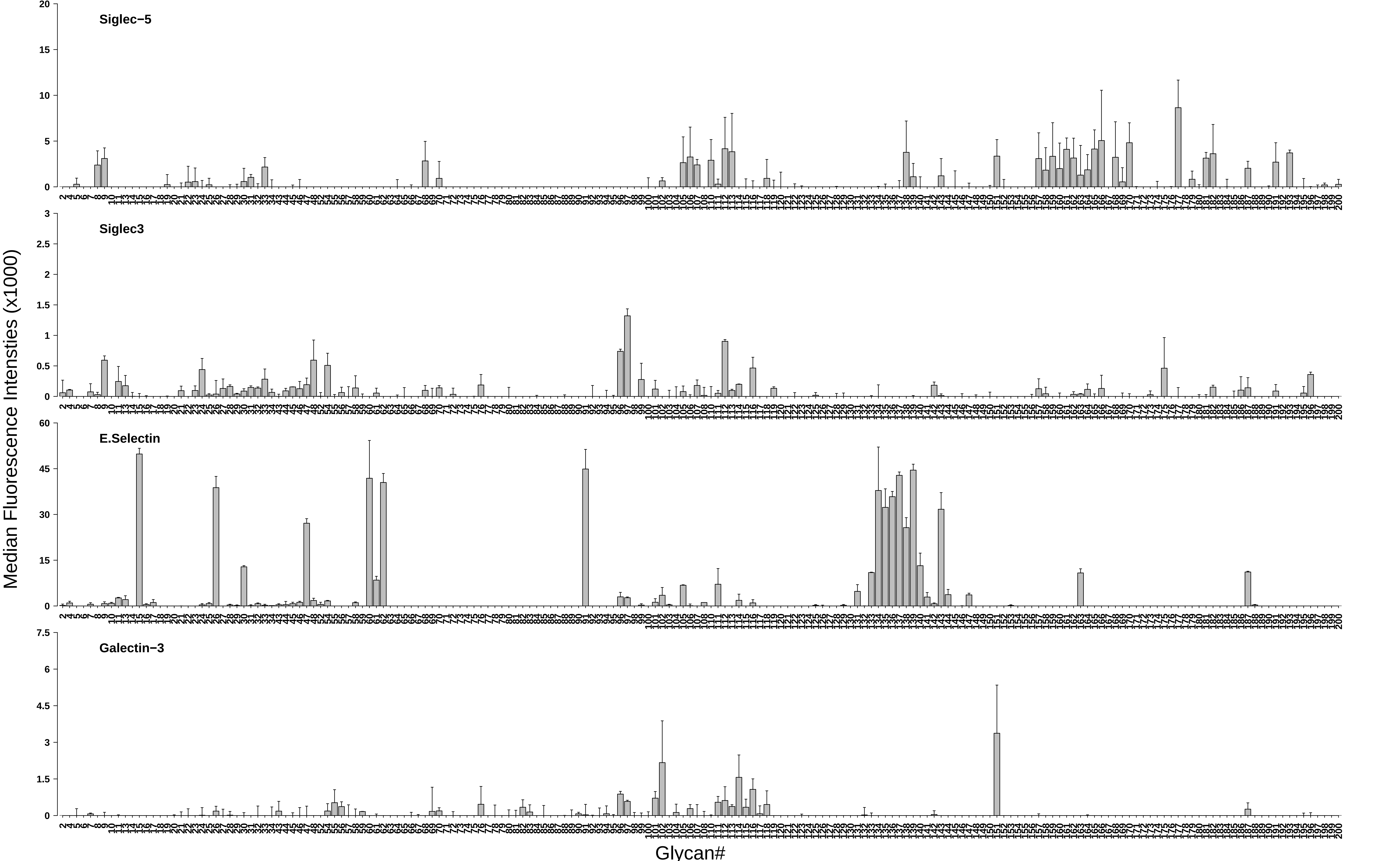
Siglec−5
Siglec3
E.Selectin
Galectin−3
Median Fluorescence Intensties (x1000)
Glycan#

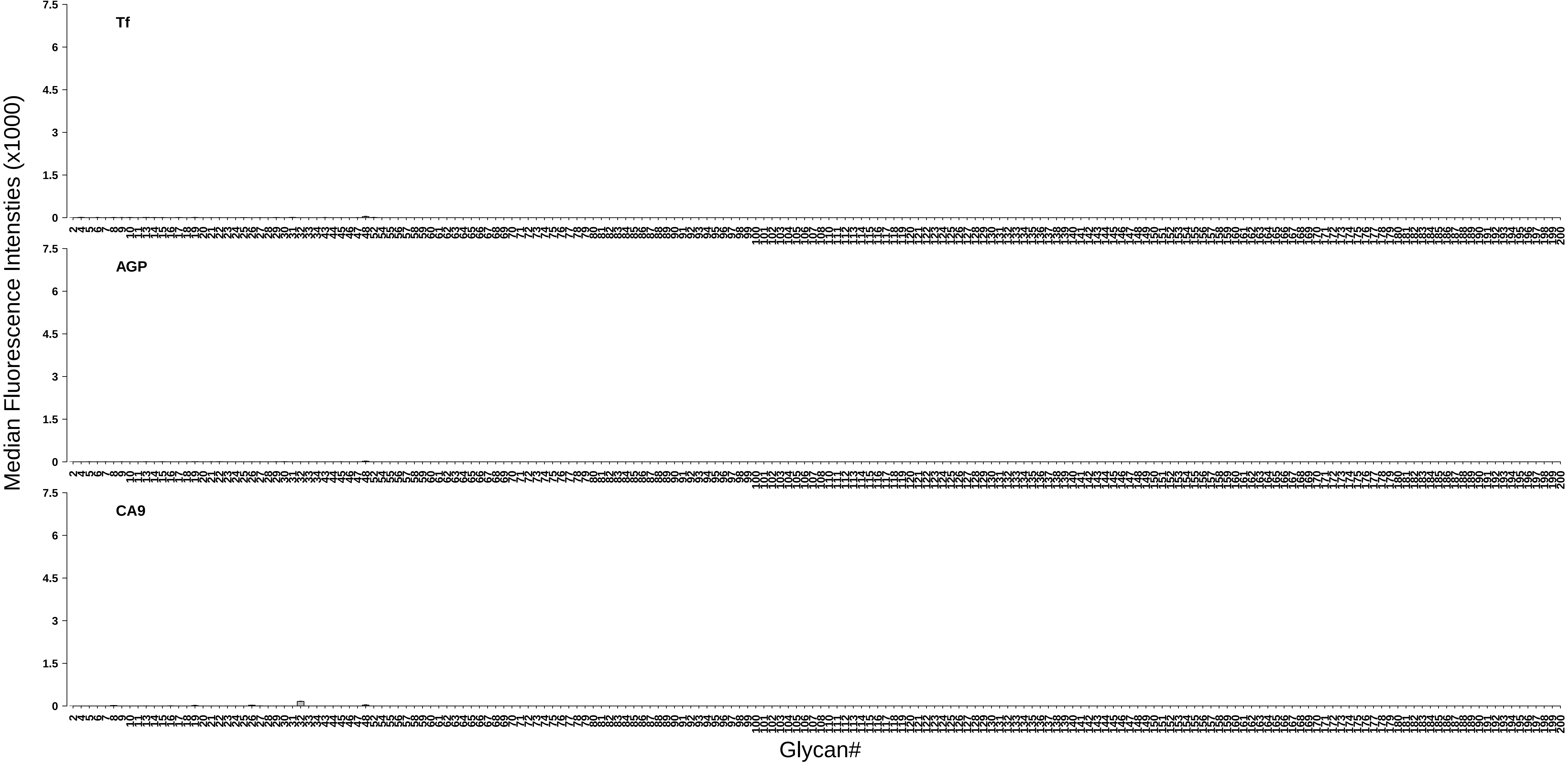

Supplementary Figure 8: Profiling of recombinant glycan binding protein of human (siglec-5, siglec-3 and galectin-3) and murine (E-selectin) origin. Purified serum proteins (Tf-transferrin, AGP- alpha-1 acid glycoprotein) and recombinant CA-9 were added as negative controls. Representative glycan # are added for identification purposes. The data is presented as mean+SD of median fluorescence intensities (y-axis) of two replicates, each experiment was repeated 3 times.

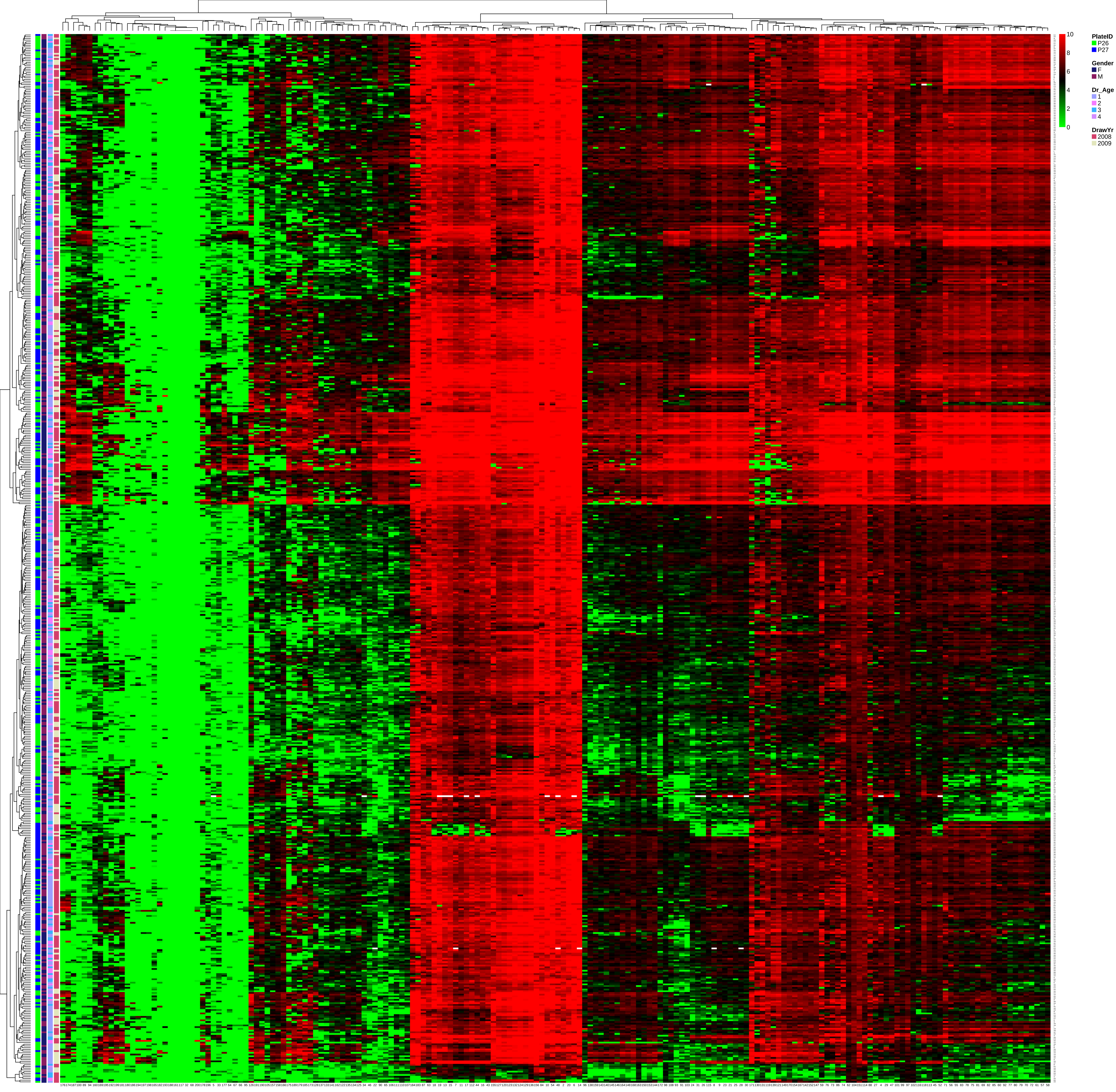
10
8
6
4
2
0
PlateID
P26
P27
Gender
F
M
Dr_Age
1
2
3
4
DrawYr
2008
2009

Supplementary Figure 9: Heatmap of anti-IgM antibody levels in 613 samples. Data are Log 2 transformed prior to unsupervised hierarchical clustering. Each row represents an individual sample and each column represents an individual glycan. IgM levels are represented by the color codes as indicated by the color bar. Serum IgM levels was used to cluster glycans (Cluster 1 – 5) as well as subjects (Cluster A – D). Cluster A, B, C and D contain 221 (36%), 54 (8.8%), 325 (53.1%), and 13 (2.1%) subjects, respectively (presented in Figure 5b). The sample clusters are not associated with demographic or clinical variables such as collection date, gender and age of the subjects, and batch of sample analyses.

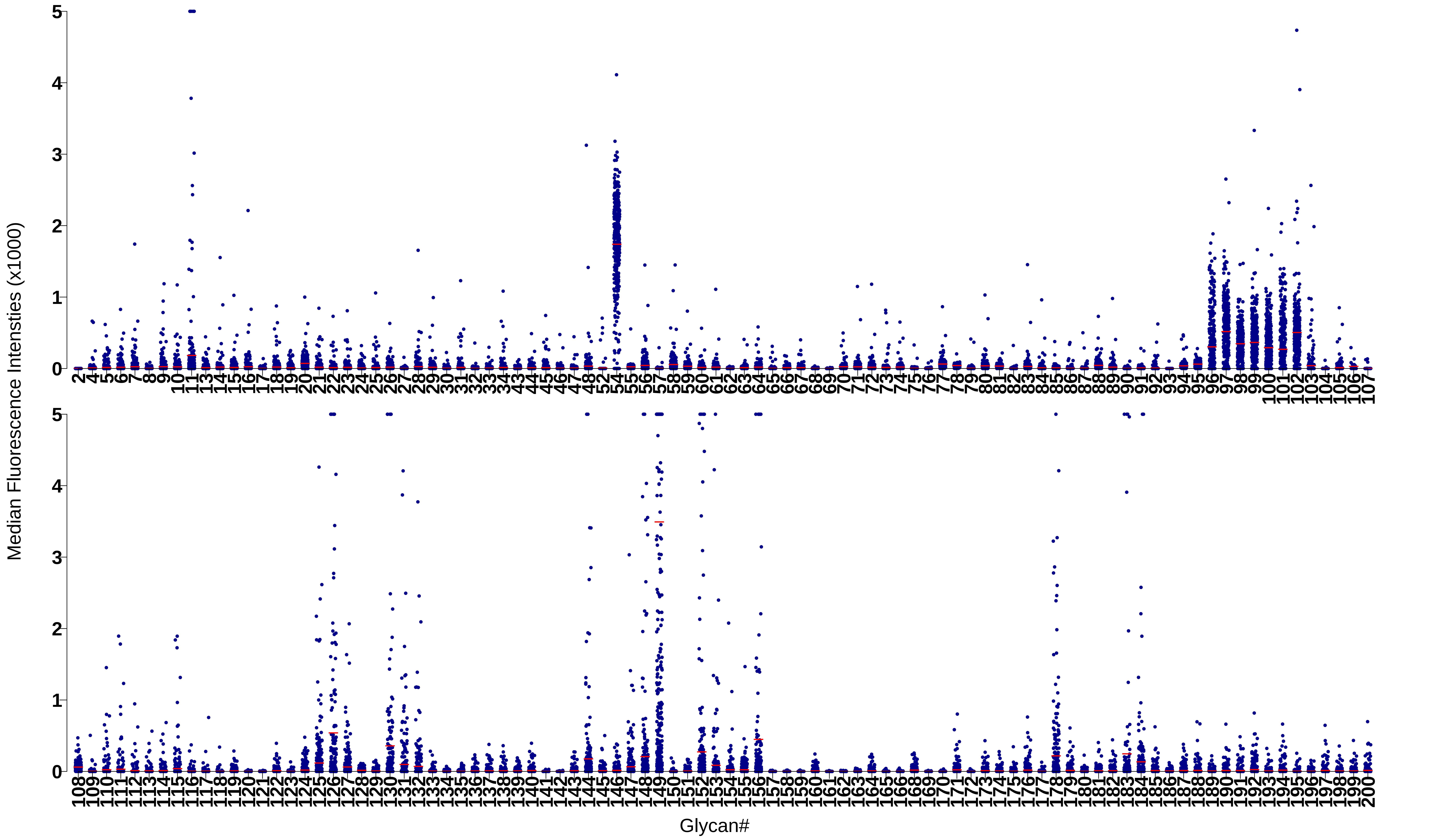
Median Fluorescence Intensities (x1000)
0 1 2 3 4 5
2 4 5 6 7 8 9 10 11 13 14 15 16 17 18 19 20 21 22 23 24 25 26 27 28 29 30 31 32 33 34 43 44 45 46 47 48 52 54 55 56 57 58 59 60 61 62 63 64 65 66 67 68 69 70 71 72 73 74 75 76 77 78 79 80 81 82 83 84 85 86 87 88 89 90 91 92 93 94 95 96 97 98 99 100 101 102 103 104 105 106 107
0 1 2 3 4 5
108 109 110 111 112 113 114 115 116 117 118 119 120 121 122 123 124 125 126 127 128 129 130 131 132 133 134 135 136 137 138 139 140 141 142 143 144 145 146 147 148 149 150 151 152 153 154 155 156 157 158 159 160 161 162 163 164 165 166 167 168 169 170 171 172 173 174 175 176 177 178 179 180 181 182 183 184 185 186 187 188 189 190 191 192 193 194 195 196 197 198 199 200
Glycan#

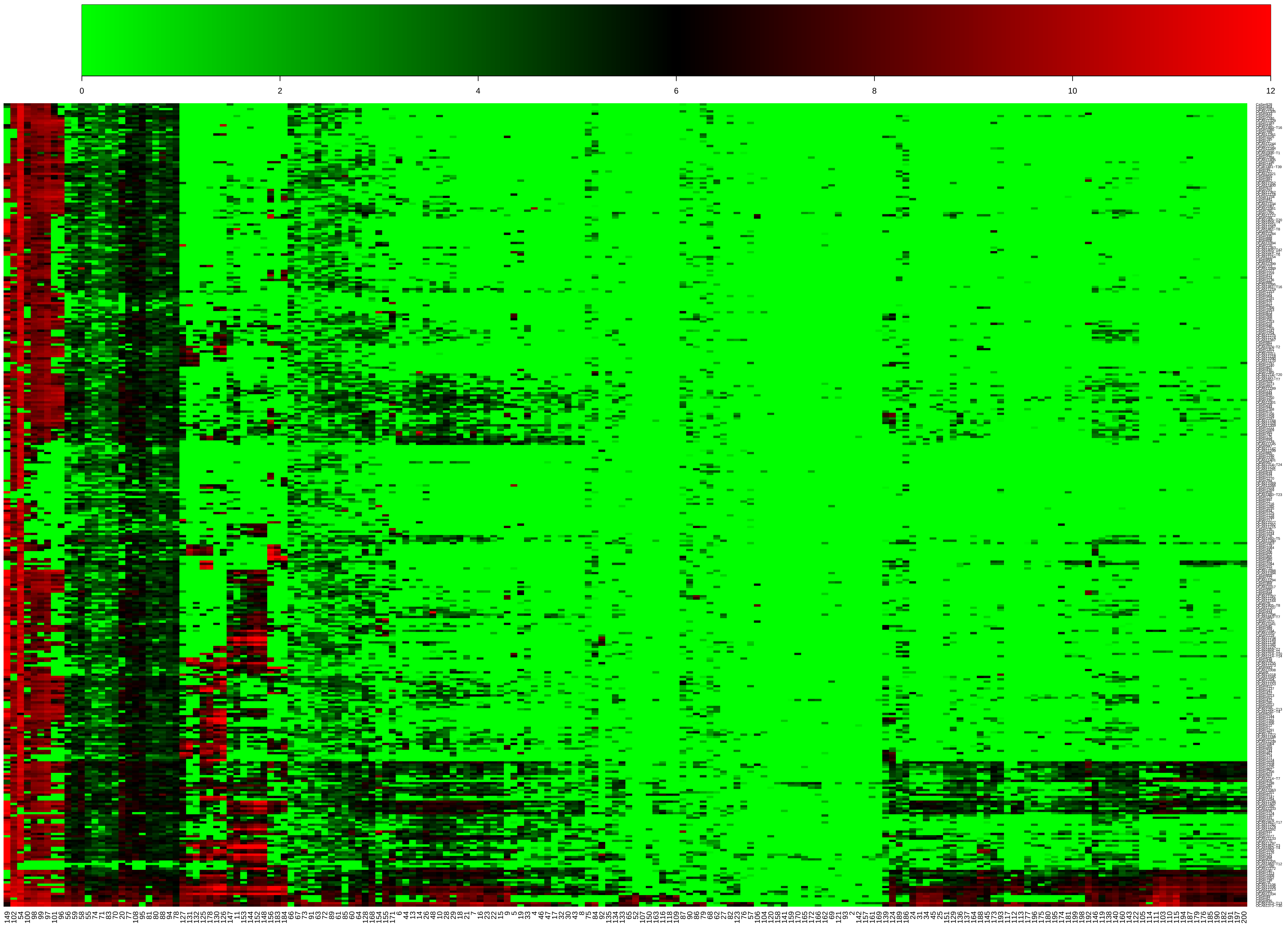
0
2
4
6
8
10
12

Supplementary Figure 10: Serum anti-glycan IgG antibodies in 348 ovarian cancer serum samples measured on MGBA. a) Dot plots of median fluorescence (MFI) for each glycan in 348 serum samples obtained from 119 patients at different time points. Samples with MFI above 5,000 were plotted at the 5,000 level. The mean of the MFI for the glycan is presented as a red line. b) Heatmap showing clusters of glycans based on serum IgG levels in the samples.

Supplementary Table 1: List of Glycan conjugated to create multiplex glycan bead array v1.0

Glycan IDs	Glycan ID	Name	**Structure**	Molecular Weight
2	GLYPW_002	α-Mannosyl derivative	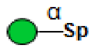	221.25
4	GLYPW_004	α-Fuc-Sp	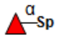	221.25
5	GLYPW_005	α-Rham-Sp	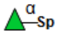	221.25
6	GLYPW_006	β-GlcNAc-Sp	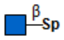	278.3
7	GLYPW_007	β-GalNAc-Sp	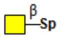	278.3
8	GLYPW_008	Neu5Ac-α-2,3-Gal-β-1,4-Glc-β-Sp	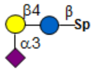	690.65
9	GLYPW_009	Neu5Ac-α-2,6-Gal-β-1,4-Glc-β-Sp	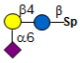	690.65
10	GLYPW_010	Gal-α-1,4-Gal-β-1,4-Glc-β-Sp	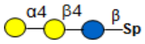	561.53
11	GLYPW_011	Gal-α-1,3-Gal-β-1,4-Glc-β-Sp	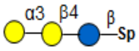	561.53
13	GLYPW_016	β-Glc-Sp	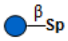	278.3
14	GLYPW_017	β-Gal-Sp	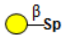	278.3
15	GLYPW_018	Gal-β-1,4-Glc-β-Sp	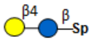	399.39
16	GLYPW_019	α-Man-Sp	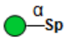	278.3
17	GLYPW_020	Gal-β-1,4-GlcNAc-β-Sp	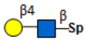	440.44
18	GLYPW_021	Gal-β-1,3-GlcNAc-β-Sp	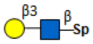	440.44
19	GLYPW_022	Glc-α-1,2-Gal-α-1,3-Glc-α-Sp	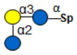	561.53
20	GLYPW_023	α-GalNAc-Sp	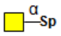	278.3
21	GLYPW_024	Gal-α-1,3-Gal-β-1,3-GlcNAc-β-Sp	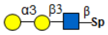	602.58
22	GLYPW_025	Neu5Ac-α-2,3-Gal-β-1,3-GlcNAc-β-Sp	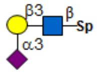	731.7
23	GLYPW_026	Neu5Ac-α-2,6-Gal-β-1,3-GlcNAc-β-Sp	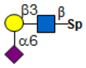	731.7
24	GLYPW_027	Neu5Gc-α-2,3-Gal-β-1,3-GlcNAc-β-Sp	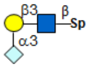	747.7

25	GLYPW_028	Neu5Gc-α-2,6-Gal-β-1,3-GlcNAc-β-Sp	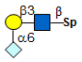	747.7
26	GLYPW_029	Gal-β-1,3-(Fuc-α-1,4)-GlcNAc-β-Sp	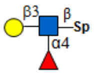	586.26
27	GLYPW_030	Gal-β-1,3-GalNAc-α-Sp	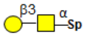	440.44
28	GLYPW_031	GlcNAc-β-1,3-Gal-β-1,4-Glc-β-Sp	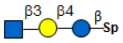	602.58
29	GLYPW_032	GalNAc-β-1,3-Gal-β-1,4-Glc-β-Sp	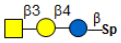	602.58
30	GLYPW_033	Neu5Gc-α-2,3-Gal-β-1,4-Glc-β-Sp	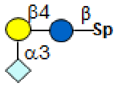	706.64
31	GLYPW_034	Neu5Gc-α-2,6-Gal-β-1,4-Glc-β-Sp	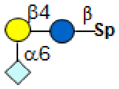	706.64
32	GLYPW_035	Gal-β-1,4-(Fuc-α-1,3)-Glc-β-Sp	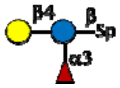	545.53
33	GLYPW_036	GlcNAc-β-1,6-GlcNAc-β-Sp	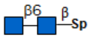	481.49
34	GLYPW_037	4-P-GlcNAc-β-1,6-GlcNAc-β-Sp	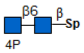	561.47
43	GLYPW_046	GalNAc-β-1,4 -Gal-β-1,4-Glc-β-Sp1 [Asialo GM2]	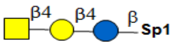	617.6
44	GLYPW_047	Gal-β-1,3 -GalNAc-β-1,3-Gal-β-Sp1	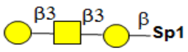	617.6
45	GLYPW_048	Glc-α-1,2-Gal-α-Sp	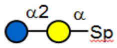	399.93
46	GLYPW_049	Gal-β-1,4 -(Fuc-α-1,3)-GlcNAc-β-1,3 -Gal-β-Sp1	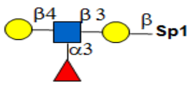	763.74
47	GLYPW_050	Neu5Ac-α-2,3-Gal-β-1,4 -(Fuc-α-1,3)-Glc-β-Sp1 [3-Sialyl-3-fucosyllactose/ F-SL]	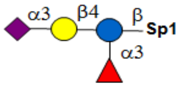	851.8
48	GLYPW_051	GlcNAc-β-1,4-GlcNAc-β-Sp1	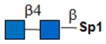	496.51
52	GLYPW_055	Gal-β-1,3-GalNAc-α-O-Ser (Gal-Tn-Antigen)	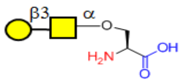	470.43
54	GLYPW_057	GalNAc-α-1,3-(Fuc-α-1,2)-Gal-β-Sp 1 [Blood A antigen trisaccharide]	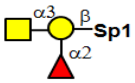	601.6
55	GLYPW_058	Fuc-α-1,2-Gal-β-1,4-GlcNAc-β-Sp1 [Blood H antigen trisaccharide Type 2]	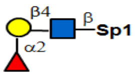	601.6

56	GLYPW_059	Gal-α-1,3-(Fuc-α-1,2)-Gal-β-Sp 1 [Blood B antigen trisaccharide]	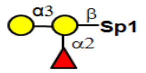	560.55
57	GLYPW_060	Fuc-α-1,2-Gal-β-1,3-GlcNAc-β-1,3-Gal-β-1,4-Glc-β-Sp [LNFP I]	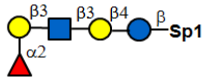	925.88
58	GLYPW_061	Fuc-α-1,2-Gal-β-1,4-Glc-β-Sp1 [Blood H antigen trisaccharide Type 5]	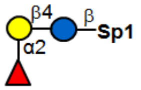	560.55
59	GLYPW_062	Gal-α-1,3-(Fuc-α-1,2)-Gal-β-1,4-Glc-β-Sp 1 [Blood B antigen tetrasaccharide]	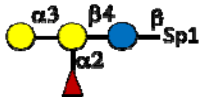	722.69
60	GLYPW_063	(Fuc-α-1,2)-Gal-β-1,4 -(Fuc-α-1,3)-GlcNAc-β-Sp1 [Lewis Y]	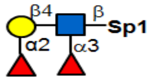	747.74
61	GLYPW_064	(Fuc-α-1,2)-Gal-β-1,3-(Fuc-α-1,4)-GlcNAc-β-Sp1 [Lewis B]	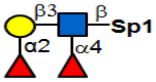	747.74
62	GLYPW_065	Gal-β-1,3-(Fuc-α-1,4)-GlcNAc-β-1,3-Gal-β-1,4 -(Fuc-α-1,4)-Glc-β-Sp1 [Lewis A]	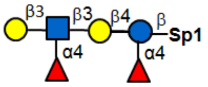	1072.02
63	GLYPW_066	Gal-β-1,3-GalNAc-β-Sp 1 (T-antigen)	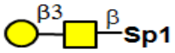	455.46
64	GLYPW_067	Gal-β-1,3-(Neu5Ac-α-2,6)-GalNAc-β-Sp	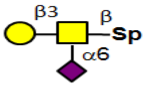	731.7
65	GLYPW_068	Neu5Ac-α-2,6-Gal β-1,3-GalNAc-β-Sp	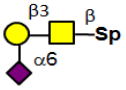	731.7
66	GLYPW_069	Neu5Ac-α-2,6-Gal β-1,3-(Neu5Ac-α-2,6)-GalNAc-β-Sp	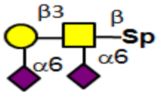	1022.95
67	GLYPW_070	Neu5Ac-α-2,3-Gal β-1,3-(Neu5Ac-α-2,6-)-GalNAc-β-Sp	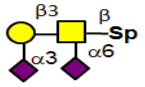	1022.95
68	GLYPW_071	Neu5Ac-α-2,6-(Neu5Ac-α-2,3)-Gal β-1,3-GalNAc-β-Sp	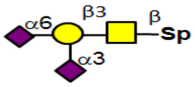	1022.95
69	GLYPW_072	GalNAc-β-1,4-(Neu5Ac-α-2,3)-Gal β-1,4-Glc-β-Sp [GM2]	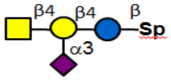	893.84
70	GLYPW_073	GalNAc-β-1,4-(Neu5Ac-α-2,8-Neu5Ac-α-2,3)-Gal β-1,4-Glc-β-Sp [GD2]	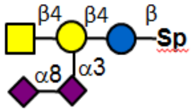	1185.09

71	GLYPW_074	Gal-α-1,4-Gal β-1,4-GlcNAc-β-Sp1	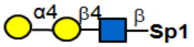	617.26
72	GLYPW_075	D-Rhamnose-sp	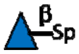	221.25
73	GLYPW_076	Glc-α-1,4-Glc-β-Sp1	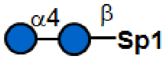	414.41
74	GLYPW_077	Glc-α-1,6-Glc-α-1,4-Glc-β-Sp1	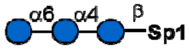	576.55
75	GLYPW_078	Maltotriose-Sp1	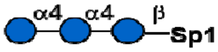	576.55
76	GLYPW_079	Glc-α-1,6-Glc-α-1,6-Glc-β-Sp1	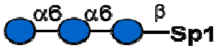	576.55
77	GLYPW_080	Maltotetraose-β-Sp1	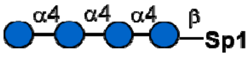	738.69
78	GLYPW_081	Maltopentaose-β-Sp1	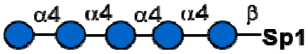	900.83
79	GLYPW_082	Maltohexaose-β-Sp1	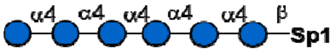	1062.97
80	GLYPW_083	Maltoheptaose-β-Sp1	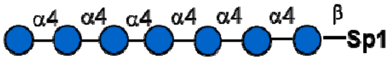	1225.11
81	GLYPW_084	Acarbose-β-Sp1	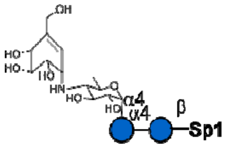	717.71
82	GLYPW_085	D-pentamannuronic acid	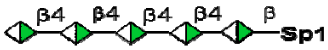	970.74
83	GLYPW_086	L-pentaguluronic acid	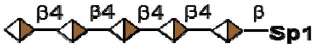	970.74
84	GLYPW_087	D-cellose-Sp1	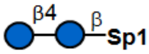	414.41
85	GLYPW_088	Cellopentaose-Sp1	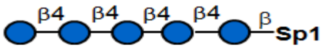	900.83
86	GLYPW_089	β-1,4-Xylotetrose-Sp1	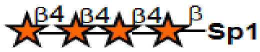	618.58
87	GLYPW_090	Chitin-trisaccharide-Sp1	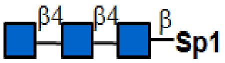	699.3174
88	GLYPW_091	GlcNAc-α-1,3-(Glc-α-1,2-Glc-α-1,2)Gal-α-1,3-Glc-α-Sp	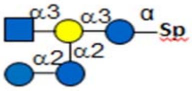	926.86
89	GLYPW_092	GlcNAc-β-1,2-Man-α-Sp	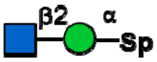	440.44

90	GLYPW_093	Gal-β-1,4-(6S)GlcNAc-β	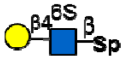	520.5
91	GLYPW_094	Neu5Ac-α-2,3-Gal-β-Sp1	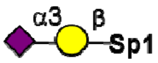	543.52
92	GLYPW_095	Gal-α-1,3-Gal-β-Sp1	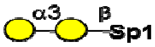	414.41
93	GLYPW_096	D-GlcA-Sp	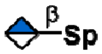	251.23
94	GLYPW_097	GlcA-β-1–4-GlcNAc-α-1–4-GlcA-β-SP	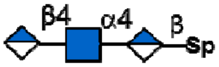	630.55
95	GLYPW_098	GlcNAc-α-1–4-GlcA-β-1–4-GlcNAc-α-1–4-GlcA-β-SP	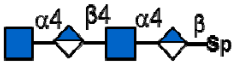	833.74
96	GLYPW_099	Tobramycin	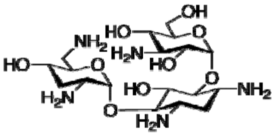	467.51
97	GLYPW_100	Amikacin	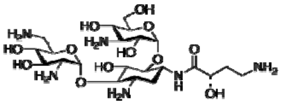	585.6
98	GLYPW_101	Gentamicin Sulfate	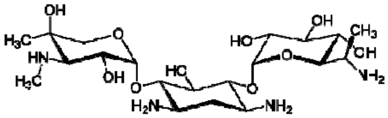	575.67
99	GLYPW_102	Kanamycin sulfate	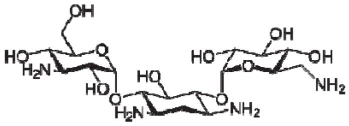	582.58
100	GLYPW_103	Geneticin Disulfate Salt (G418)	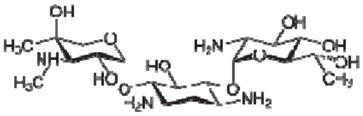	692.71
101	GLYPW_104	Neomycin trisulfate	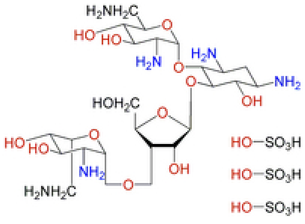	908.88

102	GLYPW_105	Sisomycin Sulphate	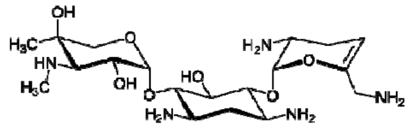	692.72
103	GLYPW_106	Gal-β-1,3-GalNAc-β-1,3-Gal-α-1,4-Gal-β-1,4-Glc-β-Sp	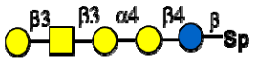	926.86
104	GLYPW_107	GlcNAc-β-1,6-(GlcNAc-β-1,3)-Gal-β-1,4-Glc-β-Sp1	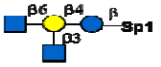	820.79
105	GLYPW_108	SGP [N-glycan]	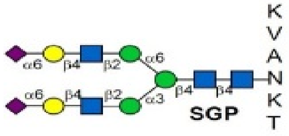	2865.76
106	GLYPW_109	Neu5Ac-α-2,8-Neu5Ac-α-2,3-Gal β-1,4-Glc-β-Sp [GD3]	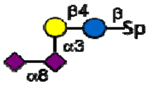	981.9
107	GLYPW_110	Neu5Gc-α-2,8-Neu5Ac-α-2,3-Gal β-1,4-Glc-β-Sp [GD3 (Gc-Ac)]	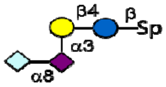	997.9
108	GLYPW_111	KDN-α-2,8-Neu5Ac-α-2,3-Gal β-1,4-Glc-β-Sp [GD3 (KDN-Ac)]	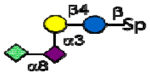	940.85
109*	GLYPW_112	Neu5Ac-α-2,8-Neu5Gc-α-2,3-Gal β-1,4-Glc-β-Sp [GD3 (Ac-Gc)]	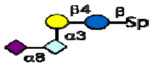	997.9
110	GLYPW_113	α-2,8-Neu5Ac-α-2,8-Neu5Ac-α-2,8-Neu5Ac-α-2,3-Gal-β-1,4-Glc-Sp2 [GT3]	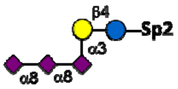	1594.52
111	GLYPW_114	Neu5Ac-α-2,8-Neu5Ac-α-2,8-Neu5Ac-α-2,8 Neu5Ac-α-2,3-Gal β-1,4-Glc-β-Sp [Neu5Ac-α-2,8-GT3]	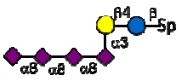	1564.41
112	GLYPW_115	Gal β-1,3-GalNAc-β-1,4-(Neu5Ac-α-2,3)-Gal β-1,4-Glc-β- SP1 [GM1a]	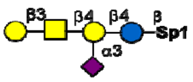	1070.99
113	GLYPW_116	Gal β-1,3-GalNAc-β-1,4-(Neu5Gc-α-2,3)-Gal β-1,4-Glc-β-SP1 [GM1a (Gc)]	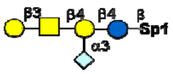	1086.99
114	GLYPW_117	GalNAc-β-1,4-(Neu5Gc-α-2,8-Neu5Ac-α-2,3)-Gal β-1,4-Glc-β-Sp [GD2 (Gc)]	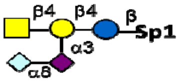	1216.11

115	GLYPW_118	GalNAc-β-1,4-(Neu5Ac-α-2,3)-Gal β-1,4-Glc-β-Sp [GM2 (KDN)]	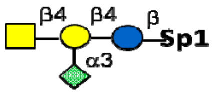	1070.99
116	GLYPW_119	Neu5Ac-α-2,3-Gal-β-1,4-(6S) GlcNA-Sp	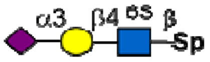	811.76
117	GLYPW_120	GalNAc-β-1,4-GlcNAc-β $CH_2CH_2NH_2$ [LacdiNAc]	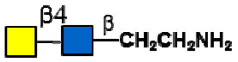	467.47
118	GLYPW_121	GlcNAc-β-1,6-(Gal-β-1,3)-GalNAc-α-O-Ser	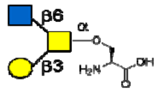	673.62
119	GLYRDC_126	Galβ1-3GalNAcβ1-3Gal -AEAB	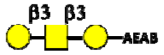	545.48
120	GLYRDC_022	Galβ1-4GlcNAcβ1-3Galβ1-4GlcNAcβ1-3Galβ1-4Glc -AEAB	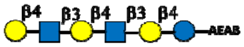	1072.96
121	GLYRDC_023	Galβ1-4GlcNAcβ1-3Galβ1-4GlcNAcβ1-3Galβ1-4GlcNAcβ1-3Galβ1-4Glc-AEAB	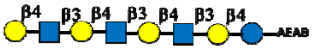	1438.29
122	GLYRDC_127	Fucα1-2Galβ1-3GalNAcβ1-3Gal -AEAB	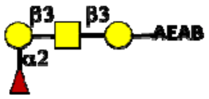	691.62
123	GLYRDC_0331	Fucα1-2Galβ1-3GlcNAcβ1-3Galβ1-4Glc-AEAB	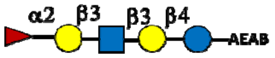	853.76
124	GLYRDC_0332	Fucα1-2Galβ1-4GlcNAcβ1-3Galβ1-4Glc-AEAB	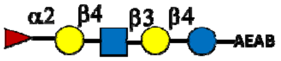	853.76
125	GLYRDC_128	GalNAcα1-3(Fucα1-2)Galβ1-3GalNAcβ1-3Gal-AEAB	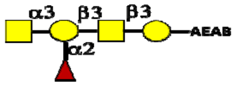	894.82
126	GLYRDC_0371	GalNAcα1-3(Fucα1-2)Galβ1-3GlcNAcβ1-3Galβ1-4Glc -AEAB	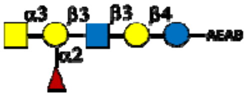	1056.96
127	GLYRDC_0372	GalNAcα1-3(Fucα1-2)Galβ1-4GlcNAcβ1-3Galβ1-4Glc-AEAB	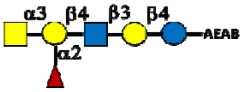	1056.96
128	GLYRDC_0384	Galα1-3(Fucα1-2)Galβ1-3(Fucα1-4)GlcNAc-AEAB	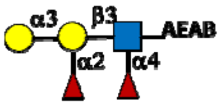	837.77
129	GLYRDC_0385	Galα1-3(Fucα1-2)Galβ1-4(Fucα1-3)GlcNAc -AEAB	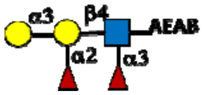	837.77

130	GLYRDC_129	Galα1-3(Fucα1-2)Galβ1-3GalNAcβ1-3Gal-AEAB	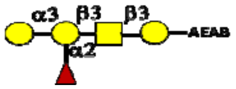	853.76
131	GLYRDC_0401	Galα1-3(Fucα1-2)Galβ1-3GlcNAcβ1-3Galβ1-4Glc-AEAB	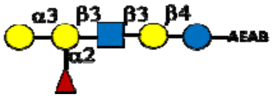	1015.9
132	GLYRDC_0402	Galα1-3(Fucα1-2)Galβ1-4GlcNAcβ1-3Galβ1-4Glc-AEAB	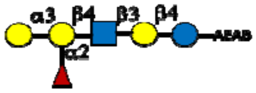	1015.9
133	GLYRDC_051	Galβ1-4(Fucα1-3)GlcNAcβ1-3Galβ1-4(Fucα1-3)Glc-AEAB	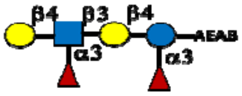	999.9
134	GLYRDC_052	Fucα1-2Galβ1-4(Fucα1-3)GlcNAcβ1-3Gal -AEAB	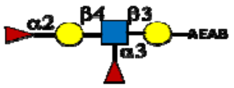	837.77
135	GLYRDC_054	Galβ1-3(Fucα1-4)GlcNAcβ1-3Gal-AEAB	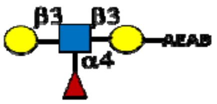	691.62
136	GLYRDC_055	Galβ1-3(Fucα1-4)GlcNAcβ1-3Galβ1-4(Fucα1-3)Glc -AEAB	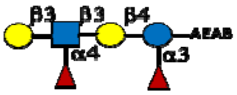	999.9
137	GLYRDC_056	Fucα1-2Galβ1-3(Fucα1-4)GlcNAcβ1-3Gal -AEAB	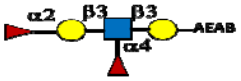	837.77
138	GLYRDC_047	Neu5Acα2-3Galβ1-4(Fucα1-3)GlcNAc-AEAB	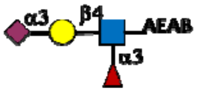	842.72
139	GLYRDC_053	Neu5Acα2-3Galβ1-4(Fucα1-3)GlcNAcβ1-3Gal-AEAB	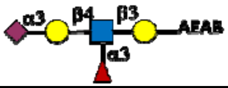	1004.86
140	GLYRDC_066	Fucα1-2Galβ1-4(Fucα1-3)Glc-AEAB	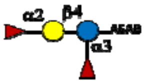	634.57
141	GLYRDC_061	Galβ1-3GlcNAcβ1-3Galβ1-4(Fucα1-3)Glc-AEAB	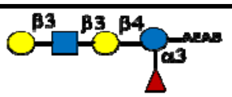	853.76
142	GLYRDC_062	Neu5Acα-3Galβ1-4(Fucα1-3)Glc-AEAB	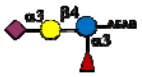	801.67
143	GLYRDC_063	Galβ1-4GlcNAcβ1-3Galβ1-4(Fucα1-3)Glc-AEAB	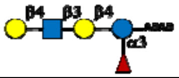	853.76
144	GLYRDC_064	Galα1-3Galβ1-4(Fucα1-3)Glc-AEAB	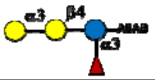	650.57
145	GLYRDC_067	GalNAcα1-3(Fucα1-2)Galβ1-4(Fucα1-3)Glc-AEAB	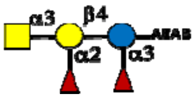	837.77

146	GLYRDC_068	Galα1-3(Fucα1-2)Galβ1-4(Fucα1-3)Glc-AEAB	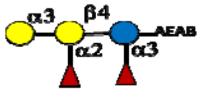	796.71
147	GLYRDC_0741	Galα1-3Galβ1-3GlcNAc -AEAB	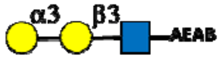	545.48
148	GLYRDC_0742	Galα1-3Galβ1-4GlcNAc -AEAB	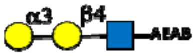	545.48
149	GLYRDC_077	Galα1-3[Galβ1-4GlcNAcβ1-3]2Galβ1-4Glc-AEAB	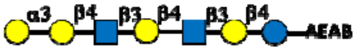	1235.09
150	GLYRDC_078	Galα1-3[Galβ1-4GlcNAcβ1-3]3Galβ1-4Glc-AEAB	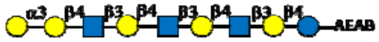	1600.42
151	GLYRDC_079	Galα1-3[Galβ1-4GlcNAcβ1-3]4Galβ1-4Glc -AEAB	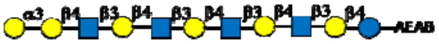	1964.76
152	GLYRDC_075	Galα1-3Galβ1-4(Fucα1-3)GlcNAc -AEAB	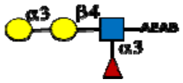	691.62
153	GLYRDC_070	Galα1-3Galβ1-4Glc -AEAB	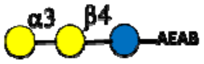	504.43
154	GLYRDC_072	GalNAcβ1-3Galα1-3Galβ1-4Glc -AEAB	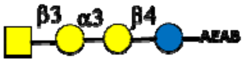	707.62
155	GLYRDC_073	Galβ1-3GalNAcβ1-3Galα1-3Galβ1-4Glc-AEAB	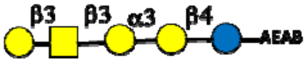	869.76
156	GLYRDC_134	GalNAcα1-3GalNAcβ1-3Galα1-3Galβ1-4Glc -AEAB	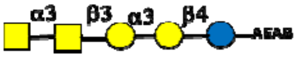	910.82
157	GLYRDC_080	Neu5Acα2-3Galβ1-3GlcNAcβ1-3Gal-AEAB	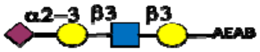	858.72
158	GLYRDC_081	Neu5Acα2-3Galβ1-3GlcNAcβ1-3Galβ1-4Glc -AEAB	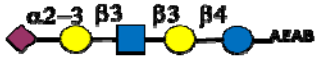	1020.86
159	GLYRDC_082	Neu5Acα2-3Galβ1-4GlcNAcβ1-3Gal-AEAB	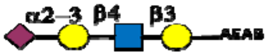	858.72
160	GLYRDC_083	Neu5Acα2-3Galβ1-4GlcNAcβ1-3Galβ1-4Glc -AEAB	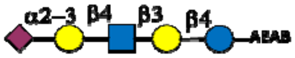	1020.86
161	GLYRDC_090	Neu5Acα2-3Galβ1-4Glc-AEAB	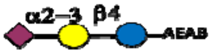	655.53
162	GLYRDC_091	Neu5Acα2-8Neu5Acα2-3Galβ1-4Glc-AEAB	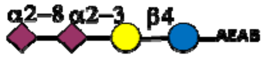	968.76
163	GLYRDC_092	Neu5Acα2-8Neu5Acβ2-8Neu5Acα2-3Galβ1-4Glc -AEAB	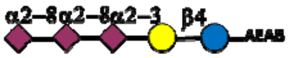	1282

164	GLYRDC_093	GalNAcβ1-4(Neu5Acα2-3)Galβ1-4Glc-AEAB	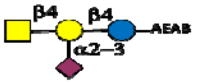	858.72
165	GLYRDC_094	GalNAcβ1-4(Neu5Acα2-8Neu5Acα2-3)Galβ1-4Glc -AEAB	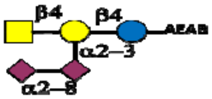	1171.95
166	GLYRDC_095	GalNAcβ1-4(Neu5Acα2-8Neu5Acα2-8Neu5Acα2-3)Galβ1-4Glc-AEAB	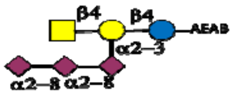	1485.19
167*	GLYRDC_096	Galβ1-3GalNAcβ1-4(Neu5Acα2-3)Galβ1-4Glc-AEAB	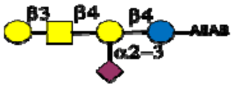	1020.86
168	GLYRDC_097	Neu5Acα2-3Galβ1-3GalNAcβ1-4Galβ1-4Glc-AEAB	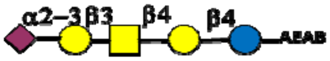	1020.86
169	GLYRDC_098	Neu5Acα2-3Galβ1-3GalNAcβ1-4(Neu5Acα2-3)Galβ1-4Glc -AEAB	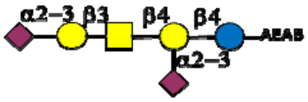	1334.09
170	GLYRDC_099	Galβ1-3GalNAcβ1-4(Neu5Acα2-8Neu5Acα2-3)Galβ1-4Glc-AEAB	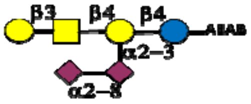	1334.09
171	GLYRDC_102	Galβ1-3GalNAcβ1-4Galβ1-4Glc-AEAB	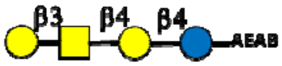	707.62
172	GLYRDC_103	Fucα1-2Galβ1-3GalNAcβ1-4(Neu5Acα2-3)Galβ1-4Glc-AEAB	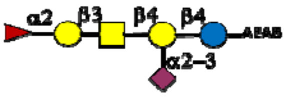	1168
173	GLYRDC_104	GalNAcβ1-4Galβ1-4Glc-AEAB	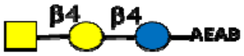	545.48
174	GLYRDC_120	Galα1-4Galβ1-4Glc-AEAB	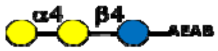	504.43
175	GLYRDC_121	GalNAcβ1-3Galα1-4Galβ1-4Glc-AEAB	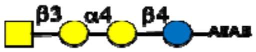	707.62
176	GLYRDC_136	Galα1-4Galβ1-4GlcNAc-AEAB	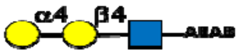	545.48
177	GLYRDC_171	Neu5Acα2-3Galα1-4Galβ1-4Glc-AEAB	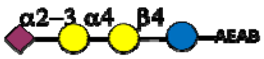	817.67
178	GLYRDC_124	GalNAcα1-3(Fucα1-2)Galβ1-3GalNAcβ1-3Galα1-4Galβ1-4Glc-AEAB	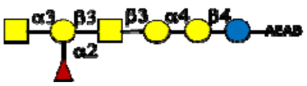	1219.09
179	GLYRDC_122	Galβ1-3GalNAcβ1-3Galα1-4Galβ1-4Glc -AEAB	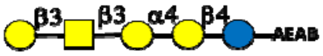	869.76

180	GLYRDC_123	Fucα1-2Galβ1-3GalNAcβ1-3Galα1-4Galβ1-4Glc -AEAB	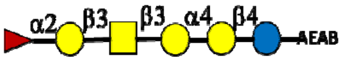	1015.9
181	GLYRDC_130	Neu5Acα2-3Galβ1-3GalNAcβ1-3Gal-AEAB	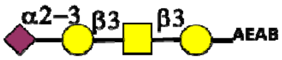	858.72
182	GLYRDC_131	Neu5Acα2-3Galβ1-3GalNAcβ1-3Galα1-4Galβ1-4Glc-AEAB	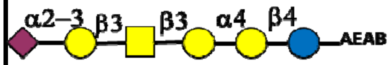	1183
183	GLYRDC_132	GalNAcα1-3GalNAcβ1-3Galα1-4Galβ1-4Glc-AEAB	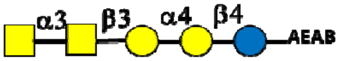	910.82
184	GLYRDC_133	GalNAcα1-3GalNAcβ1-3Gal-AEAB	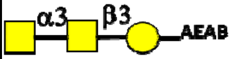	586.54
185	GLYRDC_180	GlcAβ1-3Galβ1-4Glc-AEAB	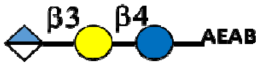	540
186	GLYRDC_181	GlcAβ1-3Galβ1-3GlcNAcβ1-3Galβ1-4Glc-AEAB	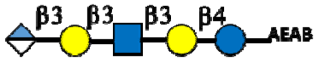	883.75
187	GLYRDC_163	Fucα1-2Galβ1-4(Fucα1-2)Glc -AEAB	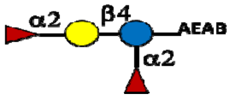	634.57
188	GLYRDC_164	GalNAcα1-3(Fucα1-2)Galβ1-4(Fucα1-2)Glc-AEAB	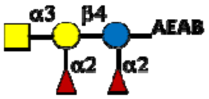	837.77
189	GLYRDC_1911	Galβ1-3GlcNAcβ1-3Galα1-4Galβ1-4Glc-AEAB	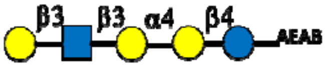	869.76
190	GLYRDC_1912	Galβ1-4GlcNAcβ1-3Galα1-4Galβ1-4Glc-AEAB	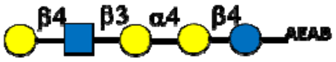	869.76
191	GLYRDC_1951	NeuAcα2-3Galβ1-3GlcNAcβ1-3Galα1-4Galβ1-4Glc-AEAB	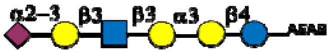	1183
192	GLYRDC_2011	Fucα1-2Galβ1-3GlcNAcβ1-3Galα1-3Galβ1-4Glc-AEAB	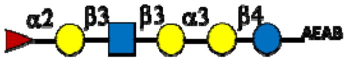	1015.9
193	GLYRDC_111	Galβ1-4(Neu5Acα2-3)Galβ1-4Glc-AEAB	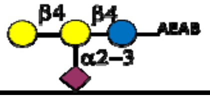	817.67
194	GLYRDC_174	Galβ1-3GalNAcβ1-4Galβ1-3GalNAcβ1-4Galβ1-4Glc-AEAB	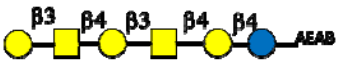	1072.95
195	GLYRDC_GLU314	Maltotetraose DP4-AEAB	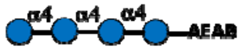	666.57

196	GLYRDC_GLU315	Maltopentaose DP5 -AEAB	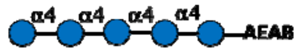	828.71
197	GLYRDC_GLU316	Maltohexaose DP6-AEAB	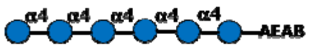	990.85
198	GLYRDC_GLU317	Maltoheptaose DP7 -AEAB	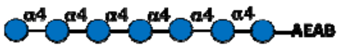	1152.99
199	GLYRDC_GLU318	Maltooctaose DP8 -AEAB	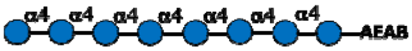	1315.13
200	GLYRDC_GLU319	Maltononaose DP9 -AEAB	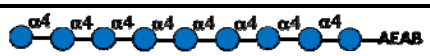	1477.27

Gly#109 was analyzed in OvCaR Samples for anti-glycan IgG

Gly#167 was analyzed for anti-Glycan IgM in type-1 diabetes samples.

Sp: $OCH_2CH_2CH_2NH_2$
Sp1: $NH(CH3)OCH_2CH_2NH_2$
Sp2: $O(CH_2)_3NHCOCH_2(OCH_2CH_2)_5CH_2CH_2NH_2$
AEAB: 2,6-diaminopyridine (DAP) and 2-amino(N-aminoethyl) benzamide

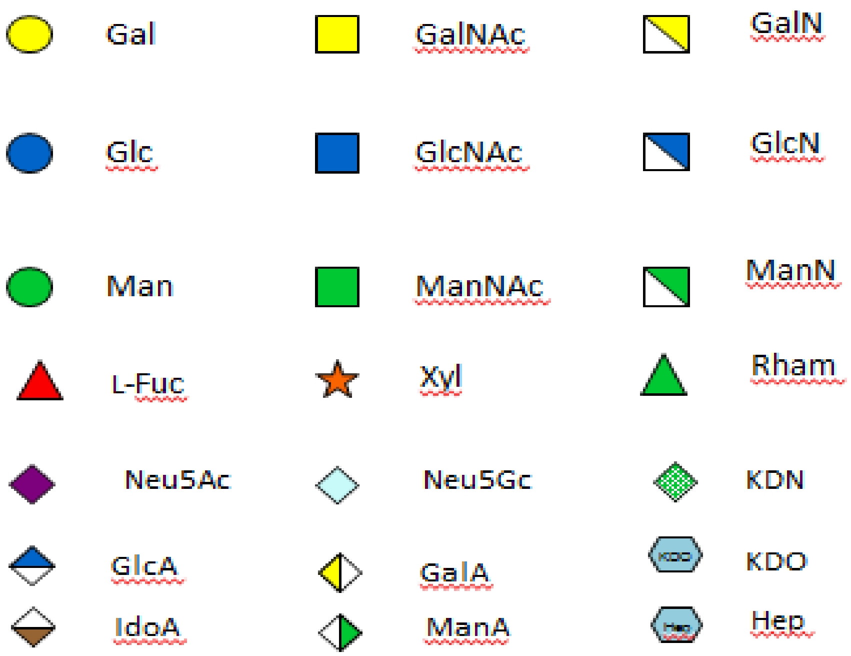

Supplementary Table 2: List of lectins used for characterization of the multiplex glycan bead array.

Lectin	Acronym	Sugar Specificity
Ricinus communis II	RCAII	Gal, GalNAc
Erythrina cristagali	ECL	Galβ4GlcNAc
Succinylated Wheat Germ	SuWGA	GlcNAc
Lens clunaris	LCA	αMan,αGlc
Ricinus communis I	RCAI	Gal
Jacalin	Jacalin	Galβ3GalNAc
Pisium sativum	PSA	αMan,αGlc
Succinylated Concanavalin A	SuConA	αMan,αGlc
Soyabean	SBA	α>βGalNAc
Dolichos biflorus	DBA	αGalNAc
Galanthus nivalis	GNL	αMan
Psophocarpus tetragonolobus I	PTLI	GalNAc, Gal
Phaseolus vulgaris E	PHA-E	bisecting Galβ1-4GlcNAcβ1-2Man
Lycopersicon esculentum	LEL	(GlcNAc)2-4
Maackia amurensisI	MALI	Galβ4GlcNAc
Vicia villosa	VVL	GalNAc
Solanum tuberosum	STL	(GlcNAc)2-4
Phaseolus vulgaris L	PHA-L	
Amaranthus caudatus	ACL	Galβ3GalNAc
Peanut	PNA	Galβ3GalNAc
Ulex europaeus	UEA	αFuc
Narcissus pseudonarcissus	NPL	αMan
Euonymus europaeus	EEL	Galα3Gal
Griffonia simplicifolia II	GSLII	α or βGlcNAc
Lotus tetragonolobus	LTL	αFuc
Sambucus nigra	SNA	Neu5Acα6Gal/GalNAc
Bauhinia purpurea	BPL	Galβ3GalNAc
Hippeastrum hybrid	HHL	αMan
Musa Paradisiaca	MPL	αMan,αGlc
Sophora japonica	SJA	βGalNAc
Datura Stramonium	DSL	(GlcNAc)2-4
Wisteria Floribunda	WFL	GalNAc
Griffonia simplicifolia I	GSLI	αGal,αGalNAc
Concanavalin A	ConA	αMan,αGlc
Psophocarpus tetragonolobusII	PTLII	GalNAc,Gal
Griffonia simplicfolia I isolectin B4	BSIB4	αGal
Maackia amurensisII	MALII	Neu5Acα3Galβ3GalNAc
Aleuria aurantia	AAL	Fucα6GlcNac
Wheat Germ	WGA	GlcNAc

Supplementary Table 3: Commercial and in house raised anti-glycan antibodies used to characterized suspension glycan array.

Anti-Glycan antibody	Company	Clone ID	Cat#	Lot No	Ig Type
CA19.9	ABCAM		ab3982	GR72650-15	IgM
Le^x	ABCAM		ab3358	GR158608-1	IgM
Le^y IgM	ABCAM		ab3359	GR147932-9	IgM
SSEA3	In Lab*	MC631			IgM
GM1	In Lab*	D0131			IgM
Gal α1,3	In Lab*				IgM
Sle^x	In Lab*	SNH3			IgM
GA1	In Lab*	D079			IgM
Le^b	ABCAM		ab3968	GR133794-1	IgG
Blood Group H2	ABCAM		ab33404	GR174650-1	IgG
CA19.9	US Bio	5G17	C0075-31	L12102625	IgG
Le^a	ABCAM		ab3967	GR117120-8	IgG
Le^Y IgG	In Lab*	E2014			IgG
Muc1	Fitzgerald	M3A106	10-R-M129C	1714	IgG
Muc1	BBI solutions		BM236-V2G9	NA	IgG
mouse anti-Goat IgG HRP	Pierce	Poly clonal			IgG
Rat IgG	Pierce				IgG

Muc1, Rat IgG and mouse anti-goat antibodies were used as negative control.

All antibodies were used at a dilution of 1:100 in 1% BSA in PBS (w/v).

*These monoclonal antibodies were produced in laboratory by immunizing intact cells into mice. Individual clones were identified by inhibition studies (unpublished results).

NA: Not Available

Supplementary Table 4: Frequency and percentage positive samples for anti-glycan IgM antibody in human subjects (n=613).

Glycan IDs	Glycan	Below Cutoff (n)	Above Cutoff (n)	Percentage Positive	Cutoff MFI (Mean+3SD)
2	GLYPW_002	274	339	55.3	587
4	GLYPW_004	352	260	42.5	60
5	GLYPW_005	612	0	0	41
6	GLYPW_006	7	605	98.9	51
7	GLYPW_007	137	475	77.6	59
8	GLYPW_008	326	286	46.7	38
9	GLYPW_009	568	45	7.3	67
10	GLYPW_010	12	600	98	74
11	GLYPW_011	158	455	74.2	54
13	GLYPW_016	35	577	94.3	62
14	GLYPW_017	6	606	99	61
15	GLYPW_018	105	507	82.8	121
16	GLYPW_019	50	563	91.8	59
17	GLYPW_020	95	517	84.5	52
18	GLYPW_021	38	575	93.8	41
19	GLYPW_022	44	568	92.8	70
20	GLYPW_023	11	602	98.2	45
21	GLYPW_024	477	135	22.1	46
22	GLYPW_025	612	0	0	43
23	GLYPW_026	450	163	26.6	45
24	GLYPW_027	572	41	6.7	37
25	GLYPW_028	500	113	18.4	40
26	GLYPW_029	564	48	7.8	55
27	GLYPW_030	256	357	58.2	35
28	GLYPW_031	522	90	14.7	30
29	GLYPW_032	187	426	69.5	33
30	GLYPW_033	567	45	7.4	62
31	GLYPW_034	598	14	2.3	32
32	GLYPW_035	613	0	0	57
33	GLYPW_036	613	0	0	26
34	GLYPW_037	585	27	4.4	35
43	GLYPW_046	45	568	92.7	72
44	GLYPW_047	68	544	88.9	65
45	GLYPW_048	450	163	26.6	53
46	GLYPW_049	613	0	0	55
47	GLYPW_050	114	499	81.4	38
48	GLYPW_051	24	587	96.1	79
52	GLYPW_055	425	187	30.6	60

54	GLYPW_057	12	601	98	75
55	GLYPW_058	141	472	77	38
56	GLYPW_059	193	420	68.5	44
57	GLYPW_060	360	253	41.3	43
58	GLYPW_061	294	319	52	41
59	GLYPW_062	104	509	83	39
60	GLYPW_063	319	294	48	48
61	GLYPW_064	322	291	47.5	39
62	GLYPW_065	279	334	54.5	77
63	GLYPW_066	17	596	97.2	36
64	GLYPW_067	613	0	0	35
65	GLYPW_068	596	17	2.8	37
66	GLYPW_069	613	0	0	33
67	GLYPW_070	613	0	0	22
68	GLYPW_071	610	3	0.5	32
69	GLYPW_072	136	477	77.8	42
70	GLYPW_073	210	403	65.7	32
71	GLYPW_074	128	485	79.1	35
72	GLYPW_075	244	369	60.2	38
73	GLYPW_076	91	522	85.2	28
74	GLYPW_077	67	546	89.1	41
75	GLYPW_078	131	482	78.6	31
76	GLYPW_079	154	459	74.9	42
77	GLYPW_080	185	428	69.8	21
78	GLYPW_081	180	433	70.6	39
79	GLYPW_082	173	440	71.8	32
80	GLYPW_083	237	376	61.3	36
81	GLYPW_084	82	531	86.6	21
82	GLYPW_085	83	530	86.5	30
83	GLYPW_086	202	411	67	38
84	GLYPW_087	6	607	99	35
85	GLYPW_088	232	381	62.2	32
86	GLYPW_089	58	555	90.5	33
87	GLYPW_090	21	592	96.6	35
88	GLYPW_091	179	434	70.8	30
89	GLYPW_092	613	0	0	33
90	GLYPW_093	603	10	1.6	40
91	GLYPW_094	525	88	14.4	38
92	GLYPW_095	213	400	65.3	31
93	GLYPW_096	416	197	32.1	38
94	GLYPW_097	606	7	1.1	40
95	GLYPW_098	613	0	0	24
96	GLYPW_099	582	31	5.1	52

97	GLYPW_100	409	204	33.3	70
98	GLYPW_101	605	8	1.3	58
99	GLYPW_102	480	133	21.7	61
100	GLYPW_103	613	0	0	28
101	GLYPW_104	368	245	40	47
102	GLYPW_105	591	21	3.4	69
103	GLYPW_106	491	122	19.9	43
104	GLYPW_107	324	289	47.1	168
105	GLYPW_108	590	22	3.6	44
106	GLYPW_109	610	2	0.3	39
107	GLYPW_110	605	7	1.1	57
108	GLYPW_111	487	126	20.6	54
110	GLYPW_113	589	23	3.8	37
111	GLYPW_114	612	0	0	65
112	GLYPW_115	32	580	94.8	45
113	GLYPW_116	282	330	53.9	63
114	GLYPW_117	109	503	82.2	64
115	GLYPW_118	356	256	41.8	30
116	GLYPW_119	415	197	32.2	44
117	GLYPW_120	612	0	0	49
118	GLYPW_121	305	307	50.2	47
119	GLYRDC_126	161	452	73.7	25
120	GLYRDC_022	28	585	95.4	24
121	GLYRDC_023	105	508	82.9	39
122	GLYRDC_127	556	57	9.3	33
123	GLYRDC_331	20	593	96.7	30
124	GLYRDC_332	17	596	97.2	22
125	GLYRDC_128	512	101	16.5	34
126	GLYRDC_371	361	252	41.1	27
127	GLYRDC_372	41	572	93.3	36
128	GLYRDC_384	472	141	23	29
129	GLYRDC_385	20	593	96.7	27
130	GLYRDC_129	293	320	52.2	31
131	GLYRDC_401	147	466	76	33
132	GLYRDC_402	18	595	97.1	38
133	GLYRDC_051	589	24	3.9	33
134	GLYRDC_052	554	59	9.6	32
135	GLYRDC_054	500	113	18.4	27
136	GLYRDC_055	16	597	97.4	55
137	GLYRDC_056	583	30	4.9	31
138	GLYRDC_047	368	245	40	26
139	GLYRDC_053	178	435	71	28
140	GLYRDC_066	370	243	39.6	26

141	GLYRDC_061	553	60	9.8	38
142	GLYRDC_062	166	447	72.9	27
143	GLYRDC_063	521	92	15	43
144	GLYRDC_064	219	394	64.3	27
145	GLYRDC_067	403	210	34.3	31
146	GLYRDC_068	420	193	31.5	32
147	GLYRDC_741	96	517	84.3	26
148	GLYRDC_742	367	246	40.1	32
149	GLYRDC_077	163	450	73.4	23
150	GLYRDC_078	339	274	44.7	31
151	GLYRDC_079	8	605	98.7	32
152	GLYRDC_075	138	475	77.5	30
153	GLYRDC_070	339	274	44.7	40
154	GLYRDC_072	215	398	64.9	36
155	GLYRDC_073	32	581	94.8	27
156	GLYRDC_134	4	609	99.3	20
157	GLYRDC_080	497	116	18.9	30
158	GLYRDC_081	464	149	24.3	26
159	GLYRDC_082	417	196	32	23
160	GLYRDC_083	593	20	3.3	32
161	GLYRDC_090	599	14	2.3	27
162	GLYRDC_091	495	118	19.2	22
163	GLYRDC_092	544	69	11.3	51
164	GLYRDC_093	364	249	40.6	33
165	GLYRDC_094	610	3	0.5	33
166	GLYRDC_095	362	251	40.9	31
167	GLYRDC_096	205	408	66.6	36
168	GLYRDC_097	308	305	49.8	34
169	GLYRDC_098	541	72	11.7	25
170	GLYRDC_099	186	427	69.7	27
171	GLYRDC_102	73	540	88.1	32
172	GLYRDC_103	338	275	44.9	22
173	GLYRDC_104	162	451	73.6	34
174	GLYRDC_120	357	256	41.8	44
175	GLYRDC_121	301	312	50.9	38
176	GLYRDC_136	444	169	27.6	36
177	GLYRDC_171	514	99	16.2	48
178	GLYRDC_124	459	154	25.1	35
179	GLYRDC_122	333	280	45.7	57
180	GLYRDC_123	564	49	8	49
181	GLYRDC_130	315	298	48.6	14
182	GLYRDC_131	576	37	6	29
183	GLYRDC_132	41	572	93.3	36

184	GLYRDC_133	39	574	93.6	31
185	GLYRDC_180	227	386	63	38
186	GLYRDC_181	570	43	7	43
187	GLYRDC_163	320	293	47.8	32
188	GLYRDC_164	445	168	27.4	33
189	GLYRDC_1911	261	352	57.4	32
190	GLYRDC_1912	274	339	55.3	33
191	GLYRDC_1951	313	300	48.9	33
192	GLYRDC_2011	365	248	40.5	28
193	GLYRDC_111	588	25	4.1	24
194	GLYRDC_174	540	73	11.9	26
195	GLYRDC_GLU314	403	210	34.3	44
196	GLYRDC_GLU315	389	224	36.5	30
197	GLYRDC_GLU316	581	32	5.2	34
198	GLYRDC_GLU317	543	70	11.4	33
199	GLYRDC_GLU318	373	240	39.2	29
200	GLYRDC_GLU319	589	24	3.9	42

Cutoff value is based on the mean + 3SD of blank controls in quadruplicate (without serum).

A sample is considered positive if net (background subtracted) MFI is above the mean+3SD of blank controls.

MFI: Median Fluorescence Intensities
