# Supplementary material for: Multiplex glycan bead array for high throughput and high content analyses of glycan binding proteins
Source: Nat Commun. 2018 Jan 17;9:258. doi: 10.1038/s41467-017-02747-y (PMC5772357; doi:10.1038/s41467-017-02747-y)
Supplement: Supplementary file 2 — Descriptions of Additional Supplementary Files [file 41467_2017_2747_MOESM2_ESM.pdf]

Descriptions of Additional Supplementary Files

File Name: Supplementary Dataset 1
Description: Median fluorescence intensities obtained for the glycan array for 39 lectins and control proteins. Duplicates are indicated by the lectin/protein name followed by ".1". Four blank controls (without lectin or protein) were indicated as B, B.1, B.2 and B.3.
